# Distinct patterns of responses in endothelial cells and smooth muscle cells following vascular injury

**DOI:** 10.1172/jci.insight.153769

**Published:** 2022-10-24

**Authors:** Xili Ding, Qin An, Weikang Zhao, Yang Song, Xiaokai Tang, Jing Wang, Chih-Chiang Chang, Gexin Zhao, Tzung Hsiai, Guoping Fan, Yubo Fan, Song Li

**Affiliations:** 1 Department of Bioengineering, University of California, Los Angeles, California, USA.; 2 School of Engineering Medicine and; 3 Key Laboratory for Biomechanics and Mechanobiology of Ministry of Education, Beijing Advanced Innovation Center for Biomedical Engineering, School of Biological Science and Medical Engineering, Beihang University, Beijing, China.; 4 Department of Human Genetics, David Geffen School of Medicine,; 5 Department of Pathology and Laboratory Medicine, David Geffen School of Medicine, and; 6 Department of Medicine, University of California, Los Angeles, California, USA.

**Keywords:** Vascular Biology, Cardiovascular disease

## Abstract

Vascular smooth muscle cells (SMCs) are heterogeneous, and their differential responses to vascular injury are not well understood. To address this question, we performed single-cell analysis of vascular cells to a ligation injury in mouse carotid arteries after 3 days. While endothelial cells had a homogeneous activation of mesenchymal genes, less than 30% of SMCs responded to the injury and generated 2 distinct clusters — i.e., proinflammatory SMCs and stress-responsive SMCs. Proinflammatory SMCs were enriched with high levels of inflammatory markers such as vascular cell adhesion molecule-1 while stress-responsive SMCs overexpressed heat shock proteins. Trajectory analysis suggested that proinflammatory SMCs were potentially derived from a specific subpopulation of SMCs. Ligand-receptor pair analysis showed that the interaction between macrophages and proinflammatory SMCs was the major cell-cell communication among all cell types in the injured arteries. In vitro coculture demonstrated that VCAM1^+^ SMCs had a stronger chemotactic effect on macrophage recruitment than VCAM1^–^ SMCs. Consistently, the number of VCAM1^+^ SMCs significantly increased in injured arteries and atherosclerotic lesions of ApoE^–/–^ mice and human arteries. These findings provide insights at the single-cell level on the distinct patterns of endothelial cells and SMC responses to vascular injury.

## Graphical abstract

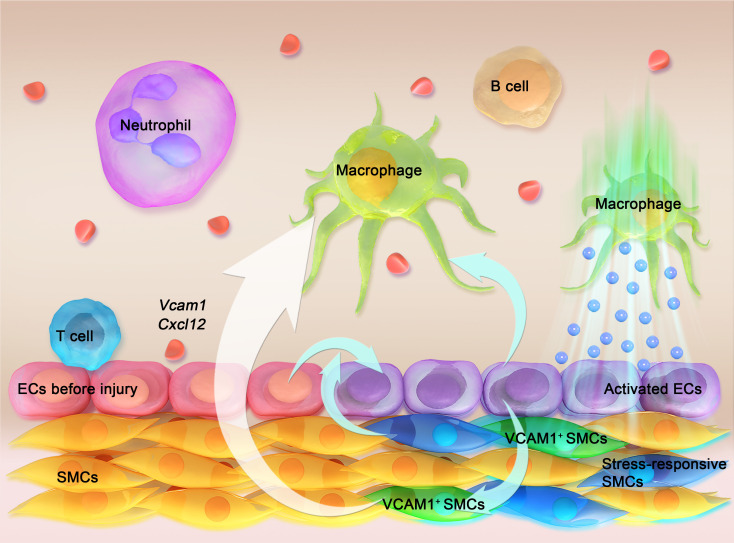

## Introduction

Vascular diseases, such as atherosclerosis and restenosis, can lead to myocardial infarction, ischemic stroke, renal impairment, and aneurysms with hypertension (1, 2) and are leading causes of morbidity and mortality in the world (3). The injury and activation of endothelial cells (ECs), the phenotype changes of smooth muscle cells (SMCs), and the recruitment of inflammatory cells are critical events in neointimal formation and disease development (4–8). SMCs are heterogeneous in their developmental origins (9). It has been shown that SMCs can dedifferentiate into proliferative and/or synthetic phenotypes in response to vascular injury (10–14); however, SMC plasticity may be limited to subpopulations of SMCs, and it is not clear how the intrinsic differences among SMCs lead to differential responses to vascular injury. Traditional histological analysis of SMC phenotypes relies on the expression of a few SMC markers, while gene expression analysis of SMCs as a population only provides the average expression profile of heterogeneous SMCs and masks the differential responses of individual cells.

Technological advances in single-cell RNA-Seq (scRNA-Seq) provide a powerful tool to identify cell types within a complex tissue and characterize each cell at the genomic level (15–18). Recent studies using scRNA-Seq revealed heterogeneous cell phenotypes in adventitia and SMCs in atherosclerotic lesions (19, 20). However, there is a lack of understanding on how SMC heterogeneity contributes to the early responses of SMCs to vascular injury at the single-cell level. In this study, we used the droplet-based scRNA-Seq method to characterize the transcriptomes of thousands of individual cells collected from the normal arteries and the arteries with ligation injury and investigate the early responses of vascular cells and inflammatory cells to the injury. The single-cell analysis revealed that, while almost all ECs had the same phenotypic transition, less than 30% of SMCs responded to the injury and generated 2 new clusters of SMCs; a proinflammatory SMC subpopulation with a chemotactic effect on macrophages and stress-responsive SMCs with high expression levels of heat shock proteins. These findings unravel the distinct patterns of EC and SMC responses to injury, shed light on how the intrinsic SMC heterogeneity contributes to the differential responses of SMCs, and underscore the importance of targeting a specific subpopulation of SMCs for vascular disease therapy.

## Results

### Unbiased clustering of scRNA-Seq data revealed major cell types in mouse carotid arteries following injury.

**Figure 1:**
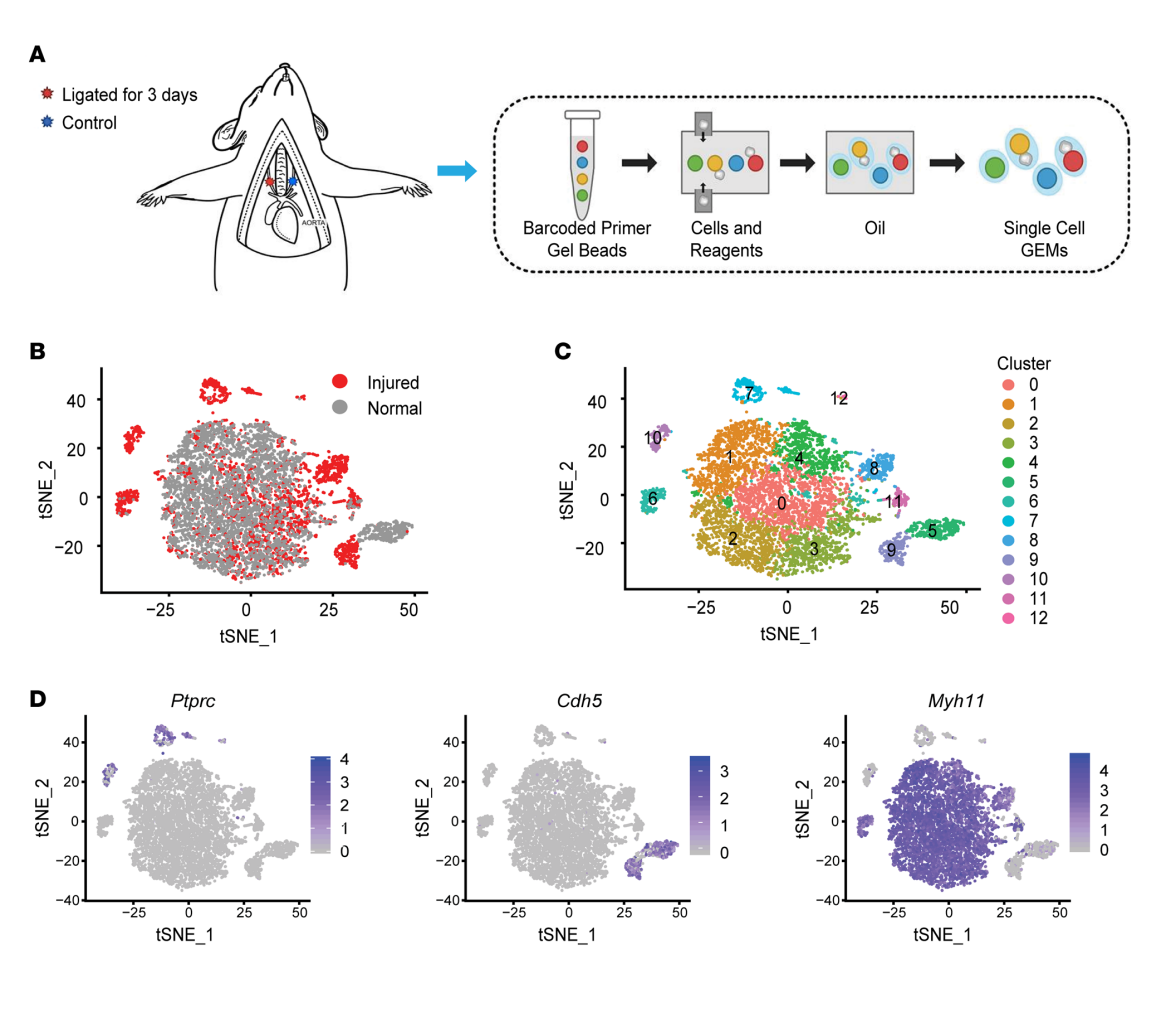
scRNA-Seq of cells from normal and injured carotid artery. (**A**) Schematic diagram of the experimental procedure. Carotid artery ligation was performed on 2-month-old mice, which were sacrificed 3 days after the ligation procedure. Medial and intimal layers of both normal and injured carotid arteries were dissected out and enzymatically digested to generate single-cell suspensions. The transcriptome of individual cells was profiled using 10X Genomics platform. (**B**) t-SNE map of medial and intimal cells from normal and injured arteries. (**C**) t-SNE representing the cell clusters identified using the unbiased clustering algorithm. Cells were colored by their cluster identity. (**D**) t-SNE map showing the expression of representative marker genes, including inflammatory marker *Ptprc*, EC marker *Cdh5*, and SMC marker *Myh11*.

First, we performed carotid artery ligation to induce vascular injury in the mouse model (6, 21). To determine the cell responses at the early stage, we harvested the arteries at day 3 after the ligation. With a focus on the cells in intima and media layers, we removed the adventitia tissue, enzymatically dissociated media and intimal cells, and profiled their transcriptome using 10X Genomics scRNA-Seq (Figure 1A). After the quality control step, we obtained 4756 and 3605 single cells from normal or injured arteries, respectively (Supplemental Figure 1; supplemental material available online with this article; https://doi.org/10.1172/jci.insight.153769DS1). These cells were then merged into 1 data set and analyzed together using Seurat V3 software package (Figure 1B). Unbiased clustering using 1970 highly variable genes identified 12 clusters within these cells (Figure 1C). By identifying genes that were highly expressed in each cluster, we classified 12 cell clusters into 3 major cell types, including ECs (*Cdh5^+^*), SMCs (*Myh11^+^*), and immune cells (*Ptprc^+^*) (Figure 1D). Overall, ECs had a relatively homogeneous response, but SMC responses were heterogeneous. The whole EC cluster experienced a phenotypic shift from clusters 5–9. In contrast, most SMCs (clusters 0–4) did not show significant changes, and only a fraction of SMCs shifted to new clusters (clusters 6 and 8). Cluster 11 highly expressed *Myh11* and was a small subpopulation (< 3%) of SMCs similar to clusters 0–4. In addition, immune cells (clusters 7, 10, and 12) that were absent in normal arteries appeared in injured arteries (Table 1).

### EC activation and phenotypic transition.

**Figure 2:**
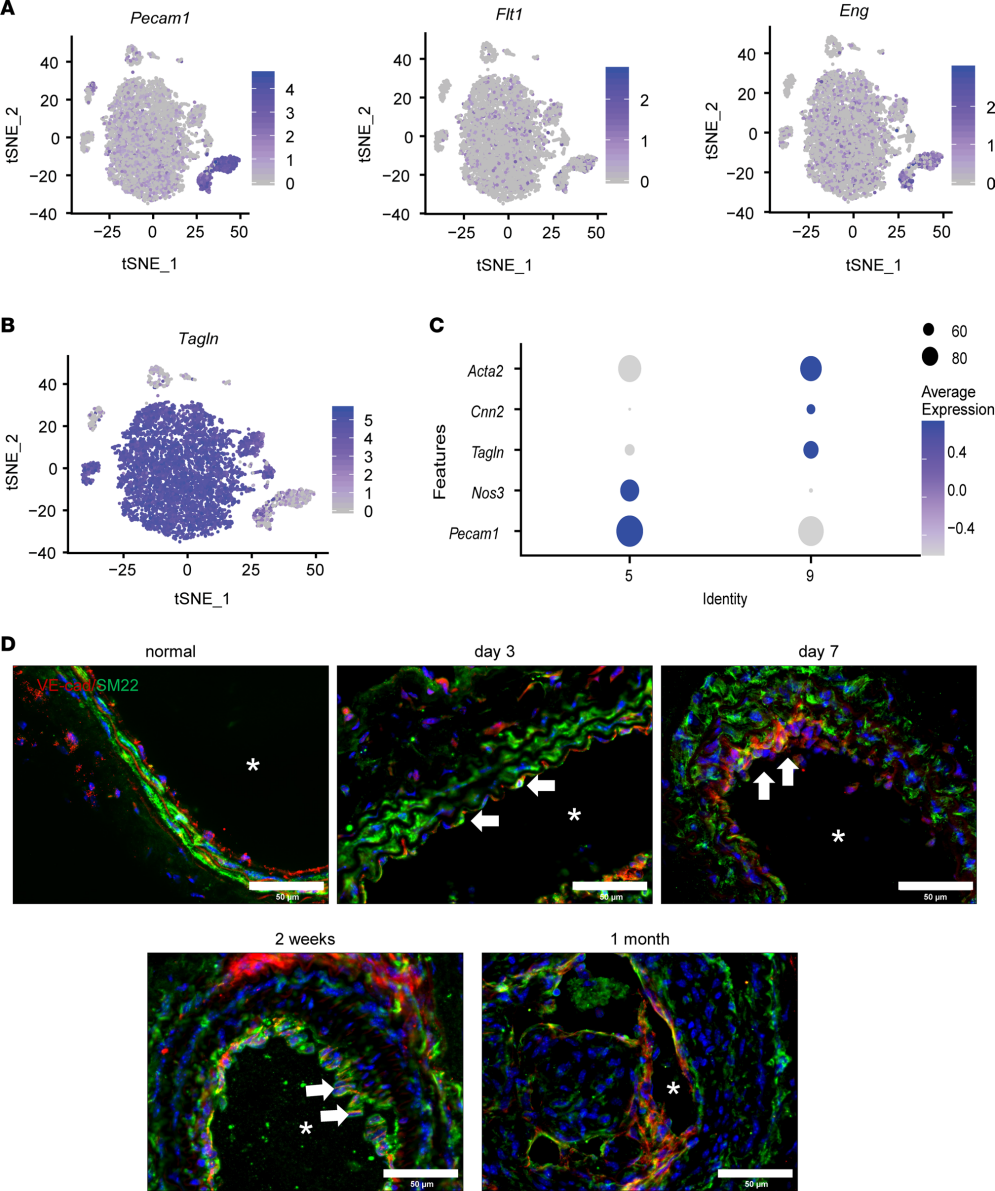
Early responses of ECs with the activation of mesenchymal genes. (**A**) Feature plot of EC markers *Pecam1*, *Flt1*, and *Eng* in normal and injured arteries. (**B**) Feature plot of selected markers *Tagln* in normal and injured arteries. (**C**) Differentially expressed genes between ECs in cluster 5 (normal arteries) and cluster 9 (injured arteries). (**D**) Immunofluorescence staining of VE-cadherin (VE-cad) and SM22 was performed in normal and injured arteries at different time points (3 days to 1 month) after ligation (*n* = 5). Scale bar: 50 μm. Arrows indicate VE-cad^+^SM22^+^ cells. Asterisk indicates the lumen of the artery.

Expression of endothelial markers *Pecam1*, *Flt1*, and *Eng* allowed us to define clusters 5 and 9 as ECs from normal and injured arteries, respectively (Figure 2A). Cluster 5 are ECs in normal arteries, while cluster 9 are ECs in injured arteries, suggesting a homogeneous phenotypic shift of ECs upon injury. We then compared clusters 5 and 9 to identify the genes that were regulated by injury in ECs. We found that 12 genes were significantly induced by injury (multiple-test adjusted *P* value < 0.05, log_2_-transformed fold change > 1), as listed in Supplemental Figure 2. Interestingly, *Tagln* (SM22), an SMC marker, was highly expressed in all ECs after injury (Figure 2B). In addition, the expression of mesenchymal genes such as *Acta2* and *Cnn2* (22) were also upregulated while the expression of endothelial marker genes, such as *Nos3* and *Pecam1* (23), were downregulated (Figure 2C). These results suggested that all ECs were activated following ligation injury (24, 25). To access whether ECs express mesenchymal markers in vivo, we evaluated the expression of SM22 in ECs at different time points after injury. We found that ECs in the normal arteries did not express SM22 and that SM22 was expressed in ECs at 7 days after ligation (Figure 2D). Moreover, most of the ECs express SM22 protein at 2 weeks after ligation (Figure 2D).

### Only a fraction of SMCs responded to ligation injury.

**Figure 3:**
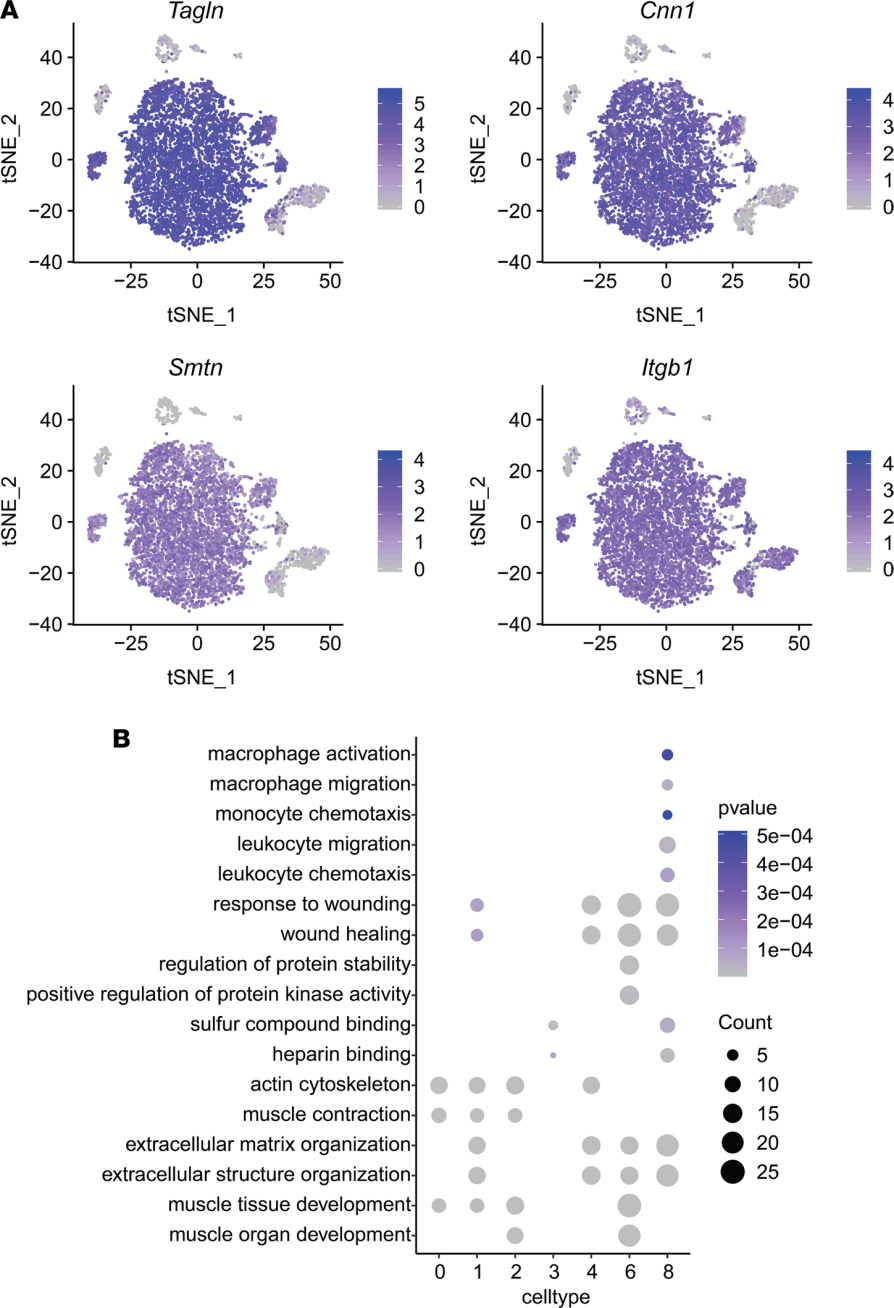
Characteristics of SMC heterogeneity. (**A**) Feature plot of SMC markers *Tagln*, *Cnn1*, S*mtn*, and *Itgb1* in normal and injured arteries. (**B**) GO term (biological pathway) analysis of different SMC clusters (0, 1, 2, 3, 4, 6, and 8).

SMCs accounted for more than 70% of the cells isolated from the normal and injured arteries (Table 1). The expression of multiple SMC genes (*Tagln*, *Cnn1*, *Smtn*, and *Itgb1*) allowed us to identify clusters 0, 1, 2, 3, 4, 6, and 8 as major subpopulations of SMCs (Figure 3A). Most of the SMCs belonged to a large cluster that comprised 5 closely related small clusters of 0–4 (Figure 1, C and D). These 5 clusters were presented in both normal and injured arteries, demonstrating the intrinsic heterogeneity of SMCs. Clusters 0 and 1 accounted for about 50% of all SMCs and did not have significant changes following injury. The percentage of SMCs in clusters 2–4 decreased by about 50% in injured arteries, and cluster 6 (13%) and cluster 8 (11%) were new SMC clusters following injury (Figure 1B and Table 1), representing activated SMCs with distinct phenotypic changes. Of note, S phase genes and G2/M phase genes were not enriched in a specific cluster and the cell cycle analysis did not show significant differences among the different SMC cells clusters (Supplemental Figure 3), suggesting that the proliferative phenotype did not appear at this early stage (day 3) and that clusters 6 and 8 were likely derived from clusters 2–4. These results demonstrated that only a fraction of SMCs responded to ligation injury.

We then identified genes specifically expressed in each SMC cluster. The gene ontology (GO) enrichment analysis of upregulated differentially expressed genes in each SMC cluster is shown in Figure 3B. Actin cytoskeleton and muscle-related pathways are among the top biological pathways identified in clusters 0–2, representing the typical mature SMC populations, while cluster 3 does not have these characteristics. Cluster 4 is more similar to cluster 1 in extracellular matrix (ECM) organization and wound healing pathways but not muscle-related pathways. Cluster 6 is similar to cluster 1 and 4 but has more genes involving in protein stability regulation. Cluster 8 is similar to cluster 4 in ECM organization and wound-healing pathways and has unique characteristics in the modulation of the migration and chemotaxis of macrophages and leukocytes. The highly expressed genes identified in different SMC clusters are listed in Supplemental Table 1. These results suggested that SMCs in normal arteries were heterogeneous and intrinsically different and that only a fraction of SMCs might respond to vascular injury.

### Single-cell trajectory analysis revealed transcriptomic dynamics of SMCs induced by ligation injury.

**Figure 4:**
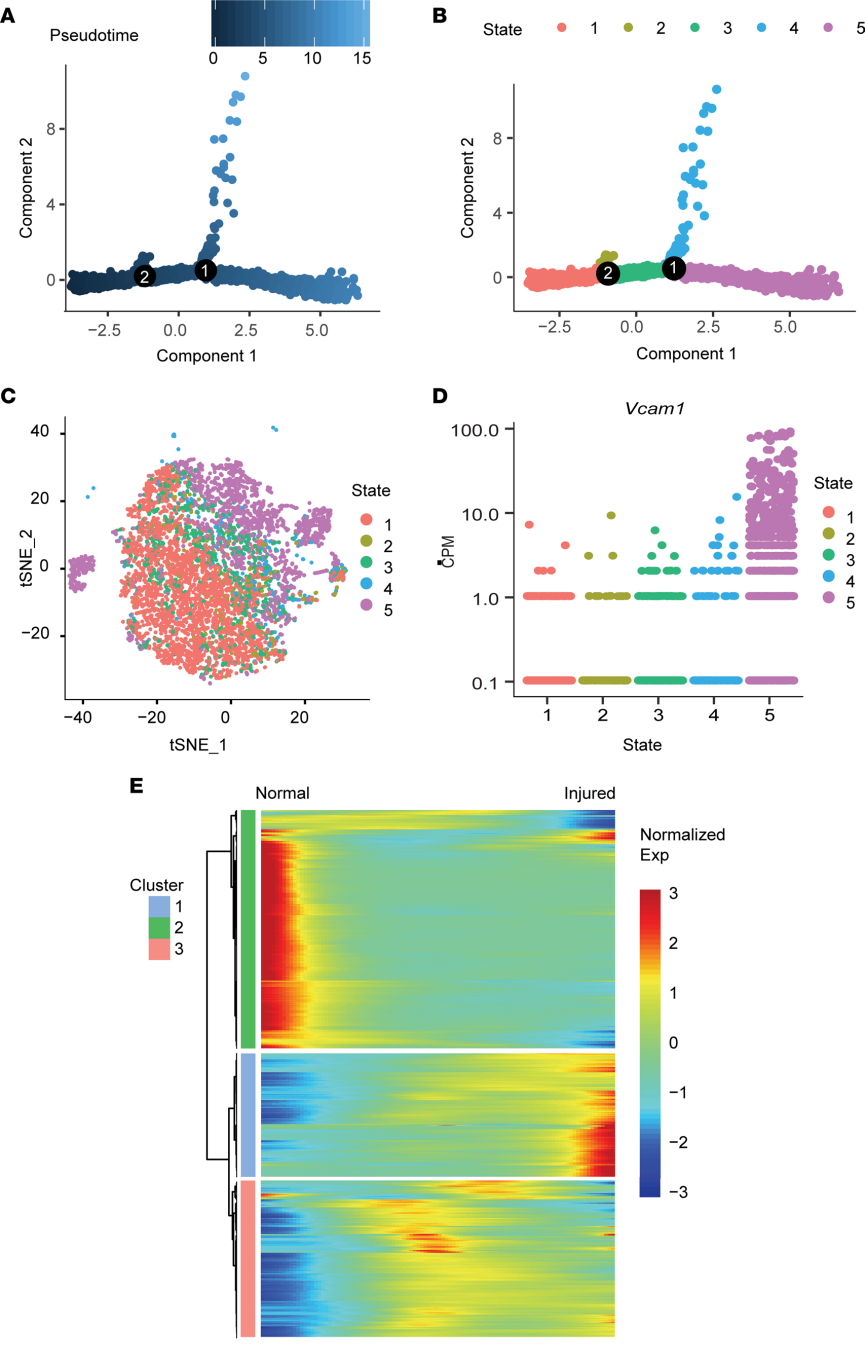
Single cell pseudotime and trajectory analysis of SMC subpopulations. (**A**) Pseudotime analysis of SMCs. (**B**) Trajectory analysis of all SMCs reconstructed by Monocle2. Cells were colored by their state identity. (**C**) Feature plot of distinct states of SMCs (as in **B**). (**D**) Expression of *Vcam1* in distinct states of SMCs. (**E**) Heatmap of the significantly modulated genes discovered in different states of trajectory analysis.

To understand the potential relationship among SMC clusters, we reconstructed single-cell trajectory of all SMCs using Monocle2 and investigated the genes whose expression levels were dynamically changed along the trajectory. We first performed Monocle2 by using standard DDRTree algorithm on SMCs from both normal and injured arteries by using 2000 highly variable genes to reconstruct a single-cell trajectory. The clustering shown on the trajectory was computed separately. To illustrate which t-distributed stochastic neighbor embedding (t-SNE) clusters are in which cell states as identified with pseudotemporal analysis, we colored cells on the trajectory by their cluster number in the t-SNE plot. Monocle2 identified a bifurcating trajectory with 2 branches (Figure 4, A and B). Clusters 0–3 were found at 1 state (state-4), and clusters 4, 6, and 8 were found at another state (state-5) (Figure 4C). The branch points and overall trajectory were determined by the differential expression of a number of genes such as the markers of SMC phenotypes. For example, at branch point 1, contractile marker genes such as Acta2, Cnn, and Taglin show lower expression in the state-5 branch, including clusters 4, 6 and 8. State-4 cells expressed genes such as Cd200, suggesting these cells may play a protective role in atherogenesis via inhibiting macrophage activation. Notably, cluster 4 and cluster 8 were found in the same state and they expressed vascular cell adhesion molecule-1 (*Vcam1*) at higher levels than the other SMCs (Figure 4, C and D). Further studies are needed to clarify whether SMCs in cluster 4 transition to cluster 8 after injury. By inferring pseudotime along the trajectory, the portion of SMCs from injured arteries increased gradually (Supplemental Figure 4). Taken together, the single-cell trajectory inferred by Monocle2 could predict the progression of a subpopulation of SMCs in normal arteries to proinflammatory SMCs.

We then sought to identify the genes that were dynamically regulated during the progression of SMCs into inflammatory phenotype by identifying the genes being variably expressed along the pseudotime. We identified 1289 highly variable genes, which could be classified into 3 clusters based on their expression pattern along the pseudotime (Figure 4E). The first cluster contained genes that were highly expressed in normal SMCs but were downregulated under injury conditions. The second cluster contained genes that were low in normal conditions and activated in injured arteries. Genes in the third cluster appeared to be transiently activated, but then repressed in injured arteries.

### A subset of SMCs after injury upregulated the expression of heat shock proteins.

We first characterized cluster 6, one of the 2 new SMC clusters. Cluster 6 displayed a higher expression of hsp 40 (*hsp40/Dnajb1*) and *Timp1* compared with the rest of the SMCs (Supplemental Figure 5A). Studies have demonstrated that hsps play an important role in the cellular adaptation to stress and the maintenance of protein stability (26) and that *Timp1* is an inhibitor of vascular matrix degradation (27). The upregulated genes encoding hsps included *hspa1b*, *hspa1a*, *hspa8*, *Dnajb1*, and *Dnaja1* (Supplemental Figure 5B), suggesting a strong stress response of these SMCs. Putative antiproliferative target gene *Errfi1* was also enriched in cluster 6 (28). Immunostaining images showed that SMCs expressed more hsp70 after injury (Supplemental Figure 5C). In addition, other genes such as *Tnfrsf12a*, *Hbegf*, and *hspb1* in the GO term “regulation of stress response” were enriched in this cluster (Supplemental Figure 6), suggesting a stress-adaptive phenotype.

### A proinflammatory subpopulation of SMCs expressed VCAM1.

**Figure 5:**
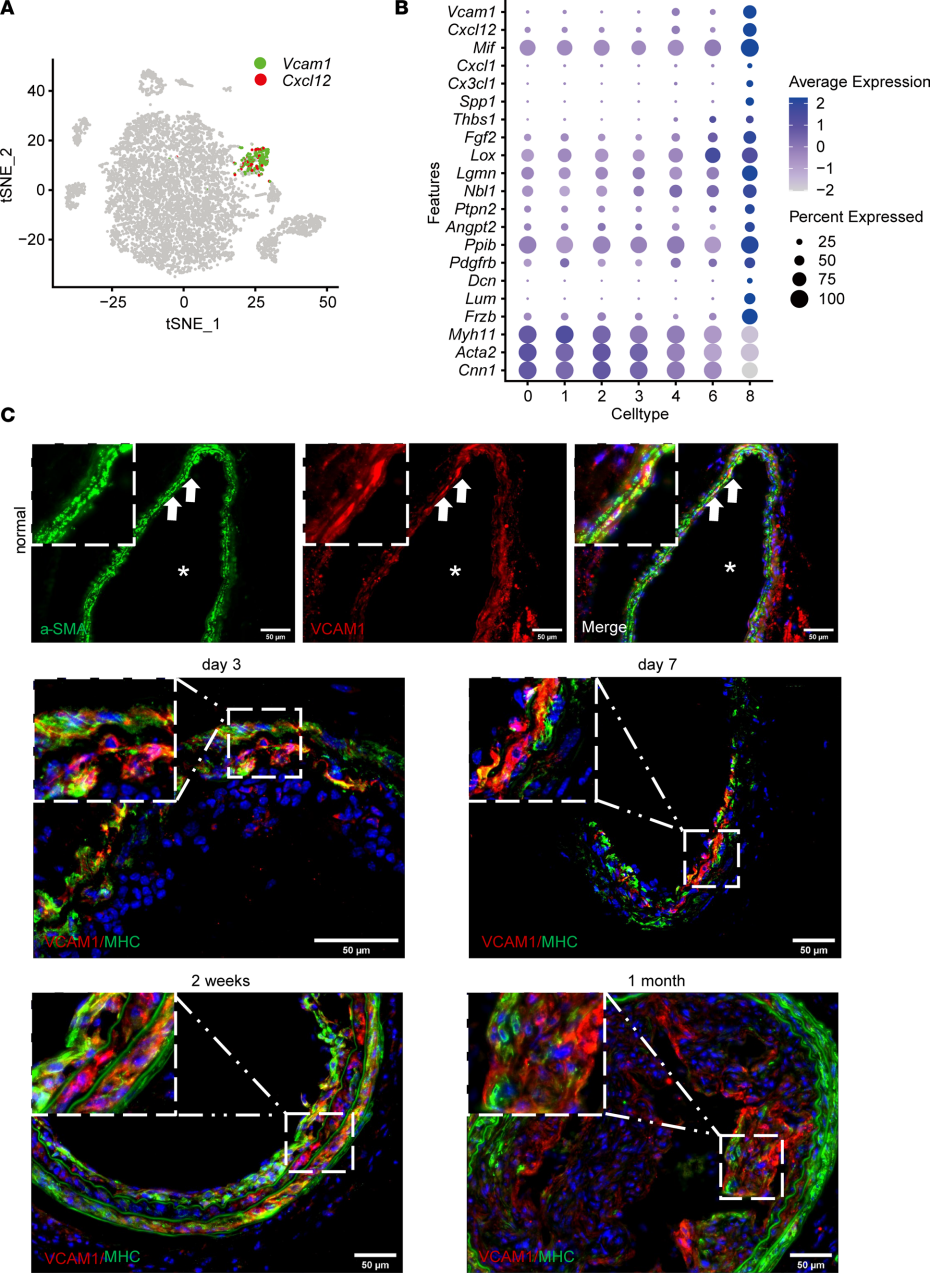
Characterization of proinflammatory SMCs in injured arteries. (**A**) Feature plot showing the expression of *Vcam1* and *Cxcl12* in cluster 8. (**B**) Differentially expressed genes in cluster 8 (*Vcam1^+^*) compared with the rest of the SMCs. (**C**) VCAM1^+^ cells were stained in normal and injured arteries at various time points (3 days to 1 month) (*n* = 5). Scale bar: 50 μm. Arrows indicate positive cells. Asterisk indicates the lumen of the artery.

Notably, cluster 8, another new SMC cluster after injury, displayed higher expression levels of inflammatory markers such as *Vcam1* (29) and chemokine (CXC motif) ligand 12 (*Cxcl12)* (30) (Figure 5A). The cluster 8 population was about 11.2% of SMCs in the injured artery at day 3 (Table 1). It is noteworthy that cluster 8 highly expressed factors involved in leukocyte chemotaxis including *Cxcl12,* *Mif* (29)*, Cxcl1*, *Cx3cl1*, *Spp1*, *Thbs1*, *Fgf2*, *Lox*, *Lgmn*, *Nbl1*, *Ptpn2*, *Angpt2*, *Ppib*, and *Pdgfrb* (Figure 5B). These results suggested that this subpopulation of SMCs had a proinflammatory role and might interact with inflammatory cells via chemotactic recruitment and cell-cell adhesion. Cluster 8 also highly expressed small leucine-rich proteoglycans such as decorin (*Dcn*) and lumican (*Lum*) (Supplemental Figure 7). In addition, genes associated with vascular calcification, such as *Spp1*, *Tnc*, and *Frzb* (Figure 5B), were highly expressed in cluster 8 (31).

We performed 2 clustering analyses independently, for normal and injured artery samples. *Vcam1*^+^ cells showed a downregulation of contractile SMC genes especially in injured arteries (Figure 5B, and Supplemental Figures 8–10). We then verified VCAM1 protein expression in SMCs using the ligation model. Compared with the low frequency of VCAM1^+^ cells detected in normal arteries, the number of VCAM1^+^ SMCs significantly increased after ligation between day 3 and 2 weeks (Figure 5C). Consistently, flow cytometry analysis also showed a relative 4.7-fold increase of VCAM1^+^VE-cadherin^–^ SMCs in the injured artery at day 7 (Supplemental Figure 11).

### Recruitment of immune cells during neointima formation.

**Figure 6:**
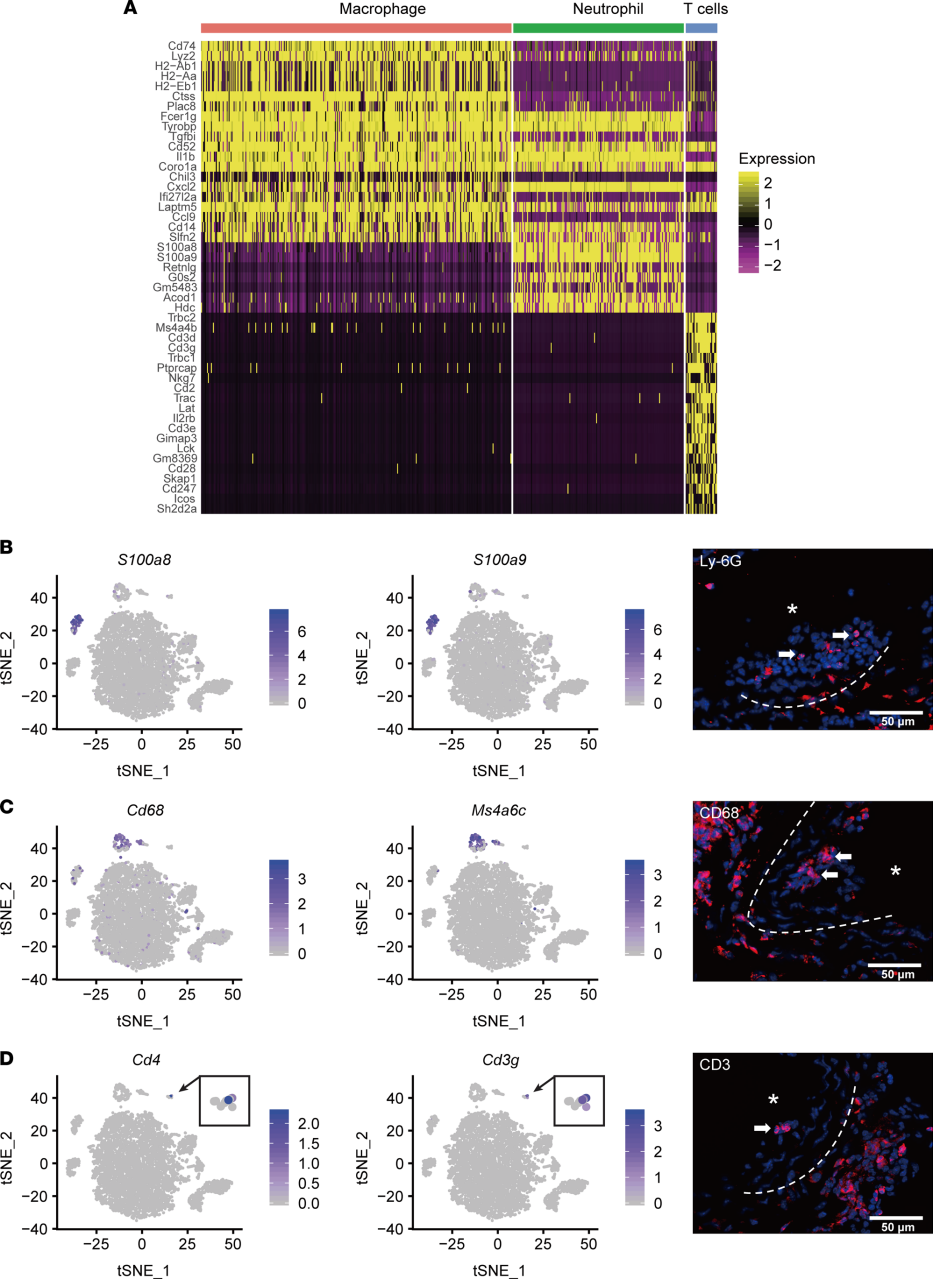
Clustering analysis of immune cells from normal and injured arteries. (**A**) Heatmap of the marker genes from 3 major immune cell clusters. (**B**) Expression of neutrophil markers *S100a8* and *S100a9* and representative immunostaining images of Ly-6G^+^ cells in injured arteries. (**C**) Expression of macrophage markers *Cd68* and *Ms4a6c* and the representative image of CD68^+^ cells in injured arteries. (**D**) Expression pattern of T cell markers *Cd4* and *Cd3g* and the representative image of CD3^+^ cells in injured arteries. (*n* = 3). Arrows indicate positive cells. Asterisk indicates the lumen of the artery. Scale bar: 50 μm.

We subclassified immune cells based on the expression of well-documented marker genes. The scRNA-Seq data showed that macrophage, neutrophil, and T cells were the major immune cells that infiltrated into the injured arteries. Highly expressed genes (*P* < 0.01 by Wilcoxon rank-sum test and log-fold enrichment > 1 in a cluster compared with other cell types) identified in different immune cell clusters were listed in Supplemental Tables 2–4, and expression patterns of these genes among immune cells clusters were visualized as a heatmap (Figure 6A). Specifically, cluster 10 cells were classified as neutrophils, which were characterized by the expression of *S100a8* and *S100a9* (Figure 6B). Cluster 7 cells were identified as macrophages, which showed high expression of *Cd68* and *Ms4a6c* (Figure 6C). Cluster 12 cells were classified as T cells, which exhibited high expression of T cell markers *Cd4* and *Cd3g* (Figure 6D).

These CD68^+^ macrophages had enriched expression of *CD74* (32), *Lyz2* (33), *Ms4a6c,* and *Gngt2* (19), suggesting that this cluster represented inflammatory macrophages (Supplemental Figure 12A). Proatherosclerotic cytokines *Il1b*, *Nlrp3,* and *Ph1da1* and chemokines *Pf4* and *C1qa* were highly expressed in cluster 7 (19, 34). The cells in cluster 7 also expressed various proinflammatory cytokines (*Tnf*, *Il1b*, and *Il66*) and chemokines (*Cxcl2*, *Cxcl10*, *Cxcl11*, *Ccl3*, *Ccl4*, *Ccl5*, *Ccl6*, and *Ccl9*). Indeed, the enriched GO term proinflammatory response further suggested cluster 7 includes proinflammatory macrophages (Supplemental Figure 12B). GO term analysis revealed that cluster 7 marker genes participated in cytokine-cytokine receptor interaction and chemokine signaling pathways. In addition, cells in cluster 7 expressed *S100a4*, also known as fibroblast-specific protein-1 (Fsp-1) (Supplemental Figure 12C), a gene expressed in inflammatory macrophages involved in tissue remodeling (35). Taken together, these results showed that the macrophages in injured arteries were proinflammatory macrophages. In addition, Cluster 7 includes a big cluster and a small cluster. The big cluster comprised proinflammatory Mo macrophages and the small cluster comprised proinflammatory M1 macrophages (Supplemental Figure 12D, and Supplemental Tables 5 and 6).

To verify the presence and distribution of immune cells in neointima, we performed immunohistological analysis of the ligated arteries. We identified Ly-6G^+^, CD68^+^, and CD3^+^ cells, respectively, in the lumen area 3 days after ligation (Figure 6, B–D), suggesting the recruitment and involvement of immune cells in the early stage of neointima formation.

### The interaction between macrophages and cluster-8 SMCs was a major intercellular communication among all cell types.

**Figure 7:**
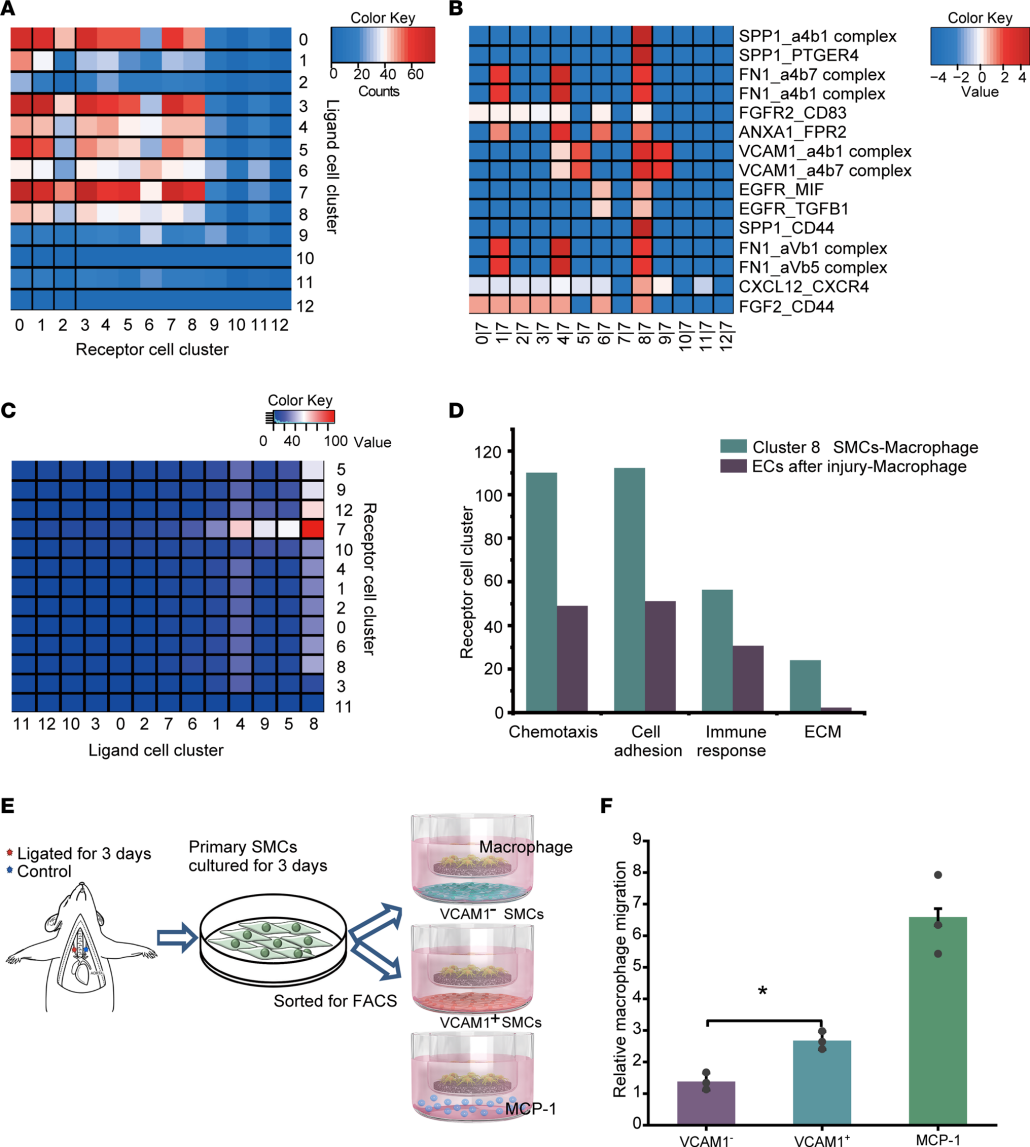
Interactions of proinflammatory SMCs with immune cells. (**A**) Heatmap showing the number of significant ligand-receptor pairs identified between each pair of cell clusters. Rows represent ligand cell clusters, and columns represent receptor cell clusters. (**B**) Heatmap showing the normalized mean expression (transcription) values for selected interacting partners between cell cluster 7 (macrophage) and all other 13 cell clusters. Red color indicates high expression values, whereas blue indicates low mean expression. The expression mean value is defined as the mean of the individual partner average expression values in the corresponding interacting pairs of cell types. (**C**) Heatmap showing the sum of mean expression values in the inflammatory pairs between cell clusters. (**D**) Fold enrichment of sum of mean expression values of different ligand-receptor pairs of ligand cells (proinflammatory SMCs and ECs after injury) and receptor cells (macrophage). (**E**) Schematic diagram of the experimental procedure for chemotactic analysis. (**F**) Monocytes were cultured in the upper chamber of the transwell, and VCAM1^+^ SMCs, VCAM1^–^ SMCs, or MCP-1 (positive control) were in the lower chamber. The recruitment of monocytes was quantified after 6 hours of coculture (*n* = 3). *P < 0.05 by 1-way ANOVA followed by Tukey’s post hoc test.

The interactions of SMCs, ECs, and immune cells were computationally predicted by evaluating the expression level of ligands and receptors. Intercellular communications between macrophages (cluster 7) and other cluster cells were the dominating interactions (Figure 7A), which indicated a critical role of the macrophage in vascular injury. Figure 7B showed the normalized mean expression (transcription) values for selected interacting partners between cell cluster 7 (macrophage) and all other 13 cell clusters. Among all clusters, the interactions of clusters 7 and 8 (proinflammatory SMCs) were most prominent. Ligand-receptor pairs that were highly expressed in cluster 8/cluster 7 interactions included *FN1*-*a4b1*, *a4b7*, *Vcam1-a4b1, a4b7, and CXCL12-CXCR4* (Permutation test *P* value < 0.05), suggesting the proinflammatory role of cluster 8 SMCs in the recruitment and adhesion of macrophages. The heatmap showed that SMCs in cluster 8 had a strong interaction with macrophages, as shown by column “8|7”. Figure 7C showed the sum of mean expression values in the inflammatory pairs of each cell cluster, which suggested that intercellular communications between cluster 8 SMCs and macrophages were the major interactions between vascular cells and inflammatory cells. Comparable enrichment of chemotaxis, cell adhesion, immune response, and ECM between the interaction of cluster 8 SMCs with macrophages and the interaction of cluster 9 ECs (activated after injury) with macrophages further indicated a major proinflammatory role of SMCs in cluster 8 (Figure 7D). Moreover, the cellular communication between ECs and macrophages involved migration, adhesion, and regulation of the inflammatory response (Supplemental Figure 13). Enriched GO terms for the communication between cluster-8 SMCs and cluster-9 ECs (after injury) showed various ligand-receptor pairs that were involved in mesenchyme development, including *Notch1-Jag1*(ligand receptor), *Jag1*-*Notch2,* and *Mdk-Ptprz1* (Supplemental Figure 14).

### Chemotactic effect of VCAM1^+^ SMCs and their distribution in diseased arteries.

To further investigate the interactions of cluster-8 SMCs with macrophages, we examined the role of VCAM1^+^ SMCs as an example. It has been shown that VCAM1^+^ SMCs mediate the adhesion of inflammatory cells (36, 37), but the chemotactic effect of VCAM1^+^ SMCs was not well understood. To directly assess the chemotactic role of VCAM1^+^ SMCs in cluster 8, VCAM1^+^ and VCAM1^–^ SMCs were sorted by FACS (Supplemental Figure 15) and cocultured with monocytes for chemotactic analysis using Transwell (Figure 7E). Figure 7F showed that VCAM1^+^ SMCs recruited more monocytes than VCAM1^–^ SMCs, consistent with the high expression level of cytokines in VCAM1^+^ SMCs.

**Figure 8:**
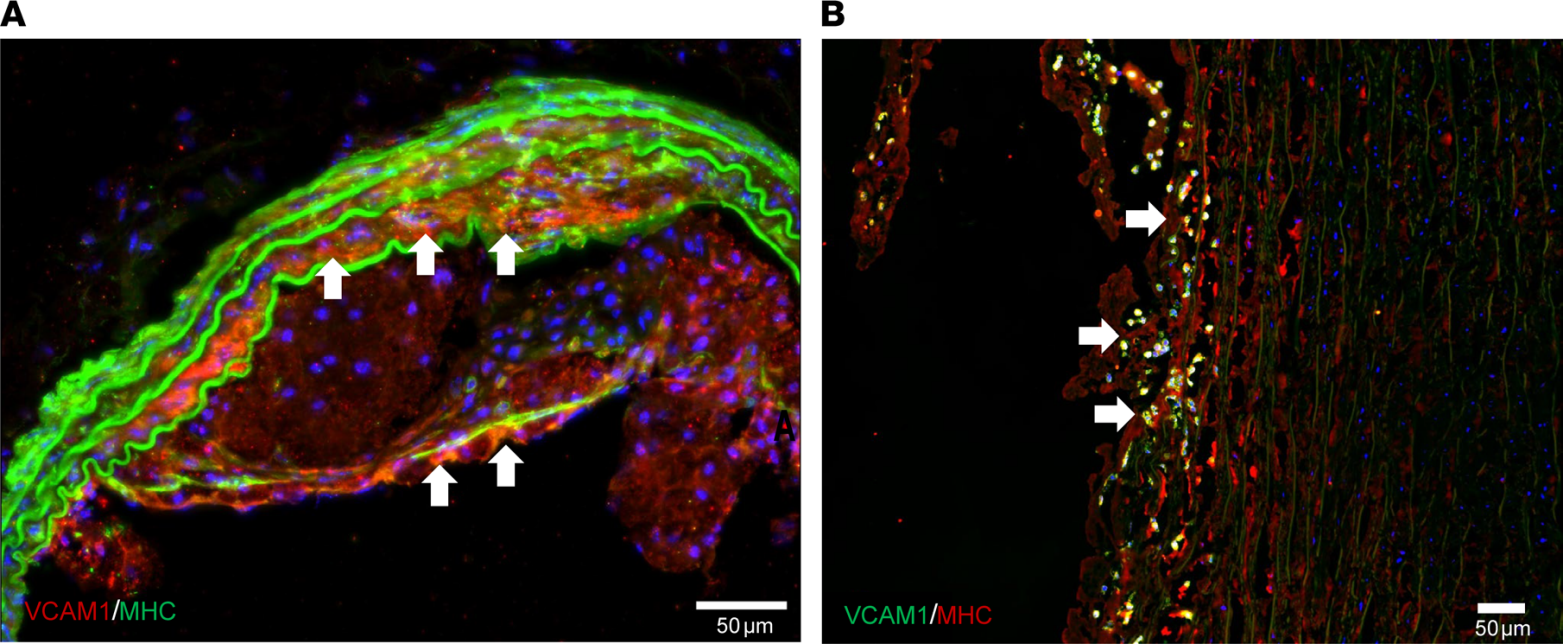
VCAM1^+^ cells in atherosclerotic lesions in mouse and human arteries. (**A**) Immunofluorescence staining showing VCAM1^+^MHC^+^ cells in atherosclerotic plaques in ApoE^–/–^ mice (*n* = 3). Arrows indicate VCAM1^+^MHC^+^ cells. (**B**) Immunofluorescence staining showing VCAM1^+^MHC^+^ cells in atherosclerotic plaques in the human aorta (*n* = 3). Arrows indicate VCAM1^+^MHC^+^ cells. Scale bar: 50 μm.

To determine whether VCAM1^+^ SMCs were involved in atherosclerosis, we examined the presence of VCAM1^+^ SMCs in ApoE^–/–^ mice fed with Western diets. Indeed, we found 40.7% VCAM1^+^ SMCs in atherosclerotic lesions in ApoE^–/–^ mice (Figure 8A). In addition, we identified VCAM1^+^ SMCs in atherosclerotic lesions in human arteries, suggesting an important role of VCAM1^+^ SMCs in vascular disease development (Figure 8B). Taken together, these data suggested that VCAM1^+^ SMCs were induced in injured arteries as a distinct proinflammatory subpopulation of SMCs.

### A subset of SMC-lineage cells expresses VCAM1^+^ in response to injury.

To directly determine whether VCAM1^+^ SMCs were derived from SMCs, we used transgenic mice to perform lineage tracing of SMCs. These animals express the tamoxifen-inducible Cre recombinase under the control of the Myh11 promoter. The activation of the Cre recombinase by tamoxifen administration causes in SMC-specific deletion of a stop cassette at multicolor reporters (Confetti) so that SMC-derived cells are labeled. The lineage-tracing mice were subjected to carotid ligation injury. After 30 days, the carotid artery was harvested, and the cross sections were stained for VCAM1 (Supplemental Figure 16). The results showed that red-fluorescent protein–positive (RFP-positive) SMCs, a major clone of SMC lineage tracing, and VCAM1^+^ SMCs were found in both media layer and neointima.

## Discussion

In this study, we unbiasedly defined the diversity, phenotypes, and transcriptomes of artery cells before and after injury by using scRNA-Seq. Overall, ECs and SMCs had distinct patterns of responses at the early stage. While ECs showed homogeneous responses to upregulate mesenchymal genes, SMC responses were limited to less than 30% of their population. Although endothelial-to-mesenchymal transition (EndMT) has been reported in neointima formation and atherosclerosis (38–41), EndMT efficiency is generally low and the significance is controversial. To our knowledge, this finding is the first demonstration of a global EC response at the single-cell level. This homogeneous phenotypic change of ECs may be context-dependent and depends on the type of vascular injuries, which may play an important role in neointima formation in this complete ligation model (21).

SMC responses were limited to a fraction of SMCs, which only generated 2 new clusters of SMCs after injury: proinflammatory SMCs and stress-responsive SMCs. The gene expression profiles for these SMC subpopulations revealed their distinct functional capabilities, and immunohistological analysis confirmed the presence of these 2 subpopulations in injured arteries. A subset of SMCs after injury upregulated the expression of hsps. Cluster 6 (13%), a new SMC cluster following injury, displayed a higher expression of hsps. Hsps play an important role in the cellular adaptation to stress, a requisite for cell survival (26). These hsps may act as molecular chaperones, playing essential roles in mediating protein folding, assembly, transport, and degradation (42, 43). It has been demonstrated that hsps could be synthesized following cell isolation from the tissues and the addition of actinomycin D immediately after cell isolation could block the further synthesis of these proteins (44). In our study, this hsp induction is unlikely to be the artifact of cell isolation because only a subpopulation of cells had higher expression. In addition, the percentage of SMCs with a high level of hsp expression in normal and injured arteries was very different (1.2% and 13%, respectively). Diverse stresses, including heavy metals, aa analogues, inflammation, and oxidative/ischemic stress, could induce the expression of hsp genes (45). Stress or hsps have been shown to be augmented in arterial SMCs during atherosclerosis (46). Induction of hsps could be important in protecting SMCs from injury and stress-responsive SMCs could be targeted to protect from vascular injury.

The proinflammatory SMCs were highly enriched for VCAM1 and cytokine expression. VCAM1 is generally considered as a cell-adhesion molecule specifically expressed in activated ECs to mediate the adhesion of inflammatory cells (47). Although there are reports suggesting that SMCs may express VCAM1 (29, 48), the role of these SMCs in the chemotactic interactions with inflammatory cells is not well understood. Our ligand-receptor pair analysis and chemotactic experiments demonstrated, for what we believe to be the first time, the role of VCAM1^+^ SMCs in the chemotactic recruitment of macrophages. A recent study showed that SMC-derived intermediate cell state accounted for the largest proportion among SMC lineages in advanced atherosclerosis. To address molecular and functional characteristics of this cell state, they analyzed differentially expressed genes between the SMC-derived intermediate cell state and SMCs and found the *Ly6a*, *Vcam1*, and *Ly6c1*, known markers for stem cells (49), EC (50), and monocyte/macrophage differentiation (51), respectively, were enriched within SMC-derived intermediate cells (15). It is interesting that these VCAM1^+^ SMCs also expressed stem cell and monocyte/macrophage markers. Whether these cells are related to proinflammatory VCAM1^+^ SMCs identified in our study remains to be determined. Our findings also suggest that VCAM1^+^ SMCs could be induced by other vascular injuries such as ligation, in addition to atherosclerosis. Furthermore, we used lineage tracing to directly demonstrate that SMCs could become VCAM-1–positive.

To better understand the heterogeneity of phenotypes that SMC can acquire within atherosclerotic lesions, another group performed scRNA-Seq analyses on late-stage lesions microdissected from advanced brachiocephalic artery lesions. Interestingly, most of the clusters containing lineage-traced SMCs also expressed markers such as *Vcam1*, *Dcn/Lum*, and *Spp1*. This study also found VCAM1^+^ SMCs in the late-stage atherosclerotic lesion, which may be related to the proinflammatory SMCs following the ligation injury (52).

Proinflammatory SMCs also showed an increased expression of endochondral and bone-associated genes, including *spp1*, *Tnc*, and *Frzb*, implying a possible role for proinflammatory SMCs in vascular calcification. This is consistent with the previous findings that the procalcificatory phenotype of SMCs and inflammation contributed to vascular calcification (31, 53). It is of interest to note that SMCs may have a generalized response to various vascular stresses such as hyperlipidemic or mechanical injury, which has huge implications for the possible use of SMC to target vascular disease.

A recent study (20) found that SMCs underwent phenotypic modulation along a continuous trajectory from a contractile SMC toward a fibroblast-like cell, which displayed a decreased expression of SMC genes and upregulation of small leucine-rich proteoglycans such as *lum*, *Dcn*, and biglycan (*Bgn*). Consistent with this data, proinflammatory SMCs in cluster 8 also highly expressed *lum* and *Dcn*. Our findings suggest only a subpopulation of proinflammatory SMCs had this phenotype change. Whether these cells are related to a specific fibroblast-like “fibromyocyte” remains to be determined.

Immune cells that are recruited to the subintimal space contribute to neointima formation (2, 54, 55). In our study, we identified 3 types of immune cells infiltrated into blood vessels after injury, including neutrophil, macrophage, and T cells. In addition, we found that most of the macrophages had inflammatory phenotypes. Interestingly, we also defined a subpopulation of macrophages that participated in the early neointima formation as Fsp-1^+^ macrophages. It has been demonstrated that Fsp-1 is a marker of a specific subpopulation of inflammatory macrophages in liver injury (35), and it could colocalize with CD68 in the injured heart (56). Studies have demonstrated that Fsp-1 mediates macrophage recruitment in vivo (57). A recent study (34) used scRNA-Seq to define aortic macrophage heterogeneity in atherosclerosis and found that inflammatory macrophages enrichment in *Il1b* were detectable in atherosclerotic aortas. Thus, inhibition of Fsp-1 might be a potential therapeutic strategy for neointima formation.

In summary, this study unravels the homogeneous EC responses and the limited and clustered responses of SMCs following ligation injury, which underscores the importance of understanding the mechanisms of vascular remodeling at the single-cell level. These findings may help us develop a new approach to target specific subpopulations of SMCs for vascular disease therapy.

## Methods

### Mice and tissue dissection.

C57BL/6 mice were purchased from The Jackson Laboratory. To avoid data variation incurred by sex difference (58), only 8-week-old female mice were selected for the study. Anesthesia was induced with 3% isoflurane followed by s.c. injection of 0.1 mg/kg buprenorphine and 5 mg/kg meloxicam for analgesia. Maintenance anesthesia was with 1.5%–2% isoflurane delivered by mask. Toe pinch was performed to confirm the anesthetic depth. The left carotid artery was completely ligated with 6-0 silk sutures just proximal to the carotid bifurcation to disrupt blood flow and ligation was upheld for the entire time course (21, 59–61). Animals were sacrificed 3 days after surgery. This time point was chosen to identify the early changes in medial SMCs that lead to their dedifferentiation and migration during the vascular remodeling before the development of any significant neointima (62, 63). Before dissection, perfusion was performed with sterile saline via the left ventricle. The animals were euthanized by CO_2_ inhalation until they ceased breathing completely and then followed by cervical dislocation. Carotid arteries above the ligation site were harvested and adipose and connective tissues were removed. Carotid arteries were incubated in collagenase Type I (2 mg/mL; Life Technologies) for 10 minutes and then adventitia was peeled off. Isolated tissue was pooled from 5 mice in each group (normal and injured) and further digested for 1–2 hours to obtain a single-cell suspension. Isolated cells were centrifuged at 300*g* for 3 minutes at 4°C and resuspended in 3% BSA in PBS. For 4 months, 8-week-old ApoE^–/–^ mice were fed with Western diets (TD.88137, Envigo Teklad) and used for experiments to examine the atherosclerotic lesions.

### scRNA library construction and sequencing.

scRNA-Seq was performed on dissociated cells using 10X Genomics Chromium Single Cell 3’ v2 kit, following the standard protocol from the manufacturer. The libraries generated and were sequenced on NextSeq500 with the high output kit setting as 26 bp Read1 and 58 bp Read2.

### Sequencing data processing and scRNA-Seq data analysis.

Raw and processed sequencing data are available in the Gene Expression Omnibus (GEO) repository under accession number PRJNA628067. The sequencing data were processed using CellRanger v2.0 pipeline, with mm10 as the mouse reference genome. The output cell expression matrix was then introduced into R package Seurat V3 for subsequent statistical analysis, including quality control, data normalization, clustering, and visualization. Specifically, cells with less than 200 genes detected or more than 4000 genes detected were removed to filter low-quality cells and potential cell doublets. Cells with more than 10% of reads originated from mitochondrial genomes were also removed. Data were then normalized using NormalizeData function with parameter scale.factor = 1 × 10^4^. Normalized data were then scaled to remove sequencing depth bias and influence of mitochondrial reads, using ScaleData function in Seurat. principal component analysis (PCA) and t-SNE dimensional reduction were performed using 1970 highly variable genes, and unbiased clustering was performed using the FindClusters function with resolution = 0.6. Marker genes for each cluster were identified using FindAllMarkers function with default parameters, with adjusted *P* value less than 0.05 and log_2_ fold change greater than 1.

### GO analysis.

GO analysis of gene sets is performed by GeneOntology website tool (http://geneontology.org/). Briefly, a hypergeometric test was performed using all genes in the mouse (Mus musculus) genome as a background list. A GO term is significant if its *P* value is less than 0.05 after multiple-test corrections by the Benjamini-Hochberg method.

### Reclustering and dimensional reduction of particular types of cells.

Reclustering and dimensional reduction of SMCs and macrophage cells were performed following the same procedure as we used for clustering all cells. Specifically, SMCs or macrophage cells were selected, this subset of data were renormalized and scaled, and highly variable genes for each cell type were identified. Dimensional reduction and clustering were then performed on SMCs or macrophage cells separately using highly variable gene sets for each cell type, respectively.

### Pseudotime trajectory analysis.

Pseudotime trajectory analysis was performed by the Monocle2 software package. Briefly, a gene count matrix with all 15,681 expressed genes (genes with at least 1 read in at least 10% of cells) among 6225 cells. The cell trajectory reconstruction and pseudotime analysis were performed on this gene count matrix using Monocle2 with default parameters. Genes with the mean expression level between 0.0125–3 and variance higher than 0.5 were used to reconstruct the trajectory of the subpopulations.

### Ligand-receptor cellular communication analysis.

Ligand-receptor cellular communication analysis was performed using Python software package CellPhoneDB v2 in “cellphonedb method statistical_analysis” mode with default parameters. The output of CellPhoneDB was then visualized using customized R scripts.

### Primary SMCs and monocyte culture, cell sorting, and Transwell assay.

The left carotid artery of C57BL/6 mice was completely ligated with 6-0 silk sutures just below the bifurcation point. Animals were sacrificed 3 days after surgery (*n* = 3). After sacrificing the mice, the carotid arteries of the mice were isolated. Adventitia was peeled off and ECs were removed using a cotton bud. The harvested arteries were cut into pieces and then incubated with collagenase/elastase solution for 4–6 hours in 37°C. Primary cells were cultured for 3 days in a 37°C incubator with 5% CO_2_. VCAM1^+^ SMCs and VCAM1^–^ SMCs were sorted using a VCAM1 antibody (catalog ab223982, Abcam) and a FACS system (Aria SORP, BD Biosciences). Bone marrow monocytes were extracted from the femur and upper bones of 8-week-old female mice. Macrophages were cultured with RPMI medium (Gibco), 1% penicillin-streptomycin (Gibco), and 10% heat-inactivated supplemented FBS. Transwell (Corning) tests were performed by adding monocytes to the upper chamber and VCAM1^+^ SMCs or VCAM1^–^ SMCs to the lower chamber (Figure 7E). The positive control group had 20 ng/mL of MCP-1 protein in the lower chamber. After 6 hours, the cells on the lower surface of the insert were fixed and stained with DAPI. Cells were imaged with a fluorescence microscope and quantified. Statistical analysis was performed using 1-way ANOVA, followed by Tukey’s multiple comparison test (*n* = 3). The difference was considered statistically significant when *P* was less than 0.05.

### Cell sorting.

The left carotid artery of C57BL/6 mice was completely ligated with 6-0 silk sutures just below the bifurcation point (*n* = 3). To obtain enough VCAM1^+^ SMCs for FACS sorting, the arteries above the ligation site were harvested on day 7 and digested to isolate the cells. VCAM1 antibody (catalog ab223982, Abcam) and VE-cadherin antibody (catalog 562242, BD Biosciences) were used to stain the cells, and a FACS system (Aria SORP, BD Biosciences) was used to sort VCAM1^+^VE-cadherin^–^ SMCs and VCAM1^–^VE-cadherin^–^ SMCs from normal and ligated arteries. The sorted cell number was counted by the FACS system in each group.

### Generation of transgenic mice.

MYH11-cre (catalog 007742) and R26R-Brainbow (catalog 017492) mice were purchased from The Jackson Laboratory. All the male MYH11-cre mice were crossed with female R26R-Brainbow mice to generate MYH11-cre/R26R-Brainbow mice. The 2-month-old male transgenic mice were used for experiments. Tamoxifen was given to the mice through i.p. injection for 5 consecutive days, and the ligation injury was performed 1 week after tamoxifen induction. The left carotid artery of MYH11-cre/R26R-Brainbow mice was completely ligated with 6-0 silk sutures just below the bifurcation point (*n* = 3). After 1 month, the animals were euthanized, and carotid arteries were harvested and fixed in 4% paraformaldehyde for histological analysis.

### Histological analysis and immunostaining.

Carotid arteries were ligated and harvested at different time points and fixed in 4% paraformaldehyde at 4°C for 4 hours. The tissues were then cryoprotected by 15% and 30% sucrose in PBS at 4°C overnight. The samples were then embedded in optimum cutting temperature and stored at –80°C. The frozen samples were cryosectioned in 10 μm thick slices. For immunostaining, frozen sections were permeabilized in 0.5% Triton X-100 solution for 10 minutes and then blocked with 5% donkey serum for 1 hour. The samples were incubated with primary antibodies overnight at 4°C. The primary antibodies used in this study were: Ly-6G (catalog sc-103603, Santa Cruz Biotechnology), CD68 (catalog ab125157, Abcam), CD3 (catalog ab5690, Abcam), VE-cadherin (catalog sc-6458, Santa Cruz Biotechnology), SM22 (catalog ab14106, Abcam), hsp70 (catalog ab7985, Abcam), myosin heavy chain (MHC) (catalog BT-562, Biomedical Technologies), α-SMA (catalog ab575, Abcam), and VCAM1 (catalog sc-1504, Santa Cruz Biotechnology). The slides were washed with PBS 3 times and incubated with secondary antibodies at room temperature for 1 hour. Nuclei were stained with DAPI. After multiple washes with PBS, coverslips were mounted and fluorescence images were observed by Zeiss fluorescence microscope (LSM710, Zeiss).

The cryosections of ApoE^–/–^ mice arteries were costained with MHC and VCAM1. Fluorescence images were taken by Zeiss fluorescence microscope (LSM710, Zeiss). The percentage of VCAM1^+^ SMCs in atherosclerotic lesions was investigated by image analysis with ImageJ (NIH) according to the previous protocol (64).

Human aortas were obtained from the National Disease Research Interchange and the Department of Surgery, University of California, Davis. The cryosections of the human aorta were fixed with paraformaldehyde, rinsed with PBS, and blocked in 10% BSA for 1 hour. Cryosections were then incubated with MHC antibody (catalog MA5-11971, Invitrogen) and VCAM1 antibody (catalog ab223982, Abcam) for 16–18 hours, followed by staining with secondary antibodies. The samples were rinsed and counterstained by DAPI. The stained tissues were imaged using a fluorescence microscope (BX51, Olympus).

### Data availability.

Supporting information is available from the Wiley Online Library Raw, and processed sequencing data are available in the Gene Expression Omnibus (GEO) repository under accession number PRJNA628067 (https://www.ncbi.nlm.nih.gov/bioproject/PRJNA628067).

### Statistics.

All the data are shown as the means ± SD. Statistical testing by 1-way ANOVA followed by Tukey’s test was used to analyze the ANOVA of multiple groups. A value of *P* < 0.05 was considered statistically significant.

### Study approval.

All animal experiments were carried out according to the *Guide for the Care and Use of Laboratory Animals* (National Academies Press, 2011) and were approved by the Institutional Animal Care and Use Committee at UCLA.

## Author contributions

XD performed scRNA-Seq experiments, analyzed the scRNA-Seq data, performed and analyzed the IHC experiments, made the figures, and wrote the manuscript. QA, XT, and JW performed bioinformatics analysis and assisted with manuscript writing. WZ, YS, and CCC assisted with IHC experiments. GZ assisted with the design of the scRNA-Seq experiments and performed scRNA-Seq capture and library preparation for all samples. TH, GF, YF, and SL designed the experiments and interpreted the data. SL helped write and revise the paper. All authors discussed the results and reviewed the manuscript.

## Supplementary Material

Supplemental data

## Figures and Tables

**Table 1:**
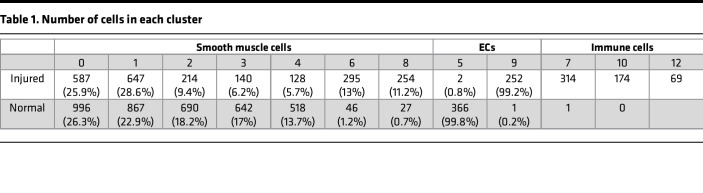
Number of cells in each cluster

## Version 1 10/24/2022

Electronic publication
