# Supplementary material for: Distinct patterns of responses in endothelial cells and smooth muscle cells following vascular injury
Source: JCI Insight. 2022 Oct 24;7(20):e153769. doi: 10.1172/jci.insight.153769 (PMC9714785; doi:10.1172/jci.insight.153769)
Supplement: Supplemental data [file jciinsight-7-153769-s196.pdf]

Supplementary materials

Supplementary Figure 1 – 16

Table S1: List of highly expressed genes identified in smooth muscle cell (SMC) clusters.

Table S2: List of highly expressed genes identified in cluster 7 (avg_logFC > 1)

Table S3: List of highly expressed genes identified in cluster 10 (avg_logFC > 1)

Table S4: List of highly expressed genes identified in cluster 12 (avg_logFC > 1)

Table S5: List of highly expressed genes identified in the big macrophage cluster (avg_logFC < -1)

Table S6: List of highly expressed genes identified in the small macrophage cluster (avg_logFC > 1)

a

Parameter	injured	normal
Estimated Number of cell	3,230	4,158
Mean Reads per Cell	64,014	51,112
Median Genes per Cell	2,375	2,703
Valid Barcodes	98.20%	98.20%
Sequencing Saturation	82.60%	76.00%
Q30 Bases in Barcode	97.50%	97.40%
Q30 Bases in RNA Read	87.90%	88.40%
Q30 Bases in Sample Index	97.10%	97.10%
Q30 Bases in UMI	96.90%	96.90%
Reads Mapped to Genome	89.40%	89.60%
Reads Mapped Confidently to Genome	82.30%	83.70%
Reads Mapped Confidently to Intergenic Region	2.10%	2.00%
Reads Mapped Confidently to Intronic Regions	3.70%	3.40%
Reads Mapped Confidently to Exonic Regions	76.60%	78.30%
Reads Mapped Confidently to Transcriptome	74.60%	76.30%
Reads Mapped Antisense to Gene	0.80%	0.80%
Estimated Number of Cells	3,605	4,756
Fraction Reads in Cells	91.60%	95.20%
Mean Reads per Cell	64,014	51,112
Median Genes per Cell	2,375	2,703
Total Genes Detected	17,434	16,953
Median UMI Counts per Cell	7,416	8,612

b

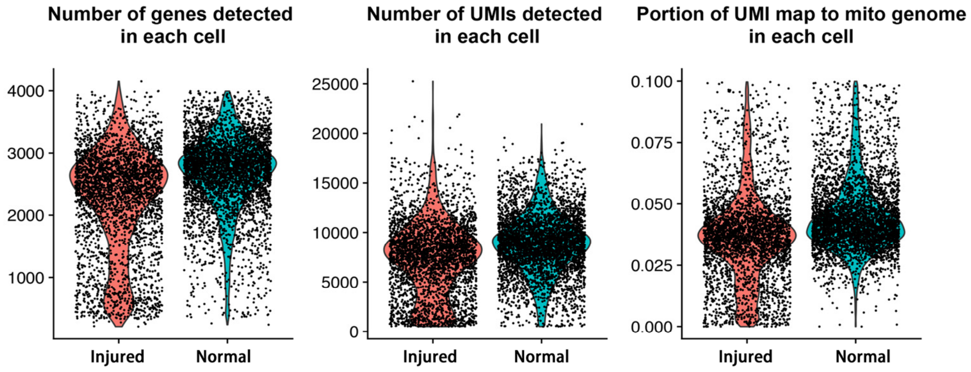

Figure S1. a An overview of sequencing data quality metrics for cells from normal and injured arteries. **b** Violin plots of basic features of sequencing data.

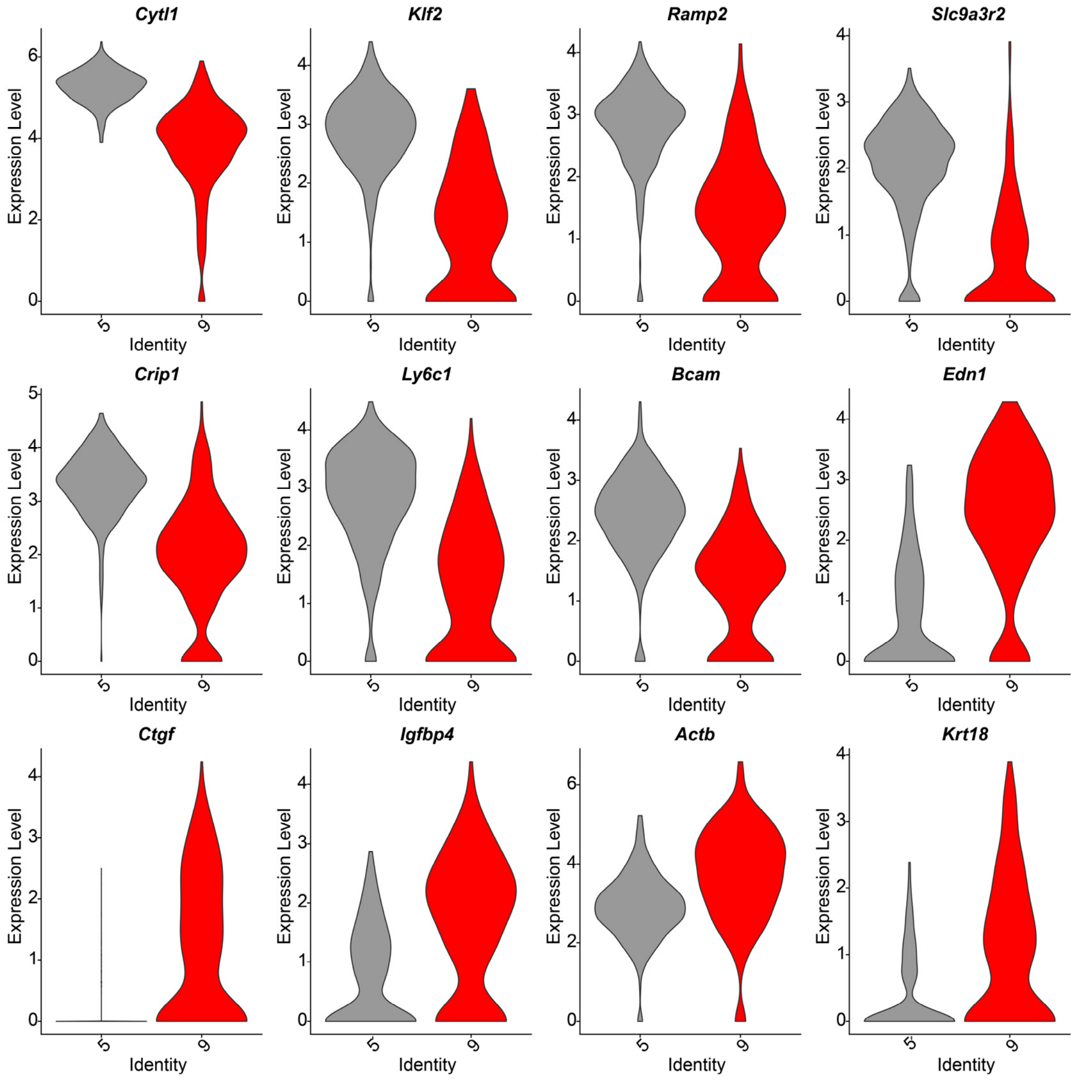

Figure S2. Violin plots of selected markers that were differentially expressed between normal and injured endothelial cells. (Cluster 5: normal, Cluster 9: Injured)

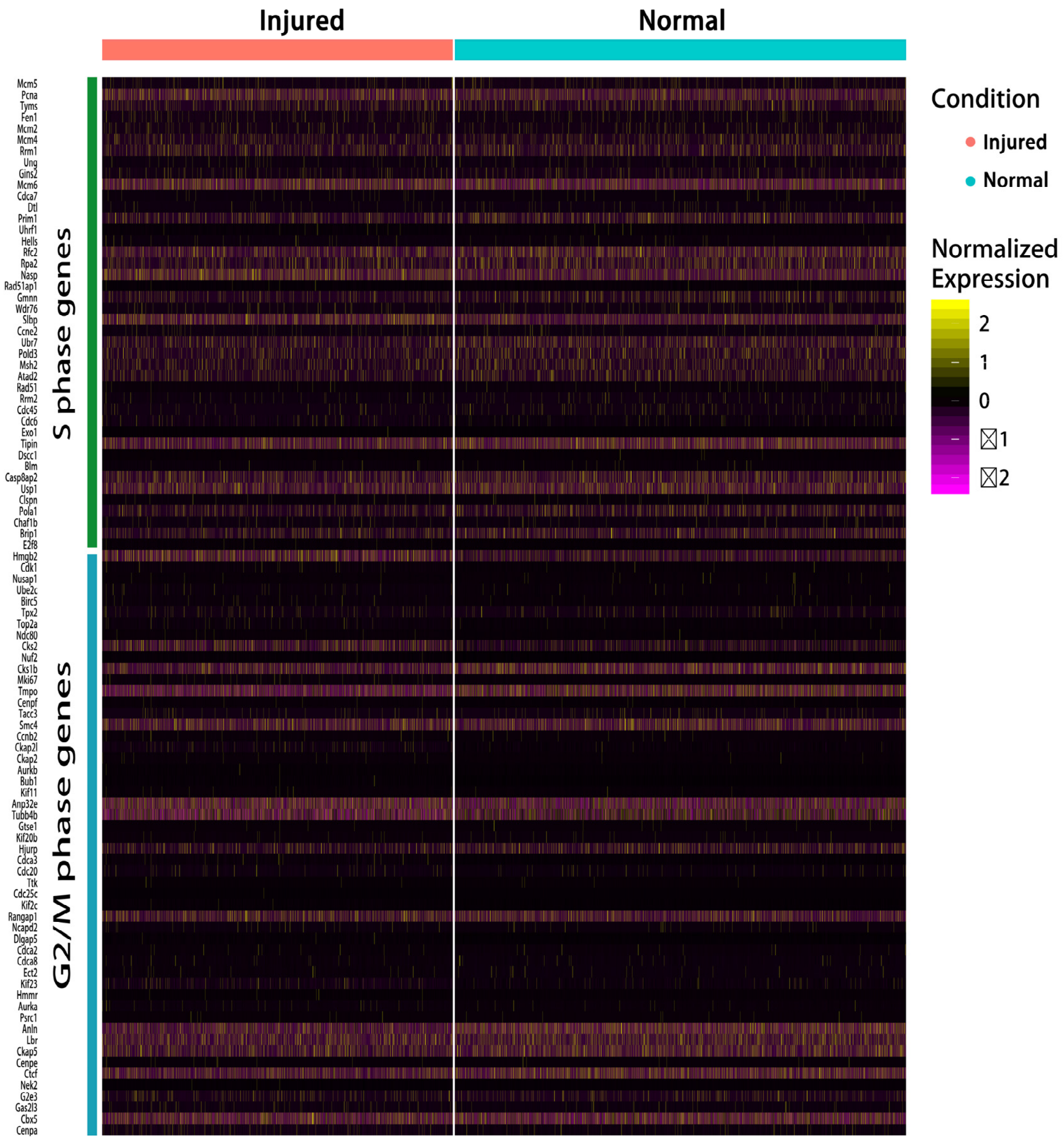

Figure S3. Heatmap of S phase genes and G2/M phase genes in normal and injured SMCs.

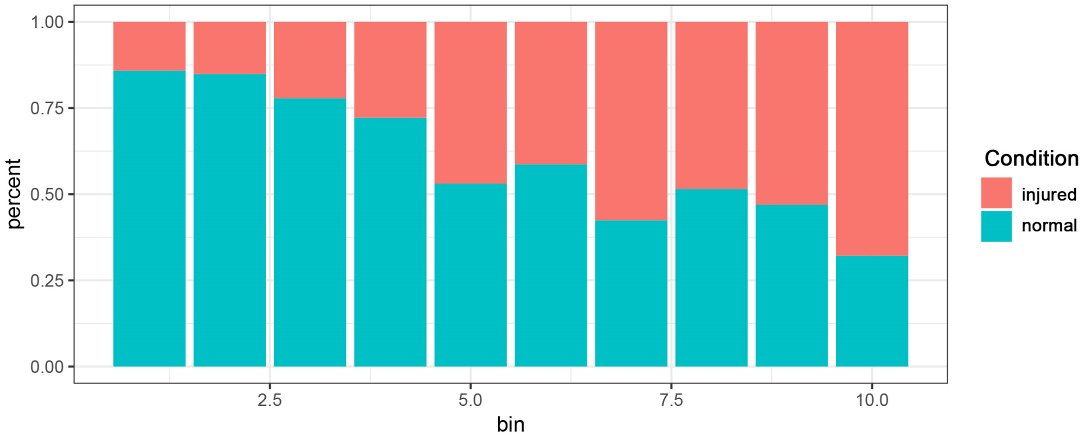

Figure S4. Stacked barplot showing how the ratio between SMCs from normal or injured conditions changes along the pseudotime of cell trajectory. Normal cells are enriched on one end of the trajectory while the injured cells are enriched in the other end. These results showed that the single cell trajectory inferred by Monocle2 represents the transition of SMCs upon injury at transcriptome level.

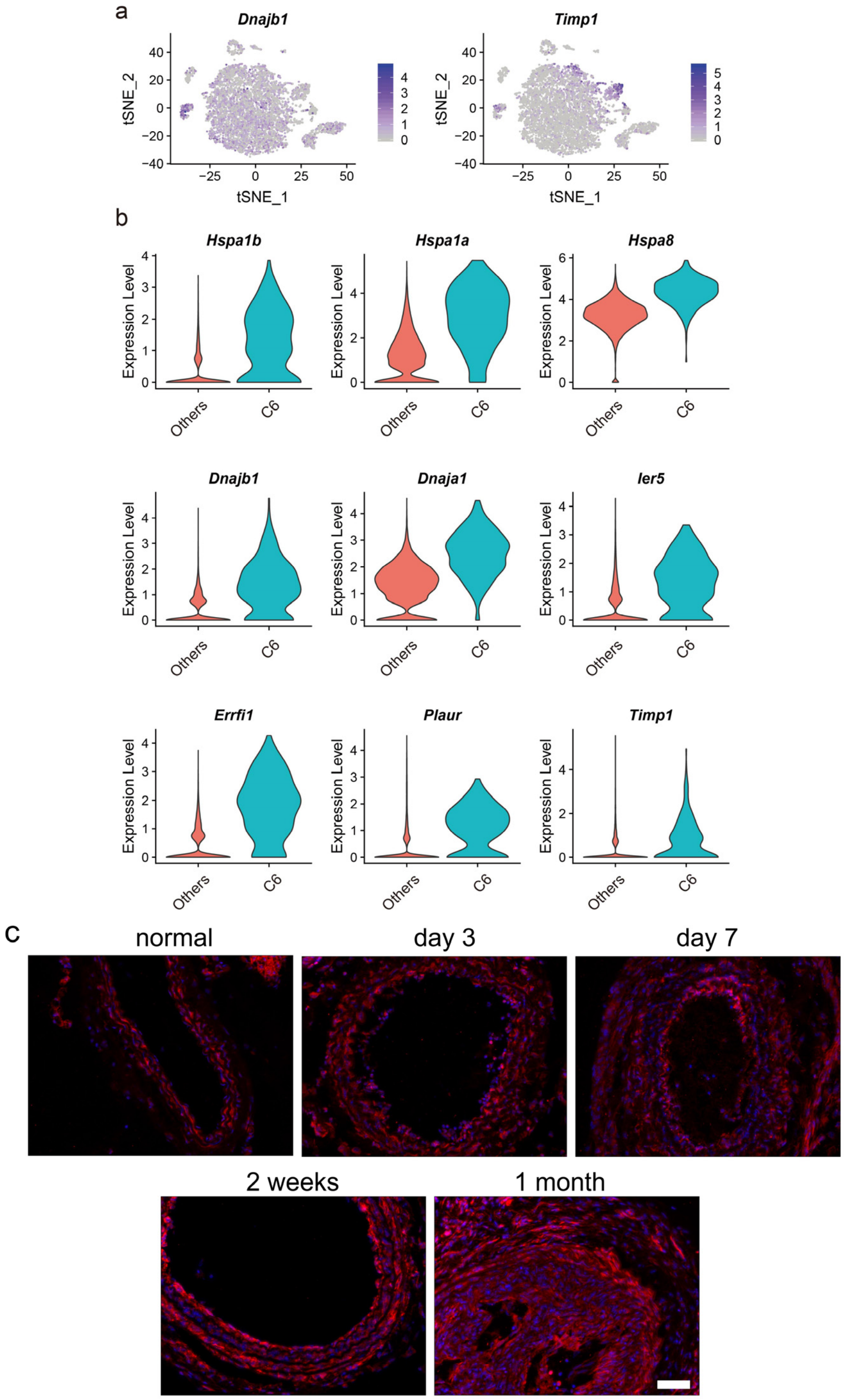

Figure S5. A subset of SMCs after injury highly expressed Hsps. **a** Expression of SMCs markers *Dnajb1* and *Timp1* in normal and injured arteries. **b** Violin plots showing the expression level of significant differentially expressed genes in cluster 6 compared with the rest of the SMCs. **c** Immunofluorescence staining of Hsp70 was performed in no injury controls and at different time points after ligation. Immunostaining images showed that SMCs expressed more Hsp70 after injury. Scale bar: 50 μm.

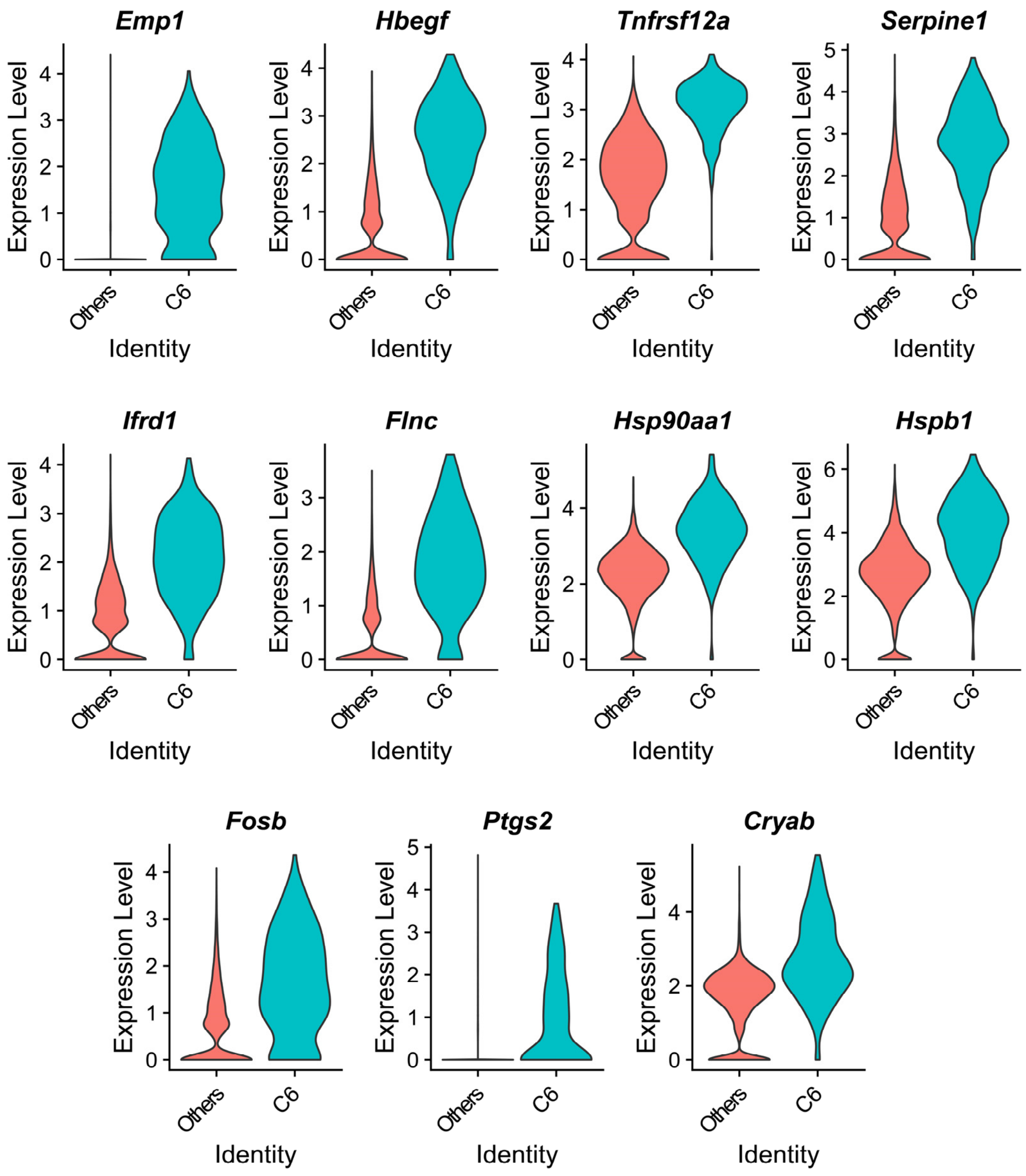

Figure S6. Violin plots showing the expression level of significant differentially expressed genes in cluster 6 compared with the rest of the SMCs.

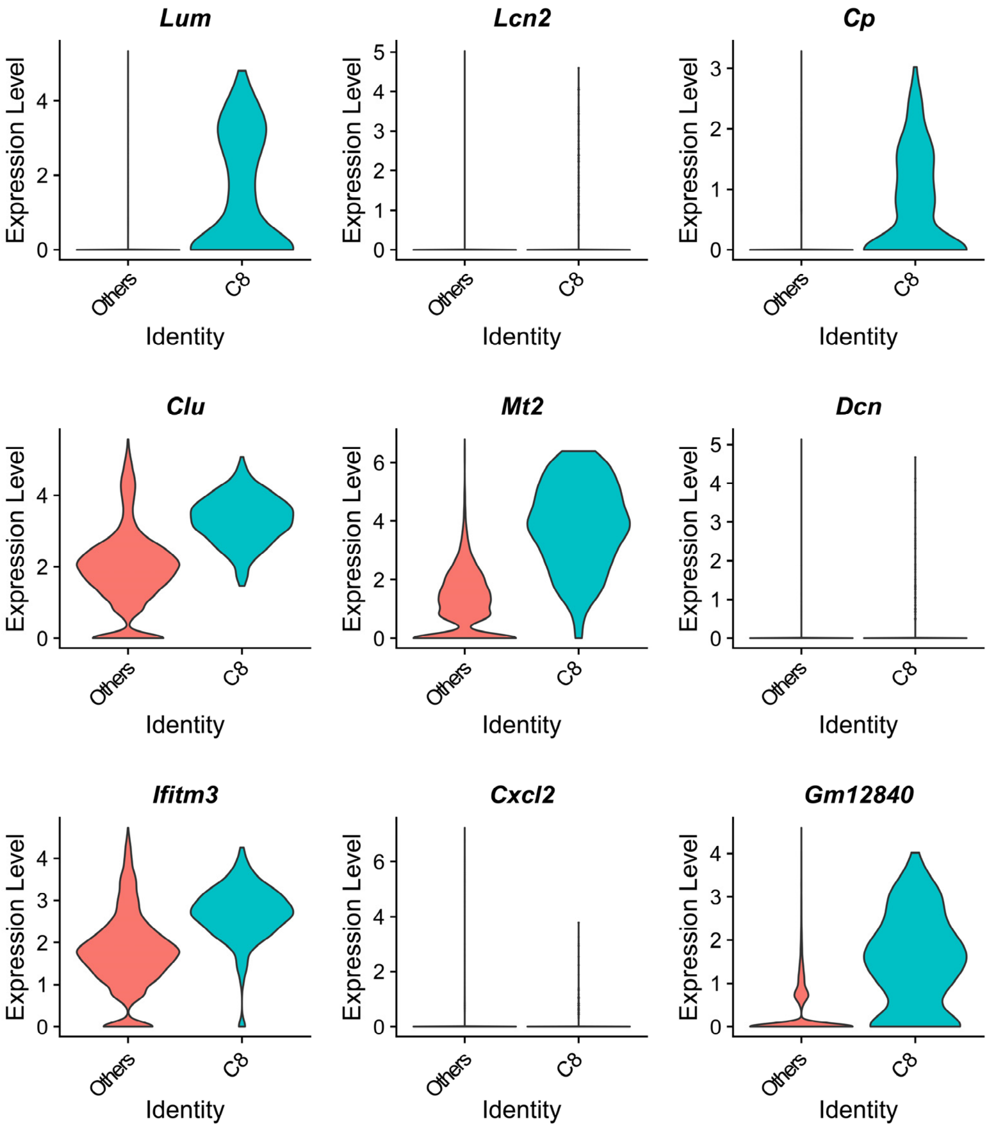

Figure S7. Violin plots of significant differentially expressed genes in cluster 8 compared with the rest of the SMCs.

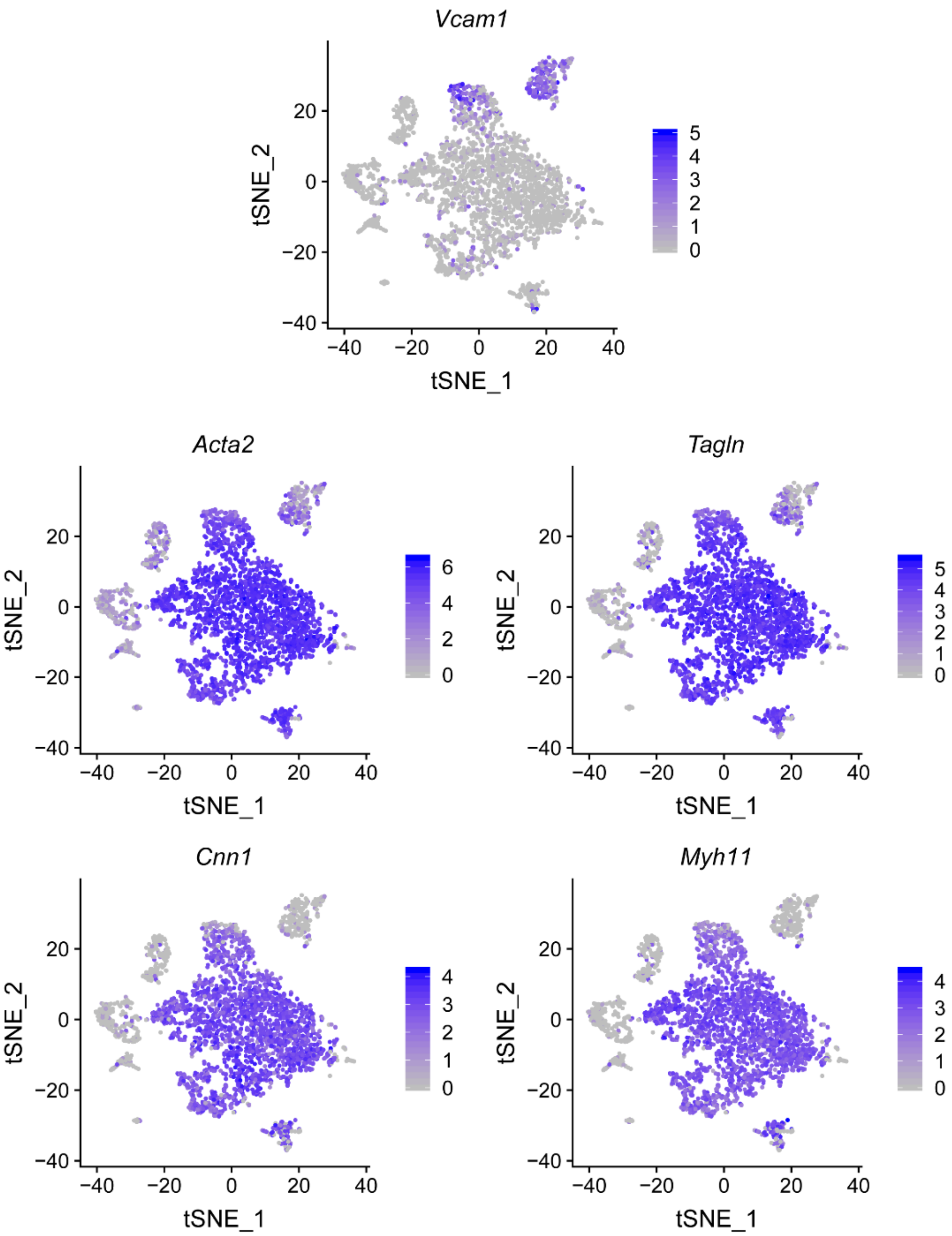

Figure S8. Feature plot of *Vcam1* and SMCs markers *Acta2*, *Tagln*, *Cnn1*, and *Myh11* in injured arteries.

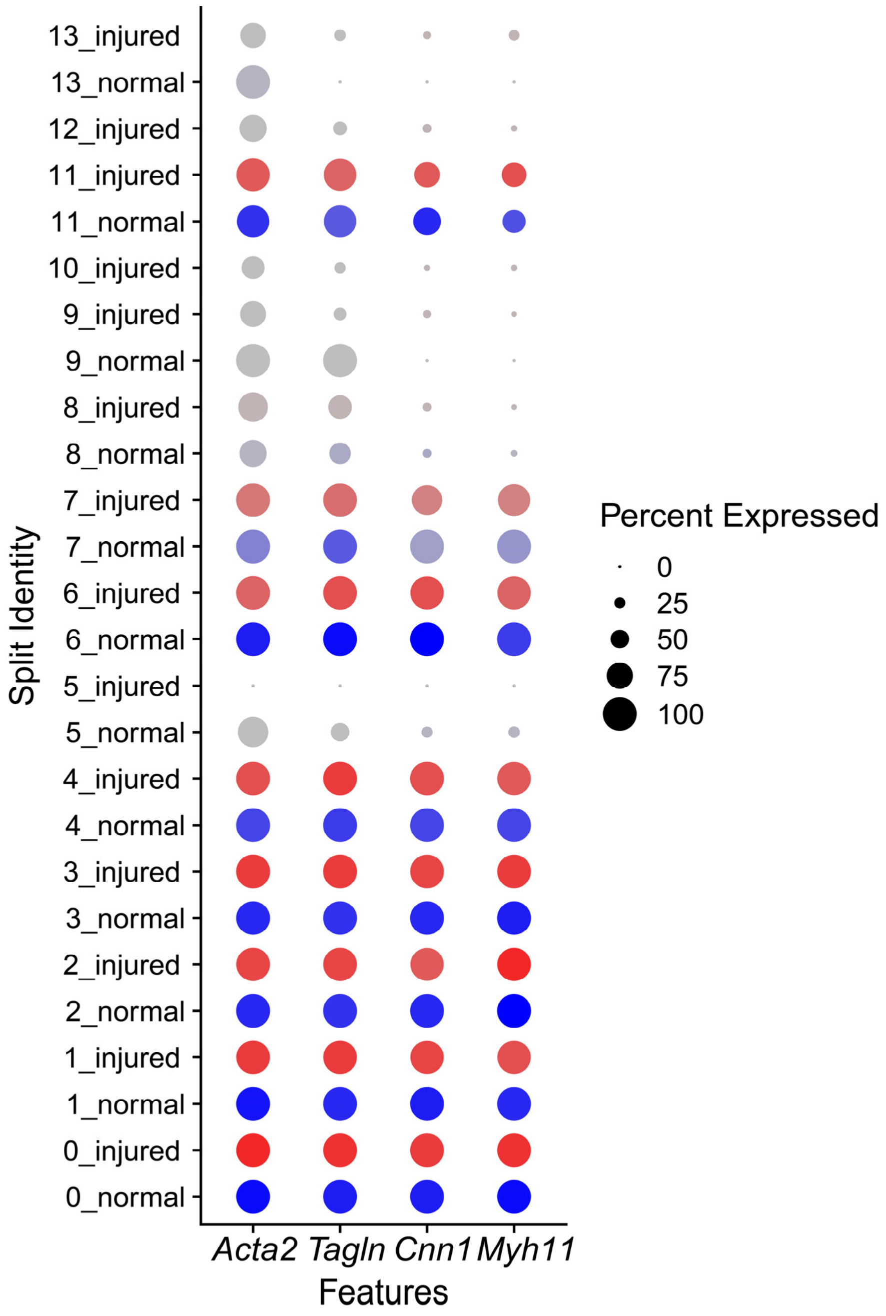

Figure S9. Dot plots showing the expression of selected genes (X-axis) in each cluster (Y-axis) in normal and injured arteries. The size of the dot represents the percentage of cells expressing the gene and the color represents the average normalized expression level (scaled and centered). Dot plots showed downregulation of contractile SMCs genes in cluster 8.

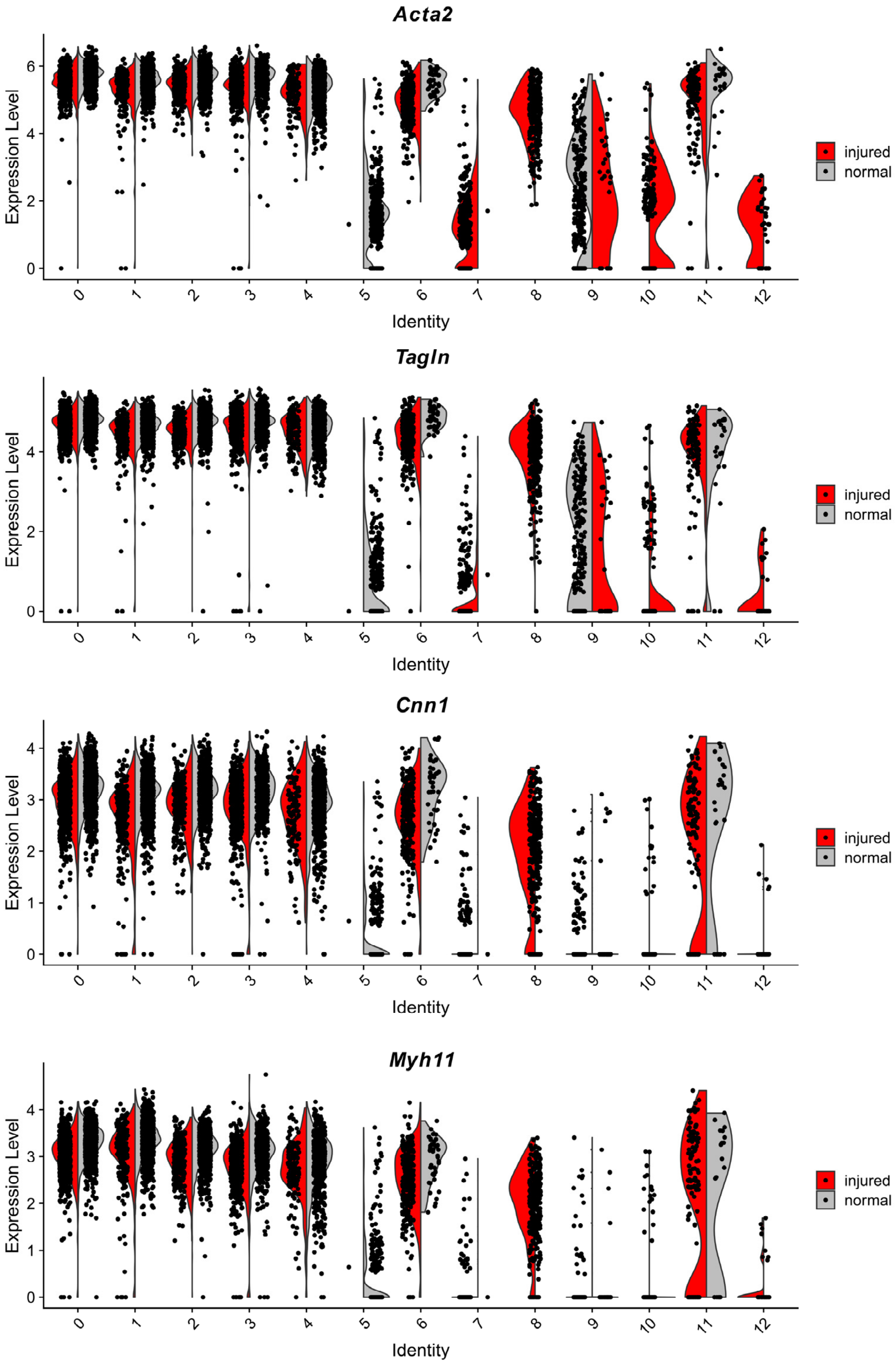

Figure S10. Violin plots of selected genes of each cluster in normal and injured arteries.

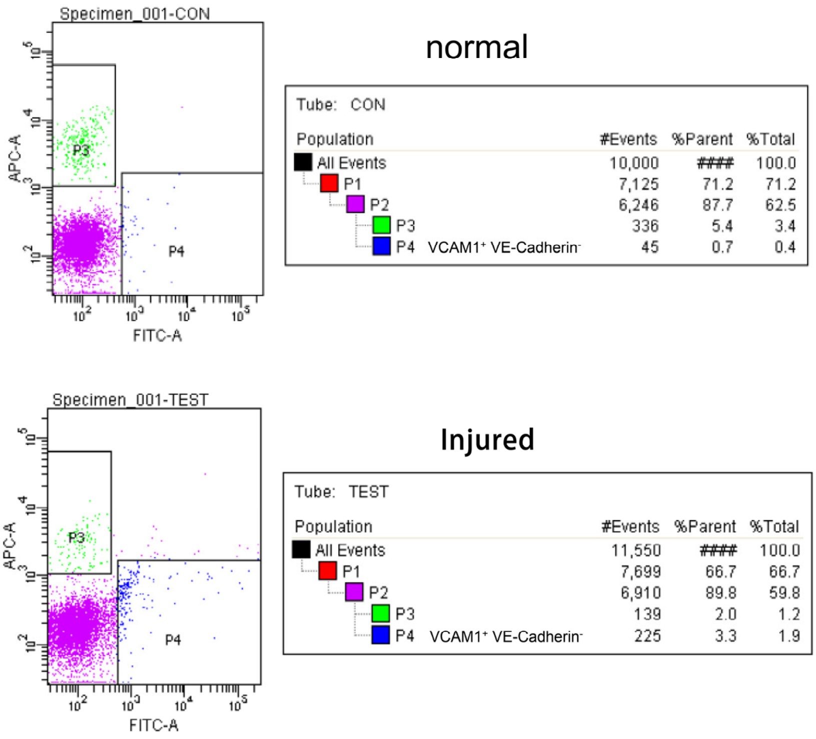

Figure S11. Dot plots showing the selection and isolation of VCAM1^{+} VE-Cadherin^{-} cells from normal and carotid arteries ligated for 7 days by FACS.

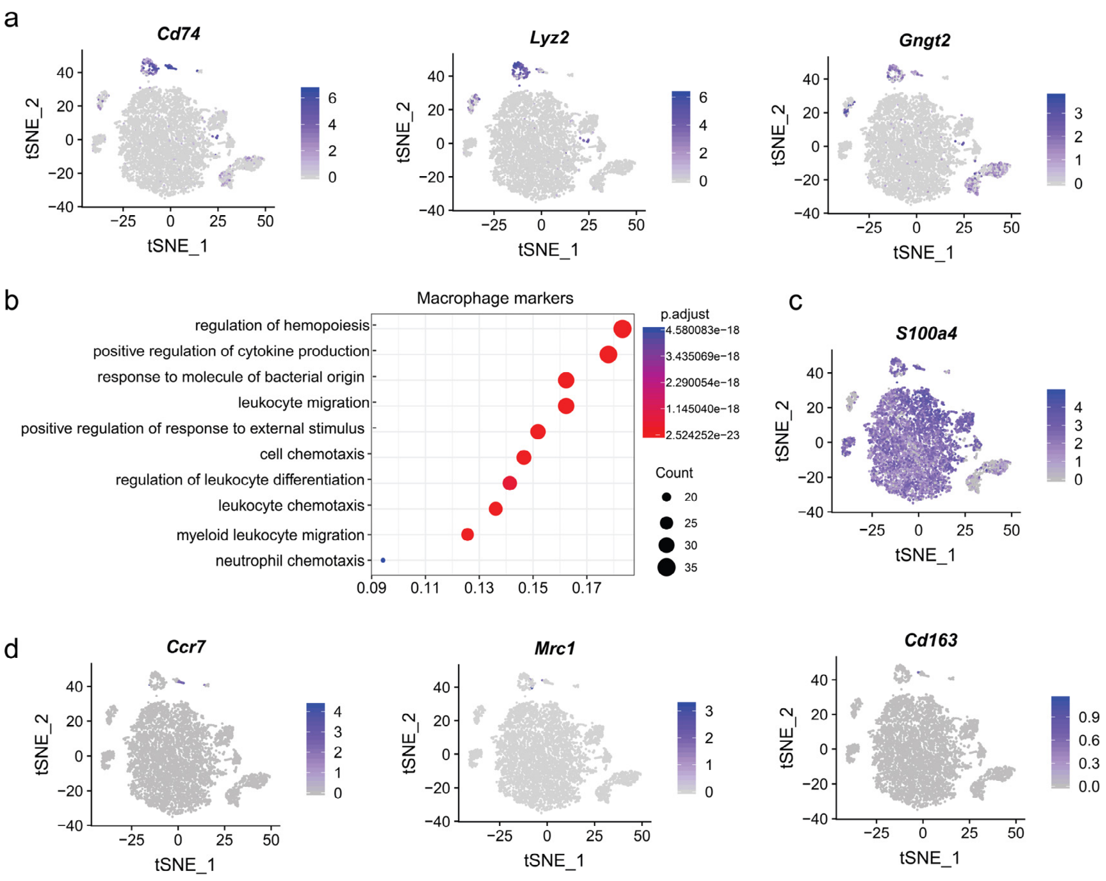

Figure S12. Gene expression in macrophage cluster. a Feature plot of selected inflammatory macrophage genes. **b** GO term (biological pathway) analysis of macrophage with its marker genes. **c** Feature plot of selected markers *S100a4* in normal and injured arteries. **d** Feature plot of M1 macrophage marker *Ccr7* and M2 macrophage marker *Mrc1* and *Cd163*.

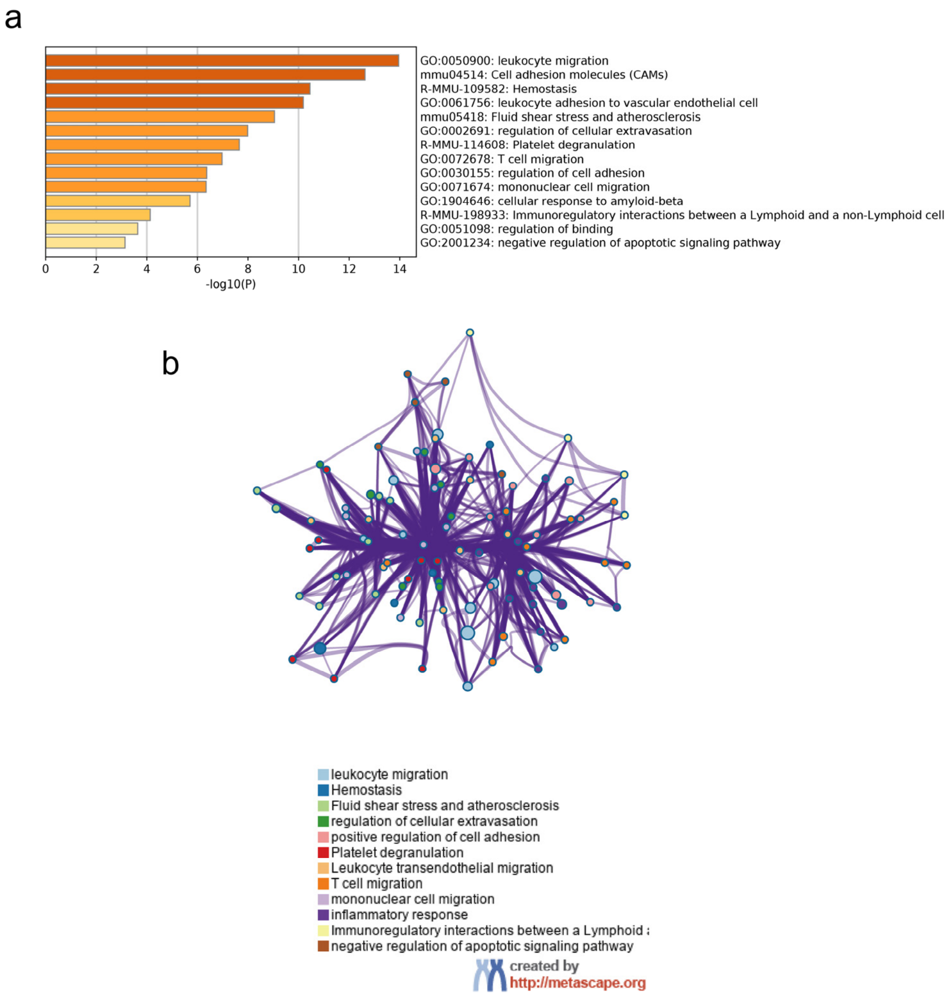

Figure S13. The cellular communication between ECs and macrophages. **a** Enrichment heatmap of the highly expressed gene list of ligand-receptor pairs between cluster 9 and cluster 7, colored by p-values. **b** Enrichment network of the highly expressed gene list of ligand-receptor pairs between cluster 9 and cluster 7. Each term is indicated by a circular node. The number of input genes falling into that term is represented by the circle size, and the cluster identities are represented by colors.

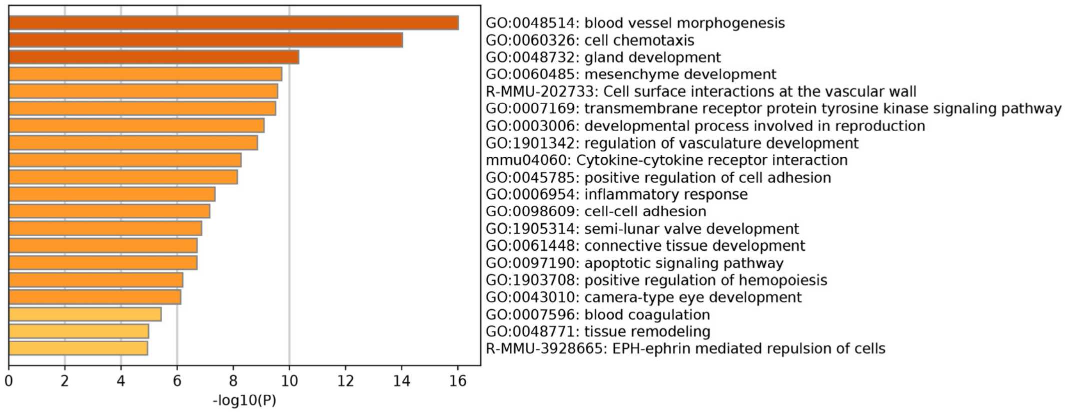

Figure S14. Interactions between cluster 8 SMCs and ECs. Enrichment heatmap of the highly expressed gene list of ligand-receptor pairs between cluster 9 and cluster 8, colored by p-values.

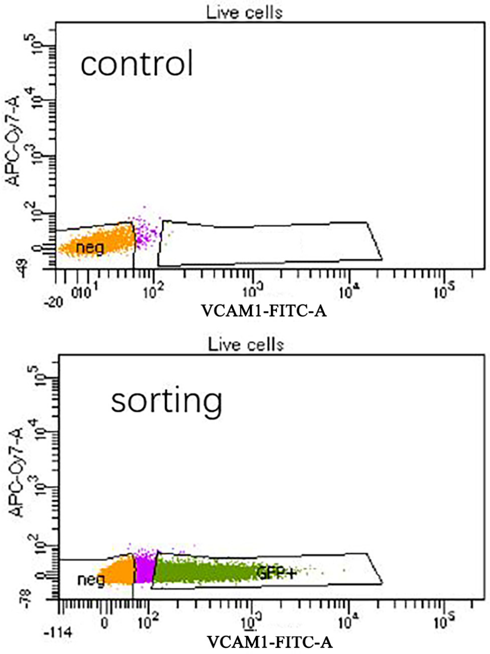

Figure S15. Dot plots showing the selection and isolation of VCAM1^{+} SMCs and VCAM1^{-} SMCs by FACS.

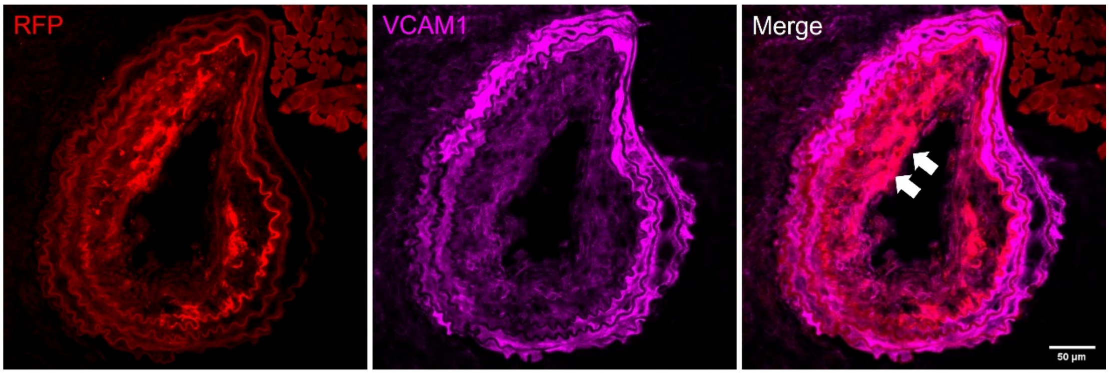

Figure S16. Carotid arterial cryosection containing RFP-expressing SMC-derived cells, co-stained for VCAM1. RFP-labeled SMC-derived cell was the dominated clone. Arrows point to the labeled VSMCs with VCAM1 expression. Scale bar: 50 µm.

Table S1. List of highly expressed genes identified in SMC clusters

p_val	avg_log2FC	pct.1	pct.2	p_val_adj	cluster	gene
3.86E-115	-0.8760076	0.819	0.912	6.05E-111	0	Junb
8.27E-96	-0.7840009	0.748	0.848	1.30E-91	0	Nr4a1
3.23E-87	-0.7838637	0.66	0.813	5.06E-83	0	Egr1
1.51E-85	-0.7704268	0.501	0.709	2.37E-81	0	Klf4
2.44E-70	-0.7954659	0.42	0.631	3.83E-66	0	Fosb
1.89E-66	-0.6023767	0.666	0.798	2.97E-62	0	Ier2
7.21E-63	-0.6243141	0.784	0.842	1.13E-58	0	Btg2
1.85E-61	0.27565533	0.999	0.998	2.90E-57	0	Acta2
3.45E-61	0.27677568	0.999	0.995	5.42E-57	0	Myl9
4.59E-50	-0.4882135	0.458	0.628	7.19E-46	0	Ppp1r15a
3.75E-49	-0.7782843	0.101	0.254	5.87E-45	0	Emp1
5.96E-48	-0.614374	0.974	0.975	9.35E-44	0	Fos
3.67E-47	0.26900656	0.941	0.801	5.75E-43	0	Sost
2.85E-44	-0.5944922	0.42	0.531	4.47E-40	0	Fxyd5
1.74E-43	-0.5181252	0.388	0.535	2.73E-39	0	Nr4a2
7.39E-40	-0.6817805	0.425	0.56	1.16E-35	0	Atf3
3.71E-39	-0.6275978	0.474	0.62	5.82E-35	0	Klf2
3.72E-39	0.29335108	0.983	0.949	5.83E-35	0	Rbp1
5.32E-38	-0.3629184	0.188	0.345	8.35E-34	0	Csrnp1
2.06E-36	0.33864012	0.797	0.64	3.23E-32	0	Mylk4
2.59E-35	-0.7360189	0.38	0.484	4.06E-31	0	Rgs2
3.09E-35	-0.4534238	0.474	0.607	4.84E-31	0	Zfp36
2.18E-34	0.35025153	0.963	0.912	3.42E-30	0	Mfap4
5.71E-34	-0.7556978	0.533	0.625	8.96E-30	0	Hbegf
5.07E-33	-0.5862492	0.118	0.244	7.94E-29	0	Ptgs2
2.23E-32	-0.4648981	0.126	0.244	3.50E-28	0	Crlf1
4.25E-32	0.39094942	0.81	0.689	6.66E-28	0	Rbp4
7.28E-32	-0.9814285	0.076	0.18	1.14E-27	0	Vcam1
9.24E-32	-0.5323745	0.928	0.927	1.45E-27	0	S100a10
4.27E-31	0.26110651	0.982	0.953	6.70E-27	0	Rock1
1.15E-30	0.2676788	0.922	0.823	1.81E-26	0	Ogn
1.59E-30	-0.3437145	0.83	0.864	2.49E-26	0	Rtn4
1.89E-30	-0.3114466	0.986	0.982	2.97E-26	0	Ftl1
4.38E-30	-0.7543071	0.848	0.85	6.87E-26	0	Fn1
8.57E-30	0.336549	0.84	0.72	1.34E-25	0	Fbxl22
1.28E-29	-0.4766472	0.469	0.601	2.01E-25	0	Ier3
6.66E-29	-0.501524	0.265	0.415	1.04E-24	0	Cdkn1a
2.07E-28	0.25455407	0.982	0.953	3.25E-24	0	Filip1l
1.30E-27	-0.4681213	0.705	0.784	2.04E-23	0	Jun
2.36E-27	-1.163304	0.229	0.332	3.69E-23	0	Timp1
3.84E-27	-0.4347648	0.627	0.699	6.02E-23	0	Sgk1
4.22E-27	-0.4673469	0.915	0.917	6.61E-23	0	Ctgf
1.92E-26	-0.2763182	0.991	0.988	3.01E-22	0	H3f3b
3.09E-26	-0.3804764	0.77	0.815	4.85E-22	0	Klf6
6.43E-26	-0.3235586	0.398	0.522	1.01E-21	0	Ccnl1
7.93E-26	-0.4052103	0.681	0.741	1.24E-21	0	Gadd45b
2.50E-25	0.2632145	0.961	0.922	3.91E-21	0	Msrb1
3.61E-25	-1.3969791	0.78	0.803	5.66E-21	0	Mt2
4.04E-25	-0.4120578	0.961	0.956	6.34E-21	0	Anxa2

4.84E-25	-0.3337008	0.31	0.424	7.59E-21	0	Pde4b
1.07E-24	-0.4386132	0.328	0.452	1.67E-20	0	Ier5
1.98E-24	0.26026153	0.795	0.685	3.10E-20	0	St5
2.18E-24	-0.3339202	0.216	0.338	3.42E-20	0	Socs3
4.87E-24	-0.3272781	0.582	0.654	7.63E-20	0	Col6a3
4.97E-24	-0.3103938	0.306	0.438	7.79E-20	0	Tob1
1.20E-23	-0.4958914	0.512	0.561	1.87E-19	0	Flnc
1.42E-23	-0.3344752	0.95	0.952	2.23E-19	0	Dusp1
8.89E-23	-0.5793964	0.382	0.483	1.39E-18	0	Errfi1
9.06E-23	-0.3486194	0.681	0.741	1.42E-18	0	Adamts1
1.65E-21	-0.6322259	0.72	0.763	2.58E-17	0	Serpine1
5.59E-21	-0.3319987	0.2	0.287	8.77E-17	0	Plaur
8.44E-21	-0.2786758	0.936	0.944	1.32E-16	0	Col1a1
8.46E-21	-0.3064113	0.701	0.736	1.33E-16	0	Aqp1
3.28E-20	-0.4134146	0.913	0.913	5.14E-16	0	Cyr61
5.68E-20	-0.3301872	0.52	0.602	8.91E-16	0	Gem
9.36E-20	-0.3124872	0.663	0.725	1.47E-15	0	Lox
3.50E-19	-0.2560504	0.873	0.869	5.48E-15	0	Tmsb10
6.69E-19	-0.2584952	0.529	0.632	1.05E-14	0	Sertad1
7.86E-19	0.30223261	0.503	0.395	1.23E-14	0	Wif1
8.14E-18	-0.4235737	0.645	0.671	1.28E-13	0	Lgals3
1.60E-17	0.25501905	0.698	0.594	2.51E-13	0	[illegible]030013I19Ri[illegible]
3.00E-17	-0.2662977	0.796	0.82	4.71E-13	0	Col15a1
3.69E-17	-0.2759614	0.138	0.221	5.79E-13	0	Cd44
5.87E-17	-0.2731737	0.996	0.993	9.20E-13	0	Tm4sf1
2.35E-16	-0.2653165	0.318	0.411	3.68E-12	0	Cd109
1.92E-15	-0.7511946	0.986	0.974	3.01E-11	0	Mt1
4.12E-15	-0.255695	0.477	0.547	6.46E-11	0	Map2k3
1.08E-14	-0.326056	0.685	0.725	1.69E-10	0	Nfkbia
1.17E-14	-0.271137	0.169	0.25	1.83E-10	0	Phlda1
1.91E-14	-0.3227087	0.735	0.744	2.99E-10	0	Id1
3.53E-14	-0.3690022	0.938	0.938	5.53E-10	0	S100a4
2.02E-13	-0.2559304	0.321	0.41	3.17E-09	0	Maff
1.00E-12	-0.2972472	0.989	0.98	1.57E-08	0	Eln
2.22E-12	-0.3950591	0.549	0.609	3.47E-08	0	Sncg
3.30E-12	-0.3124142	0.371	0.458	5.17E-08	0	Meg3
3.13E-11	-0.3934183	0.32	0.372	4.91E-07	0	Gm12840
6.14E-09	-0.2935571	0.756	0.783	9.63E-05	0	Gadd45g
9.06E-08	-0.252886	0.568	0.601	0.00142057	0	Cebpd
1.43E-05	-0.2780839	0.447	0.477	0.22391351	0	Dnajb1
0.00035318	-0.2577882	0.999	0.999	1	0	Mgp
0	0.63075735	0.999	0.999	0	1	mt-Atp6
0	0.61129743	0.999	0.999	0	1	mt-Co2
0	0.60896798	0.999	0.998	0	1	mt-Nd4
0	0.60595774	0.999	0.999	0	1	mt-Cytb
[illegible]65645841247	0.59193227	1	0.999	[illegible]44339243659[illegible]	1	mt-Co3
1.30E-292	0.57432499	0.999	0.999	2.04E-288	1	mt-Nd1
6.73E-290	0.58167615	0.999	0.999	1.06E-285	1	mt-Co1
4.90E-228	0.55559327	0.997	0.997	7.69E-224	1	mt-Nd2
2.04E-144	0.52174535	0.997	0.994	3.21E-140	1	Flna
1.51E-121	0.52007812	0.991	0.982	2.37E-117	1	mt-Nd5

6.40E-121	0.57725244	0.962	0.859	1.00E-116	1	mt-Nd4l
9.51E-89	-0.3283766	0.976	0.994	1.49E-84	1	Rpl3
1.21E-84	0.43966896	0.996	0.991	1.90E-80	1	Myh11
2.28E-84	-0.278323	0.993	0.998	3.57E-80	1	Rps3
9.66E-74	-0.2557106	0.996	0.997	1.52E-69	1	Rps3a1
1.44E-73	-0.2610295	0.992	0.997	2.25E-69	1	Rplp0
1.81E-73	-0.2596054	0.994	0.998	2.84E-69	1	Rps4x
1.98E-66	0.39932553	0.99	0.974	3.11E-62	1	Tns1
7.61E-65	0.3683288	0.993	0.987	1.19E-60	1	Itga8
3.80E-53	0.37799187	0.991	0.978	5.96E-49	1	mt-Nd3
1.01E-48	-0.3195594	0.983	0.991	1.59E-44	1	Gapdh
2.77E-47	0.42740156	0.773	0.606	4.34E-43	1	Marveld1
6.25E-46	0.36250047	0.948	0.897	9.80E-42	1	Lamb2
9.22E-44	-0.2607141	0.995	0.998	1.45E-39	1	Bgn
2.79E-43	-0.3027199	0.989	0.993	4.38E-39	1	Cd63
9.18E-42	0.32930656	0.965	0.935	1.44E-37	1	Cped1
1.58E-41	0.43566758	0.981	0.941	2.48E-37	1	Nov
5.12E-41	0.3676689	0.914	0.841	8.03E-37	1	Synpo2
4.77E-40	0.35800035	0.838	0.698	7.47E-36	1	Ppp1r12b
3.15E-37	0.34657822	0.916	0.831	4.94E-33	1	Adcy5
4.10E-35	0.28875979	0.949	0.916	6.43E-31	1	Dst
3.04E-33	0.30391901	0.99	0.973	4.76E-29	1	Col3a1
7.32E-33	0.40840959	0.8	0.652	1.15E-28	1	Mylk4
1.62E-31	0.30200814	0.977	0.944	2.53E-27	1	Xist
2.16E-29	0.68865853	0.85	0.791	3.38E-25	1	Gm42418
8.68E-29	-0.3502702	0.992	0.994	1.36E-24	1	Tm4sf1
1.04E-28	0.28008325	0.892	0.851	1.63E-24	1	Col4a1
1.12E-28	0.35255824	0.808	0.7	1.76E-24	1	Itga9
1.67E-28	0.28474875	0.873	0.782	2.62E-24	1	Col4a2
7.96E-28	0.30601904	0.538	0.366	1.25E-23	1	Dact3
6.58E-27	0.29312539	0.958	0.926	1.03E-22	1	Ltbp4
5.25E-26	0.29822582	0.456	0.299	8.23E-22	1	Wtip
5.25E-26	-0.3539849	0.479	0.576	8.23E-22	1	Tuba1c
4.71E-25	0.40518447	0.616	0.47	7.39E-21	1	Nr4a2
1.74E-24	-0.4089211	0.997	0.998	2.73E-20	1	Hspa8
1.37E-23	0.30387393	0.908	0.834	2.14E-19	1	Ogn
1.64E-23	0.36428432	0.894	0.812	2.57E-19	1	Btg2
3.15E-22	0.29658182	0.88	0.794	4.94E-18	1	Mcam
5.26E-22	0.27271385	0.784	0.672	8.25E-18	1	Jph2
1.01E-21	0.26200746	0.34	0.204	1.59E-17	1	Pkdcc
1.15E-21	0.26303399	0.86	0.777	1.81E-17	1	Fam129a
2.41E-21	-0.3209735	0.573	0.642	3.78E-17	1	Tubb6
3.30E-21	0.27154013	0.871	0.798	5.17E-17	1	Slc38a2
4.27E-21	0.26844233	0.836	0.763	6.69E-17	1	Myh10
2.84E-20	0.26821607	0.801	0.693	4.45E-16	1	Sorl1
5.05E-20	0.27326065	0.57	0.426	7.91E-16	1	mt-Atp8
9.76E-20	0.29663604	0.876	0.811	1.53E-15	1	Nr4a1
3.07E-19	0.27681794	0.59	0.447	4.81E-15	1	Myom1
3.58E-19	-0.4075992	0.915	0.932	5.61E-15	1	Tnfrsf12a
4.19E-19	-1.0413131	0.232	0.324	6.56E-15	1	Timp1
2.08E-18	0.26308474	0.757	0.668	3.26E-14	1	Pkp4

4.46E-18	0.26377101	0.686	0.562	6.99E-14	1	Plin4
5.09E-18	0.25830229	0.286	0.167	7.99E-14	1	Igfbp5
9.02E-18	0.25832445	0.836	0.775	1.41E-13	1	Enah
2.42E-16	-0.5972943	0.541	0.616	3.79E-12	1	Hbegf
2.60E-16	-0.2656408	0.876	0.925	4.08E-12	1	Ldha
4.26E-16	-0.2629375	0.658	0.734	6.68E-12	1	Eno1
2.29E-14	0.25298731	0.824	0.733	3.58E-10	1	Fbxl22
4.24E-14	-0.2593579	0.312	0.405	6.64E-10	1	Maff
5.20E-14	-0.4888596	0.162	0.228	8.15E-10	1	Emp1
1.69E-12	-0.2840697	0.929	0.947	2.65E-08	1	Ifitm3
1.05E-11	-0.3423611	0.62	0.675	1.65E-07	1	Lgals3
2.24E-11	-0.2757356	0.692	0.771	3.51E-07	1	Apoe
2.27E-10	-0.2779092	0.739	0.767	3.57E-06	1	Sdc4
4.03E-10	0.29366079	0.54	0.439	6.33E-06	1	Rgs2
4.35E-10	-0.7350998	0.124	0.161	6.82E-06	1	Vcam1
1.70E-09	-0.3070727	0.699	0.754	2.67E-05	1	Ifrd1
1.89E-09	-0.2744013	0.942	0.961	2.96E-05	1	Anxa2
1.99E-09	-0.3068342	0.91	0.931	3.12E-05	1	S100a10
3.38E-09	-0.2895509	0.889	0.899	5.31E-05	1	Dnaja1
4.21E-09	-0.2705771	0.388	0.434	6.60E-05	1	Slc39a1
8.09E-09	-0.2611659	0.472	0.511	0.00012687	1	Fxyd5
1.75E-08	-1.1557593	0.791	0.799	0.00027519	1	Mt2
3.53E-08	-0.2750784	0.455	0.472	0.00055343	1	Dnajb1
8.42E-08	-0.2707224	0.918	0.943	0.00131975	1	S100a4
4.14E-07	-0.325069	0.96	0.976	0.00648801	1	Cryab
1.25E-06	-0.3098444	0.942	0.946	0.01963665	1	Clu
1.33E-06	-0.3522522	0.451	0.46	0.02091779	1	Errfi1
1.70E-06	-0.2603467	0.913	0.945	0.0267257	1	Pam
1.64E-05	-0.268016	0.999	0.999	0.25698701	1	Mgp
3.08E-05	-0.3719222	0.989	0.994	0.48350108	1	Hspb1
0.00043584	-0.423691	0.819	0.822	1	1	Hspa1a
1.49E-248	1.07085216	0.998	0.969	2.34E-244	2	Fos
1.28E-216	1.00234632	0.98	0.788	2.01E-212	2	Nr4a1
7.79E-138	0.76017616	0.969	0.796	1.22E-133	2	Btg2
9.65E-124	0.85248794	0.906	0.705	1.51E-119	2	Id1
8.32E-122	0.78253937	0.978	0.869	1.30E-117	2	Junb
6.59E-101	-1.312533	0.764	0.868	1.03E-96	2	Fn1
6.84E-101	0.64810706	0.994	0.965	1.07E-96	2	Id3
1.06E-95	0.66713439	0.91	0.732	1.66E-91	2	Ier2
1.93E-91	0.66673846	0.924	0.742	3.03E-87	2	Egr1
3.07E-87	0.64158753	0.908	0.732	4.81E-83	2	Jun
1.71E-86	0.72620567	0.978	0.899	2.68E-82	2	Cyr61
3.22E-86	0.59644807	0.986	0.944	5.05E-82	2	Dusp1
7.07E-86	0.79207627	0.751	0.476	1.11E-81	2	Atf3
7.63E-84	-0.9386654	0.304	0.548	1.20E-79	2	Fxyd5
2.01E-83	-0.7308117	0.951	0.959	3.15E-79	2	Anxa2
2.51E-82	0.67003787	0.841	0.615	3.93E-78	2	Klf4
1.65E-81	-0.7224138	0.872	0.92	2.59E-77	2	Anxa1
6.77E-80	0.43734372	0.999	0.982	1.06E-75	2	Ubc
1.39E-79	-0.7107011	0.853	0.893	2.18E-75	2	Prss23
9.84E-77	0.62238631	0.951	0.863	1.54E-72	2	Id2

1.31E-76	0.51805704	0.972	0.805	2.06E-72	2	Sost
4.22E-72	-0.4388115	0.998	0.998	6.63E-68	2	S100a6
3.80E-71	0.74897903	0.766	0.542	5.96E-67	2	Klf2
3.78E-65	0.88674755	0.639	0.417	5.93E-61	2	Rgs2
9.45E-63	-0.6959314	0.66	0.798	1.48E-58	2	Itga5
3.47E-62	0.5595097	0.696	0.453	5.43E-58	2	Nr4a2
2.61E-61	-0.8554588	0.545	0.691	4.10E-57	2	Lgals3
8.43E-61	0.67384096	0.881	0.753	1.32E-56	2	Gadd45g
2.72E-58	-0.4478345	0.973	0.965	4.26E-54	2	Cyb5r3
2.69E-56	0.33518922	0.997	0.977	4.22E-52	2	Map1b
1.18E-55	-0.7333143	0.913	0.93	1.86E-51	2	S100a10
2.38E-54	0.50004782	0.743	0.536	3.73E-50	2	Zfp36
2.77E-54	0.50251664	0.742	0.529	4.34E-50	2	Ier3
1.89E-50	-0.3055239	0.998	0.997	2.96E-46	2	S100a11
3.88E-50	0.40955335	0.759	0.538	6.09E-46	2	Fosb
3.92E-50	0.47412162	0.746	0.549	6.14E-46	2	Ppp1r15a
1.24E-49	0.57423579	0.882	0.728	1.95E-45	2	Apoe
2.25E-49	0.42963676	0.964	0.875	3.53E-45	2	Ckb
3.99E-47	-0.6234912	0.926	0.941	6.25E-43	2	S100a4
2.32E-44	0.54178442	0.571	0.413	3.64E-40	2	Rasl11a
2.56E-44	0.39390422	0.985	0.94	4.02E-40	2	Nov
1.84E-43	0.42678981	0.643	0.457	2.89E-39	2	Ccnl1
3.04E-43	-0.4532259	0.78	0.822	4.76E-39	2	Col15a1
2.33E-42	0.2894077	0.994	0.964	3.65E-38	2	Ppp1r12a
1.47E-40	0.26790971	0.996	0.976	2.31E-36	2	Ddx5
3.23E-40	-0.4926847	0.64	0.747	5.07E-36	2	Aqp1
2.91E-39	0.32392458	0.983	0.948	4.57E-35	2	Cnn3
6.44E-39	0.33396779	0.576	0.367	1.01E-34	2	Tob1
1.18E-38	-0.4828901	0.128	0.296	1.86E-34	2	Plaur
7.22E-38	-1.0211028	0.046	0.178	1.13E-33	2	Vcam1
8.29E-38	-0.5249262	0.09	0.243	1.30E-33	2	Crlf1
6.09E-37	-0.4398434	0.835	0.878	9.54E-33	2	Tmsb10
1.58E-36	0.35945847	0.853	0.718	2.47E-32	2	Limch1
1.62E-36	-0.4326888	0.245	0.42	2.54E-32	2	Cd109
3.18E-36	-0.7464949	0.098	0.242	4.99E-32	2	Emp1
6.67E-36	-0.2817037	0.987	0.971	1.05E-31	2	Emp3
7.05E-36	-0.3030495	0.992	0.984	1.11E-31	2	Tspo
2.12E-35	-0.3202573	0.909	0.919	3.32E-31	2	Ltbp1
2.29E-35	0.3534175	0.462	0.271	3.59E-31	2	Csrnp1
2.32E-35	-1.2344515	0.179	0.335	3.64E-31	2	Timp1
2.58E-35	0.28631075	0.959	0.871	4.04E-31	2	Wfdc1
9.54E-35	0.38842888	0.436	0.253	1.50E-30	2	Timp4
1.54E-34	-0.4294636	0.348	0.52	2.42E-30	2	Itgbl1
2.00E-34	-0.4381532	0.589	0.713	3.13E-30	2	Ahnak2
3.29E-34	0.51615765	0.871	0.768	5.15E-30	2	Rrad
4.15E-34	0.38752032	0.458	0.274	6.51E-30	2	Socs3
1.08E-33	0.40150859	0.711	0.553	1.70E-29	2	Gem
6.88E-33	0.36004193	0.49	0.302	1.08E-28	2	Enpp2
7.14E-33	0.44442109	0.392	0.228	1.12E-28	2	Art3
3.76E-32	-0.6471339	0.451	0.571	5.90E-28	2	Flnc
1.33E-31	0.29681172	0.978	0.94	2.08E-27	2	Ncam1

2.62E-31	0.35576366	0.729	0.569	4.11E-27	2	Net1
2.76E-31	-0.4435171	0.962	0.967	4.33E-27	2	Nupr1
6.92E-31	0.42233996	0.81	0.707	1.08E-26	2	Gadd45b
9.50E-31	0.33558348	0.728	0.578	1.49E-26	2	Pnrc1
1.18E-30	0.4517323	0.732	0.572	1.85E-26	2	Tcap
1.68E-30	-0.2553501	1	0.998	2.64E-26	2	Lgals1
1.81E-30	0.37979467	0.76	0.613	2.84E-26	2	Fibin
1.18E-28	0.25889126	0.975	0.937	1.85E-24	2	Fbln5
1.04E-27	-0.3589779	0.665	0.748	1.63E-23	2	Col5a2
3.82E-27	0.28199276	0.846	0.729	5.98E-23	2	Ecm2
1.17E-26	0.39244922	0.219	0.106	1.84E-22	2	Rasd1
3.94E-26	-0.2831746	0.967	0.964	6.18E-22	2	Ahnak
7.92E-26	0.27607829	0.733	0.599	1.24E-21	2	Idh2
1.47E-25	-0.3798137	0.564	0.653	2.30E-21	2	Col6a3
7.29E-25	0.26562608	0.804	0.689	1.14E-20	2	Ldhb
1.55E-24	0.2903891	0.853	0.746	2.43E-20	2	Rhob
3.92E-24	0.30126342	0.928	0.844	6.14E-20	2	Sparcl1
1.10E-23	0.32618324	0.431	0.291	1.73E-19	2	Hes1
1.65E-23	-0.3572195	0.24	0.38	2.59E-19	2	Pcolce2
4.00E-23	-0.2735631	0.934	0.941	6.27E-19	2	Pmepa1
6.64E-23	-0.3270188	0.466	0.584	1.04E-18	2	Sulf1
7.30E-23	-0.3127058	0.196	0.331	1.14E-18	2	Sgms2
1.17E-22	-0.2961371	0.757	0.814	1.83E-18	2	Rock2
5.18E-22	0.2875293	0.703	0.585	8.13E-18	2	Sertad1
5.23E-22	-0.5546508	0.5	0.615	8.20E-18	2	Sncg
6.23E-22	0.25234349	0.906	0.821	9.77E-18	2	Selenop
7.97E-22	-0.2514126	0.977	0.98	1.25E-17	2	Aebp1
8.17E-22	-0.273629	0.803	0.833	1.28E-17	2	Tpm4
2.77E-21	0.26376886	0.915	0.833	4.34E-17	2	Ogn
3.37E-21	0.28971275	0.825	0.704	5.29E-17	2	Adamts1
3.65E-21	-0.3162456	0.846	0.858	5.73E-17	2	Rtn4
3.73E-21	0.26387241	0.531	0.385	5.85E-17	2	Asb2
1.21E-20	-0.269058	0.968	0.952	1.90E-16	2	Cav1
2.91E-20	-0.3618566	0.488	0.562	4.56E-16	2	2200002D01Ril
1.04E-19	-0.3190387	0.996	0.993	1.63E-15	2	Tm4sf1
1.22E-19	-0.2717272	0.851	0.885	1.92E-15	2	mt-Nd4l
1.32E-19	-0.3219421	0.326	0.459	2.07E-15	2	Ace
1.75E-19	-0.3111534	0.401	0.523	2.75E-15	2	Cav3
8.52E-19	0.25596494	0.458	0.325	1.34E-14	2	Rgs7bp
8.99E-19	-0.631259	0.735	0.817	1.41E-14	2	Gm42418
1.28E-18	0.25570079	0.538	0.394	2.00E-14	2	Ier5
2.26E-18	0.29356624	0.692	0.567	3.54E-14	2	Stbd1
4.01E-18	0.29418031	0.908	0.858	6.28E-14	2	Actg2
6.61E-18	-0.4399175	0.944	0.945	1.04E-13	2	Clu
7.02E-18	0.27108241	0.666	0.557	1.10E-13	2	Tes
7.99E-18	-0.3496569	0.94	0.945	1.25E-13	2	Ifitm3
9.30E-18	-0.2720811	0.225	0.344	1.46E-13	2	Prex2
1.40E-17	-0.3070777	0.973	0.971	2.19E-13	2	Mustn1
1.93E-17	-0.2978194	0.942	0.942	3.03E-13	2	Col1a1
2.69E-17	0.25569397	0.641	0.512	4.23E-13	2	Tob2
4.11E-17	-0.2959846	0.121	0.218	6.45E-13	2	Cd44

4.54E-17	-0.2626723	0.432	0.534	7.13E-13	2	Fads3
2.06E-15	-0.3692467	0.917	0.931	3.23E-11	2	Tnfrsf12a
1.75E-14	0.2708473	0.628	0.537	2.74E-10	2	Bambi
2.58E-14	-0.2561353	0.507	0.586	4.05E-10	2	Plod2
3.95E-14	-0.3946206	0.906	0.919	6.19E-10	2	Ctgf
2.09E-11	-0.5235198	0.734	0.756	3.28E-07	2	Serpine1
3.26E-11	-0.3656854	0.979	0.972	5.12E-07	2	Cryab
1.44E-10	-0.2949155	0.658	0.706	2.26E-06	2	Gm13889
2.84E-10	0.3965204	0.219	0.147	4.45E-06	2	Des
1.31E-09	-0.2990581	0.738	0.767	2.05E-05	2	Sdc4
2.18E-09	-0.3684746	0.305	0.367	3.42E-05	2	Hspa1b
6.66E-08	-1.0878217	0.815	0.793	0.00104369	2	Mt2
9.76E-07	-0.2535121	0.369	0.438	0.01531077	2	Slc39a1
1.44E-05	-0.2537202	0.988	0.98	0.22631353	2	Eln
1.98E-174	-0.4807993	0.996	1	3.11E-170	3	mt-Atp6
1.35E-165	-0.4871544	0.995	1	2.12E-161	3	mt-Cytb
1.13E-162	-0.4691013	0.996	1	1.77E-158	3	mt-Co3
1.35E-150	-0.4746986	0.996	0.999	2.11E-146	3	mt-Co1
4.56E-146	-0.455085	0.995	1	7.15E-142	3	mt-Co2
3.01E-144	-0.4655474	0.991	1	4.71E-140	3	mt-Nd4
6.48E-133	-0.441466	0.993	1	1.02E-128	3	mt-Nd1
2.31E-111	-0.4408651	0.985	0.999	3.63E-107	3	mt-Nd2
2.97E-74	-0.4551689	0.941	0.992	4.65E-70	3	mt-Nd5
4.31E-49	-0.4136934	0.732	0.905	6.77E-45	3	mt-Nd4l
4.37E-45	-0.3363872	0.932	0.99	6.86E-41	3	mt-Nd3
5.26E-43	-0.9133755	0.748	0.867	8.24E-39	3	Fn1
9.50E-39	-0.32523	0.984	0.997	1.49E-34	3	Flna
3.33E-37	-0.651712	0.843	0.93	5.23E-33	3	Ctgf
4.29E-32	0.28153935	0.992	0.998	6.72E-28	3	Csrp2
2.21E-30	-0.5852472	0.379	0.579	3.46E-26	3	Flnc
1.07E-28	-0.2991725	0.659	0.824	1.67E-24	3	Col4a2
2.39E-28	0.35306601	0.37	0.336	3.75E-24	3	Pecam1
1.49E-27	-0.286475	0.742	0.88	2.34E-23	3	Col4a1
2.21E-26	0.26424511	0.982	0.994	3.47E-22	3	Serpine2
6.99E-26	0.53904631	0.804	0.748	1.10E-21	3	Apoe
2.15E-24	-0.3420865	0.483	0.664	3.38E-20	3	Col6a3
1.15E-22	0.27251256	0.975	0.988	1.80E-18	3	Gsn
1.33E-21	0.27514029	0.492	0.449	2.09E-17	3	Abi3bp
8.37E-20	-0.2601462	0.605	0.756	1.31E-15	3	Col5a2
1.72E-19	-0.4060224	0.11	0.233	2.70E-15	3	Crlf1
4.60E-19	-0.2768353	0.951	0.98	7.21E-15	3	Col3a1
8.02E-18	-0.2744403	0.1	0.218	1.26E-13	3	Cd44
2.34E-17	-0.8680164	0.07	0.169	3.66E-13	3	Vcam1
7.39E-16	-0.4604111	0.118	0.229	1.16E-11	3	Ptgs2
7.70E-16	0.25729662	0.704	0.679	1.21E-11	3	Osr1
3.18E-15	0.26704062	0.389	0.327	4.99E-11	3	Enpp2
1.60E-14	-0.5898914	0.132	0.23	2.50E-10	3	Emp1
2.03E-14	-0.283411	0.925	0.974	3.19E-10	3	Nupr1
3.49E-14	-0.2613277	0.775	0.87	5.47E-10	3	Rtn4
3.99E-14	-0.5760743	0.661	0.769	6.26E-10	3	Serpine1
4.93E-14	-0.8957194	0.698	0.815	7.73E-10	3	Mt2

6.82E-13	-0.9786703	0.218	0.322	1.07E-08	3	Timp1
7.66E-13	0.3180043	0.883	0.827	1.20E-08	3	Sost
5.71E-12	0.35262292	0.919	0.943	8.95E-08	3	Pam
6.89E-12	-0.3850247	0.255	0.373	1.08E-07	3	Hspa1b
7.84E-12	0.29278018	0.769	0.737	1.23E-07	3	Id1
2.58E-11	-0.392363	0.476	0.596	4.05E-07	3	Fosb
3.72E-09	-0.2999427	0.411	0.52	5.84E-05	3	Fxyd5
5.65E-08	-0.431406	0.948	0.982	0.00088666	3	Mt1
8.67E-08	-0.2984317	0.278	0.374	0.0013594	3	Gm12840
2.55E-07	-0.3739084	0.733	0.814	0.0040054	3	Gm42418
4.39E-07	-0.2580504	0.389	0.483	0.00688044	3	Dnajb1
5.43E-06	0.26643264	0.999	1	0.08521362	3	Actb
2.41E-05	3.26991687	0.104	0.091	0.37772442	3	Hbb-bs
7.05E-05	-0.361729	0.395	0.469	1	3	Errfi1
0	1.85656276	0.978	0.831	0	4	Fn1
4.87E-282	1.22247292	0.995	0.952	7.64E-278	4	Anxa2
3.14E-265	1.42688056	0.915	0.444	4.93E-261	4	Fxyd5
3.24E-252	0.79815814	1	0.998	5.08E-248	4	S100a6
1.18E-246	1.16556818	0.995	0.899	1.85E-242	4	Anxa1
8.75E-236	1.30786669	0.996	0.917	1.37E-231	4	S100a10
4.09E-227	0.6691111	1	0.998	6.41E-223	4	Lgals1
4.96E-220	-1.6982731	0.496	0.885	7.77E-216	4	Sost
1.54E-210	1.37454404	0.917	0.628	2.41E-206	4	Lgals3
1.58E-196	1.28788831	0.631	0.154	2.48E-192	4	Crlf1
2.96E-171	0.97074905	0.992	0.87	4.64E-167	4	Prss23
3.73E-154	1.09664108	0.986	0.931	5.85E-150	4	S100a4
1.18E-147	-0.6834493	0.999	0.998	1.85E-143	4	Cst3
3.78E-140	0.85956696	0.942	0.696	5.92E-136	4	Aqp1
7.09E-135	0.69920826	0.999	0.962	1.11E-130	4	Cyb5r3
2.32E-132	0.5216036	1	0.997	3.63E-128	4	S100a11
4.09E-132	0.74219816	0.992	0.95	6.41E-128	4	Cav1
6.94E-127	-0.8108714	0.927	0.958	1.09E-122	4	Cnn3
4.04E-126	0.81382643	0.744	0.336	6.33E-122	4	Cd109
1.17E-125	0.53279245	1	0.998	1.84E-121	4	Vim
1.98E-123	0.79226806	0.915	0.658	3.10E-119	4	Ahnak2
2.85E-109	-0.5447701	1	0.996	4.46E-105	4	Csrp2
4.34E-108	0.5650821	0.999	0.984	6.81E-104	4	Tspo
5.68E-105	0.61731332	0.37	0.069	8.90E-101	4	Bdh2
5.48E-104	0.68198897	0.97	0.856	8.59E-100	4	Tmsb10
7.97E-104	0.55532022	0.997	0.97	1.25E-99	4	Emp3
4.84E-101	0.76769645	0.874	0.602	7.58E-97	4	Col6a3
7.97E-100	0.57908543	0.995	0.96	1.25E-95	4	Ahnak
7.60E-97	0.51069407	1	0.99	1.19E-92	4	Cd81
2.30E-96	0.6796815	0.955	0.794	3.61E-92	4	Col15a1
1.86E-93	0.91602824	0.526	0.17	2.91E-89	4	Emp1
2.39E-90	-1.2859604	0.54	0.788	3.75E-86	4	Apoe
3.17E-90	0.7220663	0.946	0.748	4.97E-86	4	Itga5
5.04E-87	-0.6473895	0.876	0.953	7.90E-83	4	Fbln5
8.69E-87	0.62636631	0.632	0.312	1.36E-82	4	Capg
2.07E-86	0.76922068	0.819	0.51	3.25E-82	4	Flnc
3.08E-85	-0.5592477	0.939	0.968	4.83E-81	4	Rarres2

1.06E-83	0.5627544	0.94	0.817	1.67E-79	4	Uqcc2
8.80E-83	-1.1211006	0.323	0.642	1.38E-78	4	Tcap
2.75E-81	0.58858848	0.539	0.22	4.32E-77	4	Ppp1r14b
9.11E-81	-0.4015354	0.996	0.993	1.43E-76	4	Itm2b
4.26E-80	-0.7046175	0.934	0.965	6.68E-76	4	Filip1l
1.02E-77	-0.7205189	0.943	0.974	1.61E-73	4	Id3
3.22E-76	0.42825416	0.284	0.051	5.05E-72	4	Fbln2
1.98E-75	-0.5541578	0.915	0.943	3.11E-71	4	Slmap
3.39E-74	-0.5585385	0.913	0.943	5.32E-70	4	Oat
6.54E-73	0.54595755	0.995	0.969	1.03E-68	4	Lmna
4.23E-72	0.56995411	0.604	0.282	6.64E-68	4	Prex2
4.01E-69	0.55458403	0.96	0.84	6.29E-65	4	Rtn4
1.26E-68	-0.9693701	0.611	0.815	1.98E-64	4	Itih4
6.46E-68	0.51928727	0.904	0.715	1.01E-63	4	Dap
8.88E-67	-0.6245049	0.731	0.852	1.39E-62	4	Selenop
1.05E-66	-0.8813496	0.562	0.768	1.65E-62	4	Id1
2.92E-66	0.58120792	0.995	0.968	4.58E-62	4	Mustn1
5.84E-64	-0.5202627	0.992	0.992	9.16E-60	4	Serpine2
1.34E-63	0.58053145	0.74	0.452	2.10E-59	4	Itgbl1
1.39E-63	-0.4972732	0.986	0.98	2.17E-59	4	Map1b
1.15E-62	-1.0913846	0.141	0.42	1.81E-58	4	[illegible]210407C18Ri[illegible]
2.72E-62	0.45743592	0.997	0.984	4.26E-58	4	Txn1
4.90E-62	0.35256568	1	0.997	7.68E-58	4	Pfn1
1.76E-61	0.49950253	0.939	0.782	2.76E-57	4	Iqgap1
1.80E-60	-0.5898343	0.773	0.897	2.82E-56	4	Cd200
5.90E-60	0.53095072	0.894	0.684	9.25E-56	4	Uchl1
9.53E-60	-0.4782874	0.981	0.98	1.49E-55	4	Gstm1
7.08E-59	-0.3908484	0.977	0.976	1.11E-54	4	Rbpms
8.84E-59	-0.434042	0.969	0.969	1.39E-54	4	Ppp1r12a
9.86E-59	-0.656077	0.772	0.859	1.55E-54	4	Ogn
7.05E-58	0.48014648	0.907	0.763	1.11E-53	4	Rnh1
2.08E-56	0.52559752	0.369	0.123	3.26E-52	4	Vcam1
2.15E-55	-0.6468279	0.413	0.626	3.37E-51	4	Net1
7.54E-55	0.35297732	0.267	0.065	1.18E-50	4	Phactr3
3.65E-54	0.54746369	0.94	0.851	5.73E-50	4	Tuba1a
4.29E-54	-1.3523055	0.736	0.834	6.73E-50	4	Hspa1a
6.78E-54	0.2739261	0.146	0.017	1.06E-49	4	Sema3e
6.43E-53	-0.5539657	0.63	0.768	1.01E-48	4	Ecm2
1.13E-52	0.44254227	0.635	0.348	1.77E-48	4	Prnp
3.33E-52	-0.553714	0.937	0.959	5.22E-48	4	Cfh
4.56E-52	-0.5997754	1	0.997	7.15E-48	4	Hspa8
2.60E-51	0.39495122	0.504	0.231	4.07E-47	4	Plaur
6.17E-51	0.34852858	0.304	0.092	9.67E-47	4	Dtnb
8.62E-51	0.40914328	0.992	0.963	1.35E-46	4	Anxa5
1.05E-50	-0.623287	0.927	0.951	1.65E-46	4	Nov
2.47E-50	0.42847787	0.999	0.993	3.87E-46	4	Tm4sf1
8.58E-49	-0.5323741	0.47	0.646	1.35E-44	4	Npr1
9.88E-49	-0.404456	0.942	0.929	1.55E-44	4	Pdlim1
2.19E-48	-0.5515519	0.815	0.875	3.43E-44	4	Dnajb4
3.36E-48	-0.3936629	0.973	0.979	5.27E-44	4	Mylk
3.42E-47	0.49582808	0.994	0.984	5.37E-43	4	Fhl1

3.93E-47	0.33272578	0.17	0.028	6.17E-43	4	Fst
6.91E-47	-0.4044431	0.908	0.963	1.08E-42	4	Hcfc1r1
1.59E-46	0.60337488	0.54	0.273	2.49E-42	4	Timp1
1.04E-45	0.76050755	0.782	0.567	1.63E-41	4	Sncg
1.14E-44	-0.4420106	0.835	0.881	1.78E-40	4	Prelp
1.75E-44	0.41446393	0.543	0.288	2.74E-40	4	Mrgprf
1.93E-44	0.53954249	0.398	0.172	3.02E-40	4	Cd44
2.82E-44	0.38530675	0.453	0.209	4.42E-40	4	Aspscr1
2.98E-44	-0.4970597	0.383	0.555	4.67E-40	4	Cdh6
3.79E-44	-0.4441542	0.718	0.792	5.94E-40	4	Gucy1b1
4.68E-44	0.38841404	0.975	0.909	7.34E-40	4	Ltbp1
8.45E-44	0.4475502	0.912	0.802	1.33E-39	4	Col6a2
8.96E-44	-0.3961429	0.868	0.881	1.41E-39	4	Il11ra1
1.04E-43	0.39246583	0.904	0.708	1.63E-39	4	Col5a2
1.30E-43	-0.506418	0.615	0.751	2.04E-39	4	Atp1a2
1.14E-42	0.38721493	0.663	0.404	1.78E-38	4	Sfxn3
1.76E-42	-0.3825468	0.981	0.979	2.76E-38	4	Ddx5
2.29E-42	0.40426874	0.865	0.709	3.59E-38	4	Card19
4.26E-42	0.38832028	0.92	0.789	6.67E-38	4	Cltb
1.96E-41	-0.5313713	0.641	0.757	3.08E-37	4	Limch1
2.10E-41	-0.3751758	0.962	0.951	3.30E-37	4	Zyx
2.82E-41	0.29434654	0.337	0.128	4.43E-37	4	Matn2
9.96E-41	-0.4337162	0.835	0.895	1.56E-36	4	Wfdc1
1.20E-40	0.34668054	0.593	0.337	1.88E-36	4	Phlda3
1.24E-40	-0.5690779	0.523	0.657	1.95E-36	4	Fibin
3.21E-40	0.42576497	0.711	0.479	5.04E-36	4	Plec
1.85E-39	0.39920004	0.773	0.543	2.89E-35	4	Plod2
3.92E-39	0.33229637	0.994	0.957	6.14E-35	4	Hspb7
1.87E-38	0.38929914	0.714	0.47	2.94E-34	4	Cav3
4.45E-38	0.40498306	0.802	0.646	6.98E-34	4	Nme1
7.45E-38	0.34799951	0.46	0.23	1.17E-33	4	Ngef
7.88E-38	0.35294096	0.937	0.814	1.24E-33	4	Ehd2
8.83E-38	0.38690441	0.784	0.564	1.38E-33	4	Adgre5
1.04E-37	0.38741846	0.868	0.705	1.63E-33	4	Utrn
2.04E-37	0.39963577	0.755	0.535	3.20E-33	4	Sulf1
2.06E-37	-0.4465542	0.877	0.916	3.23E-33	4	Fmo2
3.71E-37	0.33214089	0.514	0.276	5.82E-33	4	Sgms2
4.50E-37	-0.6395286	0.96	0.976	7.05E-33	4	Fos
5.40E-37	0.3519651	0.981	0.943	8.46E-33	4	Msn
1.04E-36	-0.3134882	0.964	0.964	1.64E-32	4	Sh3bgrl
1.53E-36	-0.693605	0.614	0.735	2.41E-32	4	Rbp4
1.96E-36	0.38328128	0.551	0.32	3.07E-32	4	Itpripl2
8.16E-36	0.49076313	0.714	0.525	1.28E-31	4	2200002D01Ril
9.68E-36	0.29998249	0.356	0.154	1.52E-31	4	Fam124a
1.04E-35	-0.3881078	0.64	0.72	1.63E-31	4	Ldhb
2.64E-35	-0.5212843	0.821	0.858	4.15E-31	4	Sat1
4.56E-35	0.34653442	0.981	0.958	7.15E-31	4	Arpc1b
8.29E-35	-0.6715996	0.997	0.992	1.30E-30	4	Hspb1
1.27E-34	0.27707988	0.338	0.144	1.99E-30	4	Fam46b
1.66E-34	0.29334649	0.351	0.153	2.60E-30	4	Galnt15
1.67E-34	0.37110127	0.712	0.486	2.61E-30	4	Lamc1

2.03E-34	0.42254598	0.551	0.326	3.18E-30	4	Pcolce2
3.74E-34	0.35333777	0.951	0.846	5.86E-30	4	Col4a1
4.49E-34	0.29355646	0.986	0.968	7.05E-30	4	Rexo2
5.79E-34	0.38076914	0.905	0.788	9.08E-30	4	Col6a1
6.14E-34	-0.4575853	0.311	0.51	9.62E-30	4	Serpini1
7.84E-34	-0.551004	0.889	0.898	1.23E-29	4	Dnaja1
1.42E-33	0.38031333	0.821	0.631	2.23E-29	4	Mgll
1.87E-33	0.34413556	0.767	0.546	2.94E-29	4	Tubb2a
4.82E-33	0.42433595	0.475	0.29	7.56E-29	4	Fdps
5.46E-33	-0.3371568	0.93	0.924	8.55E-29	4	Nexn
8.00E-33	0.33786801	0.992	0.96	1.25E-28	4	Crip2
1.28E-32	0.36029858	0.968	0.936	2.01E-28	4	Pmepa1
1.53E-32	0.29363931	0.715	0.486	2.39E-28	4	Fads3
2.84E-32	-0.4872079	0.203	0.386	4.45E-28	4	Frzb
3.29E-32	-0.2763119	0.987	0.98	5.16E-28	4	Laptm4a
7.56E-32	-0.3792055	0.538	0.661	1.19E-27	4	Tmem38b
3.00E-31	-0.309525	0.801	0.824	4.71E-27	4	Tspan3
6.25E-31	0.2532817	0.99	0.97	9.80E-27	4	Fkbp1a
6.41E-31	-0.3460694	0.992	0.99	1.01E-26	4	Csrp1
7.65E-31	-0.460059	0.187	0.358	1.20E-26	4	Enpp2
8.59E-31	0.36519786	0.847	0.666	1.35E-26	4	Anxa3
1.06E-30	0.33269849	0.929	0.813	1.67E-26	4	Tpm4
1.25E-30	-0.4351926	0.891	0.9	1.96E-26	4	Fgl2
2.31E-30	-0.4067794	0.543	0.667	3.62E-26	4	Pcp4l1
4.77E-30	-0.2963681	1	0.996	7.48E-26	4	Myl9
5.94E-30	0.34232944	0.68	0.469	9.32E-26	4	Vat1
1.02E-29	0.31184152	0.97	0.929	1.60E-25	4	Actn4
1.06E-29	-0.3699426	0.943	0.948	1.66E-25	4	Ncam1
1.21E-29	-0.4243415	0.645	0.705	1.90E-25	4	Tmem110
1.37E-29	-0.4610275	0.495	0.604	2.15E-25	4	Stbd1
1.68E-29	-0.4083735	0.722	0.772	2.64E-25	4	Rhob
2.20E-29	-0.5955613	0.737	0.782	3.44E-25	4	Gadd45g
2.90E-29	0.35817917	0.995	0.973	4.54E-25	4	Timp3
4.24E-29	-0.3434314	0.978	0.975	6.65E-25	4	Ppp1r14a
4.63E-29	-0.3078151	0.934	0.933	7.26E-25	4	Ndufv3
8.32E-29	0.35515862	0.771	0.585	1.30E-24	4	Inhba
9.33E-29	-0.389943	0.772	0.815	1.46E-24	4	Mcam
1.32E-28	0.31095473	0.535	0.324	2.08E-24	4	Asap1
1.47E-28	-0.3049582	0.946	0.958	2.31E-24	4	Ramp1
2.22E-28	-0.350291	0.584	0.65	3.48E-24	4	Dnajc1
3.06E-28	-0.3460058	0.652	0.723	4.80E-24	4	Smarcd3
3.21E-28	0.331779	0.839	0.691	5.04E-24	4	Anxa7
4.10E-28	-0.3318555	0.732	0.768	6.43E-24	4	Aoc3
4.85E-28	-0.3576631	0.988	0.985	7.60E-24	4	Ubc
5.92E-28	0.37758554	0.999	0.998	9.28E-24	4	Crip1
6.77E-28	-0.3504665	0.734	0.795	1.06E-23	4	Efhd1
7.35E-28	-0.3096383	0.858	0.855	1.15E-23	4	Klf9
1.07E-27	0.30969848	0.824	0.646	1.67E-23	4	Tspan2
1.27E-27	0.29425036	0.623	0.411	2.00E-23	4	Col5a1
2.13E-27	-0.3654341	0.742	0.798	3.34E-23	4	Htra3
3.39E-27	0.31305835	0.911	0.783	5.31E-23	4	Col4a2

3.45E-27	0.28379877	0.51	0.305	5.42E-23	4	Lama5
5.45E-27	-0.3565797	0.821	0.874	8.54E-23	4	Gas6
5.57E-27	-0.2800785	0.9	0.907	8.73E-23	4	Tmem59
7.48E-27	-0.4461707	0.99	0.989	1.17E-22	4	Hsp90aa1
8.82E-27	-0.2844027	0.92	0.92	1.38E-22	4	Serinc3
2.03E-26	-0.2765357	0.894	0.894	3.18E-22	4	Atp2a2
2.12E-26	-0.2845573	0.912	0.918	3.33E-22	4	Oxct1
2.97E-26	-0.3311222	0.715	0.759	4.65E-22	4	Olfml2b
4.04E-26	0.25565375	0.548	0.344	6.34E-22	4	Mcm6
4.76E-26	0.27235543	0.666	0.459	7.47E-22	4	Ehd1
4.98E-26	-0.3651035	0.451	0.581	7.81E-22	4	Hspa2
5.10E-26	-0.4457857	0.864	0.882	8.00E-22	4	Id2
5.26E-26	0.29893625	0.39	0.207	8.25E-22	4	Ano1
5.73E-26	0.28220241	0.681	0.477	8.99E-22	4	Ybx3
8.60E-26	0.32912098	0.943	0.863	1.35E-21	4	Tgm2
1.54E-25	0.32651012	0.764	0.578	2.42E-21	4	Phldb2
2.02E-25	-0.4621016	0.312	0.46	3.16E-21	4	Rasl11a
2.04E-25	0.27217402	0.569	0.366	3.19E-21	4	Myof
2.31E-25	0.29923347	0.701	0.504	3.63E-21	4	Ddr2
5.76E-25	-0.2778863	1	0.998	9.04E-21	4	Acta2
6.08E-25	-0.303278	0.913	0.931	9.53E-21	4	Sh3bgr
6.13E-25	-0.3541098	0.424	0.552	9.62E-21	4	Kcnab1
6.89E-25	0.25440291	0.286	0.13	1.08E-20	4	Ldlr
7.24E-25	-0.2704437	0.946	0.939	1.14E-20	4	Aldh2
8.60E-25	-0.3209385	0.212	0.355	1.35E-20	4	Stom
1.01E-24	0.30821368	0.996	0.975	1.58E-20	4	Postn
1.03E-24	0.28162634	0.946	0.853	1.61E-20	4	Arhgdia
1.04E-24	0.25282763	0.245	0.102	1.63E-20	4	Tnfrsf11b
2.24E-24	0.27838173	0.592	0.393	3.51E-20	4	Tspan5
2.29E-24	-0.2614176	0.951	0.958	3.60E-20	4	Smtn
2.89E-24	-0.4557134	0.741	0.767	4.52E-20	4	Jun
3.73E-24	-0.3602535	0.42	0.517	5.85E-20	4	Rasl11b
3.93E-24	-0.2922269	0.946	0.962	6.17E-20	4	Pbxip1
7.93E-24	-0.3361174	0.534	0.583	1.24E-19	4	Tes
8.11E-24	0.30242708	0.781	0.61	1.27E-19	4	Tmem43
8.73E-24	-0.3253044	0.325	0.458	1.37E-19	4	Abcc9
1.10E-23	-0.3289987	0.474	0.574	1.73E-19	4	Arid4b
1.61E-23	0.28375783	0.549	0.356	2.52E-19	4	Itga3
1.68E-23	0.25447683	0.764	0.578	2.63E-19	4	Ppp1r18
1.86E-23	-0.253852	0.06	0.191	2.92E-19	4	Cidea
1.88E-23	-0.2875218	0.804	0.827	2.95E-19	4	Susd5
2.37E-23	0.31036286	0.723	0.543	3.71E-19	4	Odc1
2.46E-23	0.2910897	0.908	0.788	3.85E-19	4	Rock2
5.45E-23	-0.2554337	0.955	0.957	8.55E-19	4	Lhfp
5.88E-23	-0.286091	0.754	0.773	9.22E-19	4	Arl4a
6.88E-23	-0.3214869	0.567	0.611	1.08E-18	4	Zfp36l1
7.92E-23	-0.3177081	0.497	0.6	1.24E-18	4	Smpdl3a
9.03E-23	-0.3735128	0.851	0.897	1.42E-18	4	Ckb
1.31E-22	0.2608764	0.956	0.864	2.05E-18	4	Cdh13
1.46E-22	0.2800547	0.858	0.71	2.29E-18	4	Ctsh
1.87E-22	-0.3261611	0.628	0.714	2.93E-18	4	Mxra8

1.92E-22	-0.3213485	0.648	0.731	3.01E-18	4	Itga9
3.60E-22	0.25668621	0.845	0.689	5.65E-18	4	Myo1c
3.88E-22	-0.3026055	0.641	0.703	6.08E-18	4	Mef2c
5.23E-22	-0.2991654	0.864	0.882	8.21E-18	4	Cbr2
7.56E-22	0.3091029	0.908	0.827	1.19E-17	4	Myadm
9.56E-22	-0.5140276	0.89	0.917	1.50E-17	4	Cyr61
1.23E-21	-0.3032426	0.504	0.571	1.93E-17	4	Scarb2
1.66E-21	0.28284369	0.342	0.184	2.60E-17	4	Adamts5
1.84E-21	-0.2514747	0.996	0.992	2.89E-17	4	Myh11
2.18E-21	-0.3424949	0.422	0.537	3.41E-17	4	Fbxo30
2.27E-21	-0.3471875	0.411	0.555	3.56E-17	4	Emb
2.54E-21	0.26848399	0.707	0.533	3.99E-17	4	.110008P14Ril
2.56E-21	-0.4362849	0.677	0.761	4.02E-17	4	Fbxl22
2.90E-21	0.28195795	0.892	0.784	4.54E-17	4	Capn2
3.12E-21	-0.311201	0.806	0.836	4.89E-17	4	H2afz
3.59E-21	0.27887024	0.794	0.635	5.64E-17	4	Glud1
3.64E-21	0.28043171	0.837	0.689	5.71E-17	4	Sh3bgrl3
4.08E-21	-0.504894	0.94	0.946	6.40E-17	4	Clu
4.11E-21	0.2636806	0.571	0.387	6.44E-17	4	Eml1
4.48E-21	-0.2612146	0.693	0.733	7.03E-17	4	Oaz2
5.47E-21	-0.3498994	0.278	0.425	8.58E-17	4	Nrtn
5.71E-21	-0.2942835	0.246	0.381	8.95E-17	4	Atp1b2
7.99E-21	-0.2648642	0.385	0.45	1.25E-16	4	Acadsb
8.28E-21	-0.3503427	0.356	0.47	1.30E-16	4	Abi3bp
9.11E-21	0.25976493	0.789	0.625	1.43E-16	4	Mprip
1.04E-20	-0.2988344	0.37	0.473	1.64E-16	4	Gstt1
1.06E-20	-0.3037273	0.192	0.31	1.67E-16	4	Map3k7cl
1.12E-20	-0.2942317	0.46	0.545	1.76E-16	4	Asph
1.19E-20	-0.3802608	0.837	0.862	1.86E-16	4	Sparcl1
3.92E-20	0.25631104	0.865	0.733	6.15E-16	4	S100a16
5.35E-20	-0.2622986	0.119	0.24	8.39E-16	4	Atoh8
7.08E-20	-0.2816078	0.092	0.21	1.11E-15	4	Gm13861
8.47E-20	-0.3116051	0.838	0.862	1.33E-15	4	2010111I01Ril
1.08E-19	-0.2772999	0.522	0.59	1.69E-15	4	Sdc2
1.29E-19	-0.2781351	0.668	0.709	2.02E-15	4	Vasn
2.57E-19	0.27872677	0.652	0.484	4.03E-15	4	Srm
2.82E-19	0.3209856	0.102	0.029	4.42E-15	4	Igfbp2
3.09E-19	-0.3916085	0.238	0.356	4.84E-15	4	Pecam1
3.66E-19	-0.3079431	0.44	0.53	5.73E-15	4	Fas
3.99E-19	-0.2839982	0.725	0.778	6.26E-15	4	Map3k20
5.65E-19	-0.2532898	0.965	0.959	8.85E-15	4	Rock1
5.70E-19	-0.3256539	0.916	0.931	8.94E-15	4	Fabp4
5.98E-19	-0.2766863	0.358	0.447	9.38E-15	4	Nfe2l2
6.14E-19	-0.3122113	0.167	0.299	9.64E-15	4	Smim5
7.03E-19	0.27083263	0.736	0.574	1.10E-14	4	Shisa4
7.25E-19	-0.2720163	0.462	0.544	1.14E-14	4	C1qtnf2
9.14E-19	-0.320655	0.26	0.39	1.43E-14	4	Fabp3
1.13E-18	-0.298258	0.31	0.402	1.77E-14	4	Glul
1.42E-18	-0.2667531	0.915	0.916	2.23E-14	4	Nudt4
1.56E-18	-0.3702322	0.79	0.787	2.45E-14	4	Rrad
1.92E-18	-0.3954454	0.145	0.274	3.00E-14	4	Art3

2.03E-18	-0.3811636	0.821	0.874	3.19E-14	4	Actg2
2.81E-18	-0.2602858	0.57	0.631	4.41E-14	4	Idh2
4.25E-18	-0.2763636	0.907	0.899	6.67E-14	4	Ctsl
9.52E-18	-0.3220436	0.407	0.513	1.49E-13	4	Ntf3
9.56E-18	-0.4096167	0.782	0.834	1.50E-13	4	Btg2
1.33E-17	-0.3118231	0.508	0.561	2.09E-13	4	Bambi
1.55E-17	-0.563988	0.424	0.542	2.43E-13	4	Atf3
1.74E-17	-0.2988734	0.253	0.364	2.73E-13	4	Rgs7bp
1.75E-17	-0.2678329	0.359	0.445	2.75E-13	4	Heyl
2.21E-17	0.26197648	0.874	0.755	3.47E-13	4	Tubb4b
3.63E-17	-0.3500744	0.324	0.441	5.69E-13	4	Vwc2
1.21E-16	-0.3664979	0.569	0.596	1.90E-12	4	Cebpd
1.30E-16	-0.3367327	0.79	0.773	2.03E-12	4	Egr1
2.17E-16	-0.2557561	0.981	0.975	3.40E-12	4	Jund
2.92E-16	0.26508723	0.565	0.417	4.58E-12	4	Nhp2
3.23E-16	0.26912564	0.799	0.676	5.06E-12	4	Plpp1
8.18E-16	-0.2583251	0.303	0.396	1.28E-11	4	Ntrk3
8.92E-16	-0.2548481	0.399	0.475	1.40E-11	4	Alcam
1.22E-15	-0.3124842	0.526	0.601	1.91E-11	4	Olfr1033
1.30E-15	-0.2505617	0.321	0.4	2.04E-11	4	Rhobtb1
1.92E-15	-0.2615608	0.644	0.686	3.01E-11	4	.190005I06Ril
2.68E-15	0.30654187	0.149	0.062	4.20E-11	4	Thbs1
1.01E-14	0.29242202	0.856	0.737	1.58E-10	4	Serpine1
1.08E-14	-0.3999262	0.501	0.578	1.69E-10	4	Ier3
1.29E-14	-0.332238	0.896	0.929	2.03E-10	4	Mfap4
4.49E-14	-0.2719271	0.605	0.611	7.04E-10	4	Hsph1
1.12E-13	-0.2892826	0.31	0.397	1.76E-09	4	Haus8
1.31E-13	-0.3622888	0.451	0.472	2.06E-09	4	Dnajb1
1.59E-13	0.3138553	0.946	0.913	2.49E-09	4	Ctgf
5.38E-13	-0.3946562	0.929	0.941	8.44E-09	4	Pam
5.40E-13	-0.4532277	0.303	0.367	8.46E-09	4	Gm12840
1.00E-12	-0.2529292	0.67	0.685	1.57E-08	4	Osr1
1.03E-12	-0.2529926	0.494	0.538	1.62E-08	4	Fam46a
1.09E-12	-0.2653703	0.903	0.909	1.71E-08	4	Mfap5
1.29E-12	-0.4107913	0.311	0.362	2.02E-08	4	Hspa1b
1.55E-12	-0.2986006	0.944	0.93	2.42E-08	4	Lmcd1
3.29E-12	0.25789281	0.749	0.629	5.16E-08	4	Cdo1
3.43E-12	-0.2565721	0.185	0.301	5.37E-08	4	Timp4
6.12E-12	-0.256504	0.291	0.395	9.59E-08	4	Mgst1
8.92E-12	-0.2974969	0.63	0.687	1.40E-07	4	Mylk4
1.28E-11	-0.2738863	0.755	0.766	2.01E-07	4	Ier2
1.71E-11	0.31828042	0.987	0.963	2.69E-07	4	Nupr1
2.08E-11	-0.379155	0.567	0.58	3.26E-07	4	Fosb
4.25E-11	-0.3022184	0.403	0.445	6.66E-07	4	Aspn
6.18E-11	0.25812628	0.772	0.668	9.69E-07	4	Sgk1
1.57E-10	-0.6896613	0.983	0.976	2.46E-06	4	Mt1
1.64E-09	0.38212863	0.902	0.876	2.58E-05	4	Tppp3
5.62E-09	-0.298653	0.398	0.424	8.81E-05	4	Ier5
1.53E-08	0.30625407	0.677	0.621	0.00023954	4	Igfbp4
6.13E-08	-0.7236357	0.86	0.788	0.00096077	4	Mt2
0	2.55536035	0.978	0.581	0	6	Hbegf

1.24E-265	2.41542842	0.863	0.435	1.95E-261	6	Errfi1
1.60E-263	1.91983799	0.969	0.731	2.51E-259	6	Ifrd1
2.04E-252	2.3667604	0.988	0.739	3.19E-248	6	Serpine1
4.03E-248	1.72047698	0.997	0.925	6.31E-244	6	Tnfrsf12a
7.27E-204	1.65582379	0.991	0.989	1.14E-199	6	Hsp90aa1
4.25E-200	2.32827884	0.822	0.182	6.67E-196	6	Emp1
1.54E-196	1.9120438	0.909	0.529	2.41E-192	6	Flnc
7.54E-194	1.54014109	1	0.998	1.18E-189	6	Hspa8
1.53E-192	1.89733197	0.8	0.451	2.40E-188	6	Dnajb1
6.70E-191	1.74350637	0.984	0.892	1.05E-186	6	Dnaja1
3.59E-183	2.56158643	0.944	0.815	5.62E-179	6	Hspa1a
4.19E-177	1.32323202	0.547	0.024	6.57E-173	6	Fosl1
1.90E-171	1.26592689	0.869	0.51	2.98E-167	6	Map2k3
5.05E-164	1.26789447	0.775	0.28	7.91E-160	6	Sgms2
1.08E-160	1.90971941	0.981	0.973	1.69E-156	6	Cryab
1.33E-159	1.53461357	0.741	0.239	2.09E-155	6	Plaur
2.28E-153	1.24186749	0.903	0.614	3.57E-149	6	Tubb6
1.30E-149	1.05926814	0.997	0.988	2.03E-145	6	H3f3b
2.42E-148	1.34472663	0.775	0.366	3.80E-144	6	Maff
3.81E-142	1.85685783	0.828	0.564	5.98E-138	6	Fosb
1.11E-140	1.60145131	0.794	0.4	1.74E-136	6	Ier5
3.73E-140	1.13774163	0.969	0.849	5.84E-136	6	Rtn4
1.68E-139	1.22174402	0.866	0.54	2.64E-135	6	Tuba1c
8.34E-135	1.90490494	0.994	0.993	1.31E-130	6	Hspb1
2.07E-125	1.74438673	0.709	0.336	3.24E-121	6	Hspa1b
9.58E-120	1.26805058	0.625	0.272	1.50E-115	6	Xirp1
6.74E-112	1.23938336	0.972	0.794	1.06E-107	6	Klf6
1.39E-109	1.11695771	0.747	0.497	2.17E-105	6	Bag3
2.95E-107	0.78502589	0.994	0.996	4.62E-103	6	Hsp90ab1
9.46E-107	0.98679719	0.497	0.073	1.48E-102	6	Sphk1
3.10E-104	1.04180286	0.481	0.057	4.87E-100	6	Myc
2.25E-101	1.23331556	0.775	0.601	3.52E-97	6	Hsph1
4.38E-97	1.36880938	0.934	0.897	6.87E-93	6	Fgl2
2.09E-95	1.14118709	0.934	0.764	3.27E-91	6	Itga5
2.87E-93	1.0214222	0.662	0.266	4.50E-89	6	Wsb1
6.21E-91	0.87978766	0.963	0.825	9.74E-87	6	Eif5
1.90E-83	0.78776089	0.422	0.069	2.99E-79	6	Noct
1.45E-81	1.22817911	0.972	0.914	2.28E-77	6	Ctgf
6.96E-80	0.80788997	0.409	0.072	1.09E-75	6	Dusp5
5.09E-79	-0.7859997	0.997	0.996	7.99E-75	6	Myl9
1.13E-77	1.70962244	0.528	0.195	1.76E-73	6	Ptgs2
6.38E-76	0.99371159	0.941	0.824	1.00E-71	6	Arid5b
6.02E-75	1.00279868	0.988	0.924	9.44E-71	6	S100a10
9.82E-75	1.14685526	0.512	0.214	1.54E-70	6	Phlda1
3.61E-73	0.43047628	1	0.999	5.66E-69	6	Eif1
3.67E-71	-0.6668058	1	0.999	5.76E-67	6	Acta2
1.56E-68	0.81992934	0.988	0.972	2.45E-64	6	Lmna
1.93E-67	0.8179159	0.681	0.354	3.02E-63	6	Eif1a
4.02E-67	-0.7902297	0.859	0.972	6.30E-63	6	Lmod1
4.56E-67	0.85057134	0.494	0.181	7.15E-63	6	Rcan1
3.37E-64	1.18740214	0.725	0.358	5.29E-60	6	Cdkn1a

7.76E-64	-0.4230264	1	0.999	1.22E-59	6	Myl6
2.15E-62	0.84378476	0.584	0.29	3.36E-58	6	Csrnp1
4.18E-61	0.77415209	0.444	0.124	6.55E-57	6	Foxs1
5.14E-61	0.90197891	0.947	0.862	8.06E-57	6	Dnajb4
1.87E-60	0.90403091	0.647	0.382	2.93E-56	6	Pde4b
1.92E-60	0.80824634	0.556	0.296	3.01E-56	6	Tsc22d2
2.99E-60	0.80945103	0.631	0.312	4.69E-56	6	Ell2
1.24E-59	0.77742967	0.681	0.383	1.94E-55	6	Nop58
5.91E-58	0.83081588	0.891	0.743	9.27E-54	6	Sqstm1
1.95E-57	0.93138602	0.469	0.185	3.06E-53	6	Cd44
2.06E-57	0.91299728	0.613	0.344	3.23E-53	6	Bdnf
5.59E-54	0.98565688	0.416	0.115	8.76E-50	6	Rgcc
3.00E-53	-0.4719582	1	0.998	4.71E-49	6	Tpm2
7.66E-53	0.80764398	0.784	0.562	1.20E-48	6	Tubb2a
1.92E-52	-0.764158	0.912	0.978	3.00E-48	6	Fos
1.85E-51	0.8623335	0.841	0.702	2.90E-47	6	Lox
3.72E-51	-0.8352625	0.588	0.867	5.84E-47	6	Lims2
3.86E-51	0.50272113	0.225	0.018	6.05E-47	6	Gprc5a
2.24E-50	0.83888818	0.322	0.068	3.52E-46	6	Arc
3.97E-50	-0.6153833	0.988	0.993	6.22E-46	6	Myh11
7.81E-50	-0.6318845	0.916	0.98	1.22E-45	6	Fblim1
3.18E-49	0.75989438	0.988	0.956	4.99E-45	6	Anxa2
5.46E-49	0.60240293	0.262	0.042	8.56E-45	6	Fam107b
1.15E-48	-0.7982998	0.869	0.966	1.80E-44	6	Filip1l
1.25E-48	0.63179702	0.994	0.992	1.95E-44	6	Serpine2
2.94E-48	0.59154053	0.966	0.936	4.60E-44	6	Ube2d3
1.09E-47	0.6972383	0.678	0.394	1.70E-43	6	Azin1
1.28E-47	0.74691402	0.991	0.983	2.01E-43	6	Ftl1
1.77E-47	0.59815768	0.397	0.116	2.78E-43	6	Trib1
4.70E-47	0.86806956	0.231	0.038	7.36E-43	6	Hmox1
7.28E-47	-0.5676662	0.991	0.995	1.14E-42	6	Flna
1.55E-46	0.64168482	0.522	0.285	2.43E-42	6	Rassf1
1.08E-45	0.67855754	0.622	0.339	1.70E-41	6	Gnl3
2.40E-45	-0.696281	0.85	0.958	3.76E-41	6	Zyx
1.87E-44	1.20725672	0.653	0.52	2.94E-40	6	Atf3
2.10E-44	-0.7040397	0.909	0.979	3.29E-40	6	Ppp1r14a
2.97E-44	-0.5976204	0.972	0.981	4.65E-40	6	Gstm1
6.27E-44	-0.4031356	1	1	9.84E-40	6	Fth1
1.34E-43	0.57391206	0.4	0.124	2.11E-39	6	Ets1
3.74E-43	0.51579464	1	0.997	5.86E-39	6	Ptma
6.69E-43	0.58339646	0.919	0.848	1.05E-38	6	Clic1
1.62E-42	0.57414106	0.462	0.181	2.54E-38	6	Dusp10
1.72E-42	0.58248239	0.972	0.942	2.70E-38	6	Eif4a1
1.83E-42	0.7489327	0.831	0.656	2.88E-38	6	Btg1
3.80E-42	0.65789309	0.75	0.598	5.97E-38	6	Zfand5
3.87E-42	0.79611958	0.912	0.753	6.06E-38	6	Sdc4
5.01E-42	0.86016851	0.512	0.225	7.86E-38	6	Pim1
1.05E-41	-0.7334553	0.288	0.665	1.64E-37	6	Npy1r
7.82E-41	-1.0531321	0.469	0.766	1.23E-36	6	Fbxl22
1.42E-40	0.71827348	0.778	0.702	2.23E-36	6	Hspd1
2.30E-40	-0.7482508	0.381	0.731	3.61E-36	6	St5

2.80E-40	-0.9187249	0.672	0.857	4.38E-36	6	Ogn
1.58E-39	1.22837	0.297	0.06	2.48E-35	6	Thbs1
1.92E-39	0.84522029	0.822	0.69	3.02E-35	6	Gm13889
2.03E-39	0.56431279	0.591	0.355	3.18E-35	6	Arf6
7.82E-39	0.63748375	0.884	0.75	1.23E-34	6	Atf4
1.72E-38	0.70705583	0.425	0.231	2.69E-34	6	Glrx
3.33E-38	0.74488432	0.8	0.573	5.22E-34	6	Ppp1r15a
3.50E-38	0.68703067	0.588	0.4	5.50E-34	6	Dnajb9
1.12E-37	-0.7037297	0.169	0.518	1.75E-33	6	Ntf3
1.29E-37	0.57057913	0.647	0.469	2.03E-33	6	Mrps6
1.50E-37	0.57970536	0.478	0.236	2.36E-33	6	Nasp
1.52E-37	0.57940227	0.797	0.605	2.38E-33	6	Coq10b
1.52E-37	-0.6387588	0.966	0.991	2.39E-33	6	Csrp1
1.82E-37	0.59176963	0.409	0.17	2.86E-33	6	Bmp2
2.35E-37	-0.7081171	0.819	0.938	3.68E-33	6	Msrb1
4.99E-37	0.60740777	0.9	0.763	7.83E-33	6	Tubb4b
6.92E-37	-0.6304678	0.391	0.735	1.09E-32	6	Rasl12
1.60E-36	0.56785541	0.537	0.327	2.50E-32	6	Slc2a1
2.10E-36	-0.6515047	0.916	0.979	3.29E-32	6	Col3a1
7.34E-36	0.53539715	0.756	0.659	1.15E-31	6	Top1
9.01E-36	-0.6259258	0.884	0.96	1.41E-31	6	Hcfc1r1
9.10E-36	-0.6381784	0.884	0.962	1.43E-31	6	Rbp1
1.59E-35	-0.7957704	0.319	0.658	2.49E-31	6	Fibin
1.61E-35	-0.6373451	0.625	0.866	2.53E-31	6	Cnn2
2.98E-35	-0.6173237	0.253	0.603	4.67E-31	6	Plin4
9.97E-35	0.69034621	0.95	0.937	1.56E-30	6	Hspe1
1.01E-34	0.59236875	0.694	0.441	1.59E-30	6	Skil
1.44E-34	0.52448463	0.622	0.41	2.25E-30	6	Etf1
1.75E-34	-0.6746094	0.853	0.957	2.74E-30	6	Dusp1
2.63E-34	0.85880111	0.797	0.675	4.13E-30	6	Sgk1
3.36E-34	-0.6778363	0.456	0.739	5.27E-30	6	Ppp1r12b
5.68E-34	-0.7836033	0.319	0.637	8.90E-30	6	[illegible]030013I19Ri[illegible]
7.56E-34	-0.6337606	0.419	0.734	1.19E-29	6	Eva1b
1.05E-33	0.51899238	0.509	0.306	1.64E-29	6	Mafk
1.69E-33	0.27664874	0.147	0.01	2.64E-29	6	Epha2
1.83E-33	-0.647234	0.312	0.645	2.88E-29	6	Gucy1a1
2.91E-33	-0.712928	0.391	0.696	4.56E-29	6	[illegible]190005I06Ri[illegible]
8.43E-33	0.48719947	0.394	0.185	1.32E-28	6	Mpp5
4.00E-32	0.53301705	0.519	0.306	6.27E-28	6	Txnrd1
2.85E-31	0.56728516	0.35	0.121	4.46E-27	6	Gadd45a
2.88E-31	0.48686393	0.466	0.257	4.52E-27	6	Foxc2
8.07E-31	0.43068429	0.991	0.99	1.27E-26	6	Gapdh
1.00E-30	0.521588	0.312	0.111	1.57E-26	6	Arl4d
1.57E-30	0.54261447	0.431	0.22	2.46E-26	6	Chka
1.59E-30	0.35947339	0.206	0.04	2.49E-26	6	Clcf1
7.05E-30	0.41218991	0.291	0.097	1.11E-25	6	Rnf19b
7.52E-30	0.45949381	0.306	0.091	1.18E-25	6	Rnd1
1.20E-29	-0.8441358	0.438	0.693	1.88E-25	6	Mylk4
1.96E-29	0.39882493	0.997	0.992	3.08E-25	6	Cd63
2.03E-29	0.4822641	0.481	0.301	3.19E-25	6	Ipo7
3.02E-29	0.44455924	0.319	0.114	4.73E-25	6	Pvr

4.20E-29	-0.706922	0.8	0.917	6.58E-25	6	Fmo2
7.28E-29	0.52126138	0.288	0.125	1.14E-24	6	Mlf1
1.84E-28	0.47365834	0.775	0.666	2.88E-24	6	Tra2b
2.94E-28	0.48500012	0.453	0.225	4.61E-24	6	Il1r1
3.99E-28	-0.4072492	0.988	0.995	6.25E-24	6	Tpm1
1.30E-27	0.54800258	0.975	0.956	2.04E-23	6	Hspa5
1.32E-27	0.80738297	0.159	0.023	2.07E-23	6	Tnfaip6
1.84E-27	-0.5329496	0.122	0.405	2.89E-23	6	Rhobtb1
2.23E-27	-0.4903506	0.122	0.401	3.49E-23	6	Chst12
2.33E-27	-0.493131	0.941	0.979	3.65E-23	6	Tns1
3.54E-27	-0.4088913	0.922	0.969	5.56E-23	6	Bri3
4.62E-27	0.7537543	0.981	0.965	7.24E-23	6	Nupr1
4.91E-27	0.48091139	0.672	0.486	7.70E-23	6	Bzw1
6.92E-27	-0.525021	0.634	0.859	1.08E-22	6	Adcy5
7.60E-27	0.43235372	0.347	0.206	1.19E-22	6	Slbp
1.33E-26	0.43143841	0.309	0.146	2.09E-22	6	Dusp14
1.87E-26	-0.4381471	0.059	0.309	2.93E-22	6	Klf15
3.39E-26	0.41815472	0.981	0.973	5.32E-22	6	Emp3
5.57E-26	0.43702785	0.412	0.229	8.73E-22	6	Fam214b
6.78E-26	0.4526113	0.491	0.322	1.06E-21	6	Uap1
6.86E-26	0.46640106	0.588	0.372	1.08E-21	6	Tpm3
6.98E-26	0.65298509	0.659	0.481	1.09E-21	6	Gja1
7.13E-26	0.52871783	0.512	0.319	1.12E-21	6	Fosl2
8.87E-26	0.39948295	0.194	0.05	1.39E-21	6	Frat2
1.45E-25	-0.5580717	0.406	0.677	2.27E-21	6	Mrvi1
2.24E-25	0.4824632	0.603	0.42	3.52E-21	6	Ube2f
3.68E-25	-0.4410242	0.953	0.98	5.77E-21	6	Mylk
3.79E-25	-0.8009169	0.653	0.832	5.95E-21	6	Nr4a1
4.01E-25	-0.4689192	0.403	0.698	6.29E-21	6	Osr1
4.94E-25	0.47209444	0.419	0.241	7.75E-21	6	Tmem120a
5.14E-25	0.67798472	0.45	0.3	8.05E-21	6	Socs3
5.91E-25	0.50190009	0.684	0.529	9.27E-21	6	Srsf7
7.21E-25	0.48134192	0.356	0.184	1.13E-20	6	Uaca
8.94E-25	0.63254014	0.697	0.603	1.40E-20	6	Inhba
1.30E-24	0.47893122	0.637	0.518	2.04E-20	6	Pxdc1
2.19E-24	0.40368229	0.359	0.221	3.44E-20	6	Arl13b
2.67E-24	0.46940819	0.688	0.51	4.18E-20	6	Eif4e
3.08E-24	0.51605297	0.878	0.829	4.83E-20	6	H2afz
3.16E-24	-0.5359546	0.678	0.843	4.95E-20	6	Rras
3.16E-24	0.30764677	0.194	0.053	4.96E-20	6	Homer1
3.94E-24	0.49130174	0.903	0.845	6.17E-20	6	Ddx3x
4.10E-24	0.38631085	0.756	0.75	6.42E-20	6	S100a16
4.24E-24	0.4052888	0.938	0.906	6.66E-20	6	Ran
6.01E-24	0.5674563	0.431	0.205	9.42E-20	6	Acan
8.61E-24	0.45018304	0.303	0.111	1.35E-19	6	Osmr
8.66E-24	0.45435328	0.475	0.309	1.36E-19	6	Sertad2
8.76E-24	0.43851444	0.381	0.166	1.37E-19	6	Hk2
1.22E-23	0.39010009	0.312	0.113	1.92E-19	6	Sbno2
1.40E-23	0.46355095	0.991	0.979	2.19E-19	6	Ddx5
1.40E-23	0.40859954	0.284	0.104	2.20E-19	6	Il13ra1
1.44E-23	0.42108153	0.312	0.139	2.25E-19	6	Dnaja4

1.51E-23	-0.5149033	0.356	0.632	2.37E-19	6	Ypel3
1.54E-23	-0.5925115	0.884	0.952	2.42E-19	6	Nov
1.89E-23	-0.5002139	0.912	0.973	2.97E-19	6	Id3
2.27E-23	0.44300071	0.409	0.215	3.56E-19	6	Cttnbp2nl
3.26E-23	0.50541404	0.706	0.55	5.12E-19	6	Nr1d1
3.87E-23	-0.488614	0.559	0.801	6.07E-19	6	Cdc42ep3
4.79E-23	-0.4890978	0.428	0.68	7.51E-19	6	Coa3
5.99E-23	0.4730508	0.75	0.598	9.40E-19	6	Sertad1
8.41E-23	0.42508715	0.947	0.914	1.32E-18	6	Ldha
1.23E-22	0.47701281	0.616	0.497	1.93E-18	6	Mcl1
1.50E-22	-0.3928729	0.884	0.944	2.35E-18	6	Ndufs5
1.64E-22	-0.7439917	0.725	0.842	2.58E-18	6	Sost
2.06E-22	0.36437702	0.272	0.092	3.24E-18	6	Baz1a
2.63E-22	0.42365549	0.925	0.911	4.12E-18	6	Fermt2
3.04E-22	-0.4707654	0.65	0.835	4.77E-18	6	Polr2f
3.35E-22	0.51008441	0.997	0.994	5.25E-18	6	Tm4sf1
4.61E-22	0.44718089	0.853	0.77	7.22E-18	6	Hnrnpu
5.04E-22	-0.5196888	0.55	0.755	7.90E-18	6	Emc8
7.39E-22	0.6991539	0.544	0.293	1.16E-17	6	Timp1
1.21E-21	0.63106363	0.903	0.85	1.90E-17	6	Sat1
1.59E-21	0.408949	0.994	0.989	2.50E-17	6	Serpinh1
2.45E-21	0.40975732	0.384	0.209	3.85E-17	6	Med13
2.75E-21	0.40431337	0.366	0.181	4.31E-17	6	Tcp11l2
3.00E-21	0.41675687	0.356	0.199	4.70E-17	6	Slc35e4
3.11E-21	-0.4431498	0.731	0.886	4.87E-17	6	Cyb5a
3.79E-21	0.44579617	0.525	0.342	5.94E-17	6	Nop56
3.80E-21	-0.5487657	0.469	0.712	5.96E-17	6	Optc
3.83E-21	0.42130241	0.394	0.209	6.00E-17	6	Midn
4.05E-21	0.5274515	0.897	0.869	6.35E-17	6	Tmsb10
4.94E-21	-0.5088924	0.525	0.756	7.75E-17	6	Nox4
6.28E-21	-0.5261988	0.806	0.914	9.85E-17	6	Mfap5
6.98E-21	-0.4459052	0.856	0.944	1.09E-16	6	Slmap
8.43E-21	-0.4548209	0.2	0.454	1.32E-16	6	Ak3
1.31E-20	-0.4697829	0.172	0.425	2.06E-16	6	Asb2
2.41E-20	-1.0181337	0.238	0.47	3.78E-16	6	Rgs2
2.45E-20	0.41351396	0.494	0.338	3.84E-16	6	Ssfa2
2.52E-20	0.42248317	0.522	0.343	3.96E-16	6	Stat3
3.71E-20	0.41139711	0.35	0.18	5.82E-16	6	Kdm6b
3.95E-20	-0.5319664	0.734	0.89	6.19E-16	6	Cd200
6.55E-20	0.40753938	0.903	0.833	1.03E-15	6	Arf4
6.69E-20	0.46852844	0.456	0.271	1.05E-15	6	Ddx21
6.98E-20	0.45039386	0.678	0.56	1.09E-15	6	Odc1
7.67E-20	0.48860594	0.406	0.254	1.20E-15	6	Nfil3
1.14E-19	0.45228964	0.562	0.354	1.78E-15	6	Smad7
1.49E-19	0.4059741	0.831	0.758	2.33E-15	6	Ppp1r2
1.51E-19	0.45763465	0.722	0.66	2.37E-15	6	Hspb8
1.63E-19	-0.4064644	0.8	0.907	2.55E-15	6	Tgfb1i1
1.66E-19	0.42145891	0.969	0.953	2.61E-15	6	Lrrfip1
2.08E-19	0.4246131	0.334	0.178	3.26E-15	6	F3
2.74E-19	-0.3820198	0.834	0.937	4.29E-15	6	Pdlim3
3.13E-19	0.53825991	0.519	0.399	4.91E-15	6	Tob1

3.46E-19	0.40573556	0.95	0.914	5.43E-15	6	Ncl
3.82E-19	0.30889458	0.219	0.076	5.99E-15	6	Gfod1
4.45E-19	0.39254058	0.212	0.074	6.98E-15	6	Ereg
4.50E-19	-0.3590347	0.972	0.989	7.05E-15	6	Itga8
4.98E-19	-0.375589	0.884	0.95	7.81E-15	6	Ndufa7
5.89E-19	0.40814825	0.975	0.976	9.23E-15	6	Jund
5.90E-19	0.46225795	0.522	0.383	9.25E-15	6	Peli1
6.02E-19	0.48321634	0.725	0.582	9.44E-15	6	Nes
8.26E-19	0.35882079	0.297	0.143	1.30E-14	6	Tuba4a
8.43E-19	0.74507735	0.803	0.762	1.32E-14	6	Jun
9.32E-19	-0.4585776	0.209	0.446	1.46E-14	6	Hey1
9.60E-19	-0.2859643	0.997	0.997	1.51E-14	6	Dstn
1.20E-18	0.465197	0.812	0.723	1.88E-14	6	Aqp1
1.73E-18	-0.4809039	0.703	0.863	2.72E-14	6	Synpo2
2.19E-18	0.52542678	0.641	0.582	3.44E-14	6	Tgfb2
3.64E-18	-0.4221821	0.884	0.944	5.71E-14	6	Cped1
3.78E-18	-0.4467457	0.766	0.894	5.92E-14	6	Wfdc1
4.79E-18	0.38294561	0.5	0.331	7.52E-14	6	Yrdc
5.31E-18	0.34669378	0.347	0.171	8.32E-14	6	Prkca
5.41E-18	0.56417343	0.812	0.656	8.49E-14	6	Lgals3
7.65E-18	-0.4116704	0.772	0.884	1.20E-13	6	Ndufb3
8.53E-18	0.48987716	0.95	0.882	1.34E-13	6	Prss23
9.02E-18	-0.3111959	0.963	0.989	1.41E-13	6	Selenow
1.00E-17	0.36427895	0.731	0.698	1.57E-13	6	Ubxn4
1.01E-17	-0.3608745	0.869	0.948	1.58E-13	6	Ilk
1.01E-17	0.31799252	0.316	0.177	1.58E-13	6	Rrs1
1.37E-17	0.42238047	0.997	0.991	2.15E-13	6	Malat1
1.60E-17	0.32034319	0.403	0.298	2.51E-13	6	Kcmf1
2.11E-17	-0.3823505	0.825	0.928	3.31E-13	6	Ndufb7
2.70E-17	-0.4653614	0.528	0.724	4.24E-13	6	Smarcd3
2.86E-17	0.30967514	0.247	0.101	4.49E-13	6	Serpinb8
3.02E-17	0.65381076	0.8	0.774	4.74E-13	6	Egr1
3.16E-17	0.38232565	0.853	0.777	4.95E-13	6	Rnh1
3.54E-17	0.41230084	0.762	0.661	5.54E-13	6	Foxp1
4.25E-17	0.5656173	0.694	0.493	6.67E-13	6	Fxyd5
4.54E-17	0.36771199	0.322	0.208	7.11E-13	6	Thbs2
4.70E-17	0.43932134	0.15	0.053	7.37E-13	6	Tnfsf9
5.46E-17	0.5240401	0.838	0.72	8.56E-13	6	Gadd45b
6.41E-17	-0.6985347	0.544	0.729	1.00E-12	6	Rbp4
6.57E-17	-0.4156903	0.216	0.445	1.03E-12	6	Bmyc
7.03E-17	-0.3351672	0.844	0.934	1.10E-12	6	Park7
7.31E-17	-0.296401	0.984	0.989	1.15E-12	6	Cnn1
7.43E-17	0.29563683	0.2	0.072	1.17E-12	6	Slc16a10
7.86E-17	-0.4555432	0.425	0.649	1.23E-12	6	Marveld1
8.22E-17	-0.3764912	0.119	0.328	1.29E-12	6	Tbxa2r
8.70E-17	0.36510969	0.906	0.853	1.36E-12	6	Pabpc1
1.18E-16	0.26463837	0.212	0.066	1.85E-12	6	Tgif1
1.19E-16	0.35270528	0.828	0.795	1.86E-12	6	Eif2s2
1.37E-16	0.37937878	0.447	0.327	2.15E-12	6	Jpt1
1.45E-16	-0.4030876	0.844	0.934	2.28E-12	6	Sh3bgr
1.47E-16	-0.2753929	1	0.998	2.30E-12	6	Crip1

1.73E-16	0.29739996	0.278	0.123	2.71E-12	6	Ctdp1
1.94E-16	-0.440002	0.312	0.548	3.05E-12	6	Lrrc17
1.97E-16	0.29099458	0.203	0.078	3.09E-12	6	Abl2
2.12E-16	-0.3650163	0.853	0.924	3.33E-12	6	Ndufb10
2.62E-16	-0.3979615	0.556	0.764	4.12E-12	6	Dctn6
3.05E-16	-0.4136741	0.312	0.546	4.78E-12	6	C1qtnf2
3.90E-16	0.3046237	0.166	0.049	6.11E-12	6	Egr3
3.93E-16	0.32602673	0.334	0.167	6.17E-12	6	Bcar1
4.97E-16	-0.4268003	0.756	0.886	7.80E-12	6	Il11ra1
5.77E-16	0.43329388	0.472	0.355	9.05E-12	6	Otud1
6.26E-16	0.4535714	0.581	0.377	9.82E-12	6	Cd109
6.29E-16	0.34018111	0.281	0.182	9.86E-12	6	Srxn1
7.28E-16	0.28531476	0.269	0.115	1.14E-11	6	Slc7a1
7.35E-16	0.31154157	0.203	0.083	1.15E-11	6	Tiparp
8.49E-16	-0.3258822	0.906	0.948	1.33E-11	6	Atp5g2
8.78E-16	0.41437841	0.672	0.579	1.38E-11	6	Por
8.84E-16	-0.2530808	0.991	0.99	1.39E-11	6	Slc25a4
9.40E-16	-0.3454067	0.359	0.596	1.47E-11	6	Krcc1
1.41E-15	0.40200952	0.478	0.302	2.22E-11	6	Esam
1.70E-15	-0.3784519	0.159	0.368	2.66E-11	6	Snx18
1.70E-15	-0.3060464	0.094	0.282	2.67E-11	6	Smim20
1.80E-15	-0.3441868	0.225	0.449	2.82E-11	6	Mrpl55
1.88E-15	0.512213	0.628	0.579	2.95E-11	6	Gem
2.13E-15	-0.4044786	0.103	0.292	3.34E-11	6	Smim5
2.15E-15	-0.384276	0.478	0.696	3.37E-11	6	Pkp4
2.29E-15	-0.4115147	0.934	0.962	3.59E-11	6	Rock1
2.65E-15	-0.3906781	0.525	0.72	4.15E-11	6	Ldhb
2.81E-15	0.35275053	0.591	0.511	4.41E-11	6	Fads3
3.02E-15	-0.366335	0.456	0.673	4.73E-11	6	Mrpl18
3.29E-15	0.34905939	0.716	0.6	5.16E-11	6	Rap1b
3.39E-15	0.3042925	0.316	0.168	5.31E-11	6	Ncs1
3.76E-15	0.29112607	0.266	0.109	5.89E-11	6	Mapk6
4.40E-15	0.56410578	0.656	0.563	6.89E-11	6	Ier3
4.46E-15	0.34522558	0.909	0.825	7.00E-11	6	Rac1
5.17E-15	0.39556519	0.275	0.13	8.11E-11	6	Nr4a3
5.28E-15	-0.3451714	0.934	0.971	8.27E-11	6	Ppp1r12a
5.29E-15	-0.3564719	0.844	0.929	8.30E-11	6	Nexn
5.56E-15	0.39311437	0.747	0.618	8.72E-11	6	Tmbim1
5.67E-15	-0.2925685	0.1	0.29	8.90E-11	6	Lsm10
5.71E-15	-0.3452504	0.906	0.95	8.95E-11	6	Bcam
6.32E-15	0.35561525	0.388	0.217	9.91E-11	6	Taf1d
6.42E-15	0.35312412	0.469	0.309	1.01E-10	6	Slc6a6
7.33E-15	-0.384763	0.322	0.542	1.15E-10	6	Snapc5
8.59E-15	0.37617092	0.688	0.55	1.35E-10	6	Akap13
9.64E-15	-0.4042356	0.278	0.498	1.51E-10	6	Fam13c
1.02E-14	-0.3475948	0.688	0.832	1.60E-10	6	Ndufs7
1.07E-14	-0.2562358	0.972	0.988	1.67E-10	6	Pls3
1.22E-14	-0.3292705	0.378	0.606	1.91E-10	6	Polr2j
1.64E-14	0.36263844	0.472	0.315	2.58E-10	6	Csnk1d
1.82E-14	-0.3834979	0.434	0.64	2.85E-10	6	Mrpl14
1.96E-14	0.38182003	0.609	0.426	3.08E-10	6	P4ha1

1.99E-14	-0.362893	0.781	0.895	3.13E-10	6	Iscu
2.26E-14	0.29876525	0.316	0.184	3.54E-10	6	Ppp1r15b
2.32E-14	-0.3595832	0.262	0.477	3.65E-10	6	Pop5
2.42E-14	-0.2938097	0.919	0.968	3.80E-10	6	Uqcr11
2.45E-14	-0.3662842	0.291	0.507	3.84E-10	6	Pigyl
2.82E-14	-0.3630003	0.381	0.601	4.42E-10	6	Islr
2.89E-14	0.30252119	0.256	0.12	4.53E-10	6	Ndrg1
3.15E-14	0.27999259	0.197	0.076	4.94E-10	6	B4galt5
3.33E-14	-0.3460668	0.244	0.461	5.23E-10	6	Tpgs1
3.67E-14	-0.3376606	0.7	0.852	5.75E-10	6	Ndufv2
3.72E-14	0.31957516	0.828	0.804	5.83E-10	6	Cltb
4.67E-14	0.27882855	0.256	0.118	7.32E-10	6	Gm47283
4.74E-14	0.27234576	0.281	0.134	7.43E-10	6	Nup62
4.80E-14	-0.3553423	0.819	0.909	7.52E-10	6	Rsu1
4.81E-14	-0.3282684	0.959	0.977	7.54E-10	6	Rbpms
5.03E-14	-0.2914605	0.922	0.955	7.89E-10	6	Atp5d
5.12E-14	0.35945168	0.478	0.395	8.02E-10	6	Creb5
5.86E-14	-0.3508944	0.656	0.812	9.20E-10	6	Ndufa8
6.16E-14	-0.2695748	0.034	0.176	9.65E-10	6	Trp53inp1
6.23E-14	-0.3332409	0.253	0.469	9.76E-10	6	Mrpl58
7.35E-14	0.3460162	0.447	0.342	1.15E-09	6	Spcs3
7.51E-14	-0.3423378	0.903	0.96	1.18E-09	6	Smtn
7.70E-14	-0.2543287	0.988	0.991	1.21E-09	6	Actn1
8.02E-14	-0.4326681	0.509	0.708	1.26E-09	6	Tmem110
8.08E-14	-0.2551805	0.975	0.976	1.27E-09	6	Nenf
8.12E-14	-0.4805645	0.409	0.609	1.27E-09	6	Net1
8.23E-14	-0.3628895	0.253	0.45	1.29E-09	6	Sspn
8.37E-14	0.31434773	0.303	0.177	1.31E-09	6	Jmjd1c
9.17E-14	-0.2865961	0.906	0.956	1.44E-09	6	Ndufb9
9.35E-14	-0.3567833	0.447	0.659	1.47E-09	6	Eif4ebp1
1.07E-13	-0.358062	0.606	0.76	1.68E-09	6	Vamp8
1.10E-13	0.31075181	0.656	0.56	1.73E-09	6	Fam104a
1.17E-13	-0.2892689	0.362	0.582	1.83E-09	6	Rgs4
1.37E-13	-0.383259	0.478	0.665	2.15E-09	6	Etfb
1.40E-13	-0.3535182	0.247	0.456	2.20E-09	6	Zfyve21
1.47E-13	-0.3824131	0.778	0.873	2.31E-09	6	Gas6
1.69E-13	0.32530543	0.669	0.595	2.64E-09	6	Dlgap4
1.69E-13	-0.2927813	0.034	0.174	2.65E-09	6	Wisp2
1.72E-13	-0.3761052	0.431	0.642	2.69E-09	6	Ech1
1.72E-13	0.3354238	0.903	0.91	2.70E-09	6	[illegible]930523C07Ri[illegible]
1.85E-13	-0.3121217	0.212	0.419	2.90E-09	6	Zmat5
1.90E-13	0.34866293	0.819	0.72	2.98E-09	6	Hnrnpa1
1.92E-13	-0.5090256	0.688	0.835	3.02E-09	6	Btg2
1.96E-13	-0.2576693	0.956	0.982	3.07E-09	6	Atp5j2
2.09E-13	-0.3381768	0.556	0.751	3.28E-09	6	Ift43
2.27E-13	-0.2846915	0.912	0.948	3.56E-09	6	Nedd8
2.41E-13	-0.3448078	0.172	0.366	3.78E-09	6	Fzd2
2.63E-13	-0.2646352	0.075	0.24	4.13E-09	6	Tcta
2.98E-13	-0.3640042	0.75	0.872	4.67E-09	6	Ndrg2
3.06E-13	-0.3180857	0.191	0.391	4.79E-09	6	Eci1
3.77E-13	0.56498826	0.944	0.931	5.92E-09	6	Lmcd1

3.85E-13	0.32081616	0.825	0.761	6.04E-09	6	Chmp4b
3.85E-13	-0.3137222	0.919	0.966	6.04E-09	6	Sh3bgrl
4.02E-13	-0.3516003	0.388	0.588	6.31E-09	6	Maf1
4.19E-13	0.30698634	0.544	0.44	6.57E-09	6	Fbl
4.48E-13	-0.3381823	0.475	0.676	7.02E-09	6	Sf3b5
4.55E-13	-0.3882534	0.791	0.885	7.13E-09	6	Cbr2
4.88E-13	0.30982007	0.412	0.26	7.65E-09	6	Jmjd6
4.88E-13	-0.3027435	0.256	0.466	7.66E-09	6	Echs1
4.90E-13	0.29343156	0.356	0.22	7.68E-09	6	Bcl10
5.07E-13	-0.2978434	0.131	0.316	7.94E-09	6	Cir1
6.08E-13	-0.3296946	0.369	0.58	9.53E-09	6	Pnkd
9.97E-13	-0.2849474	0.981	0.981	1.56E-08	6	Map1b
1.07E-12	0.30123439	0.309	0.183	1.68E-08	6	Rc3h1
1.16E-12	0.29377723	0.681	0.669	1.81E-08	6	Cacybp
1.18E-12	0.294787	0.938	0.941	1.85E-08	6	Slc25a5
1.20E-12	-0.2956543	0.103	0.274	1.88E-08	6	Adam33
1.22E-12	-0.5679871	0.306	0.463	1.92E-08	6	Dbp
1.25E-12	0.32305006	0.441	0.314	1.96E-08	6	Elf1
1.44E-12	0.32479869	0.553	0.44	2.25E-08	6	Fam129b
1.47E-12	0.33858927	0.369	0.245	2.30E-08	6	Msmo1
1.63E-12	-0.3189254	0.709	0.837	2.56E-08	6	Med28
1.64E-12	-0.3013709	0.791	0.895	2.57E-08	6	Atp5o.1
1.65E-12	-0.2889051	0.812	0.911	2.59E-08	6	Atp6v1g1
1.68E-12	-0.5017032	0.322	0.508	2.63E-08	6	Nr4a2
1.77E-12	-0.3347944	0.291	0.496	2.78E-08	6	Mxd4
1.79E-12	0.25402892	0.172	0.055	2.81E-08	6	Marcksl1
1.95E-12	-0.3465145	0.369	0.574	3.06E-08	6	Ttc3
2.04E-12	-0.2870969	0.128	0.305	3.19E-08	6	Gipc1
2.12E-12	0.35858135	0.869	0.811	3.33E-08	6	Col15a1
2.53E-12	-0.3091469	0.412	0.616	3.96E-08	6	Nsmce4a
2.57E-12	0.33212834	0.863	0.781	4.03E-08	6	Hnrnpab
2.84E-12	0.28747409	0.294	0.153	4.46E-08	6	Litaf
3.17E-12	-0.311288	0.331	0.538	4.97E-08	6	Lsm7
3.27E-12	0.39105508	0.462	0.326	5.13E-08	6	Cited2
3.85E-12	-0.3177655	0.309	0.513	6.03E-08	6	Mrpl27
3.95E-12	-0.2941131	0.847	0.935	6.20E-08	6	Rbx1
4.14E-12	0.55222836	0.803	0.797	6.49E-08	6	Mt2
4.18E-12	0.34333997	0.581	0.486	6.56E-08	6	Ccnl1
4.21E-12	-0.3267197	0.675	0.827	6.60E-08	6	Mrps21
4.26E-12	0.34201788	0.884	0.797	6.68E-08	6	Iqgap1
4.29E-12	0.27399708	0.984	0.98	6.73E-08	6	Col18a1
4.43E-12	0.3249478	0.391	0.25	6.95E-08	6	Zfp131
4.44E-12	-0.3000746	0.934	0.966	6.97E-08	6	Rarres2
4.62E-12	0.27405709	0.328	0.202	7.24E-08	6	Stk40
4.81E-12	0.30238819	0.722	0.635	7.54E-08	6	Snrpd1
5.08E-12	0.26240036	0.256	0.149	7.96E-08	6	Klhl21
5.21E-12	-0.3324901	0.541	0.731	8.16E-08	6	Dusp3
5.34E-12	-0.3183294	0.55	0.729	8.38E-08	6	Ndufs3
5.40E-12	-0.3010249	0.216	0.408	8.47E-08	6	Timm17b
5.85E-12	-0.3362261	0.906	0.956	9.18E-08	6	Hspb6
6.09E-12	-0.3437221	0.744	0.861	9.55E-08	6	Cox7a2l

6.32E-12	0.29112376	0.981	0.986	9.91E-08	6	Txn1
6.85E-12	0.26019769	0.988	0.984	1.07E-07	6	Mfge8
7.34E-12	-0.3199853	0.231	0.422	1.15E-07	6	Rilpl1
7.86E-12	-0.2663195	0.878	0.952	1.23E-07	6	Swi5
8.12E-12	0.25660148	1	0.997	1.27E-07	6	Bgn
8.13E-12	0.29930742	0.716	0.707	1.28E-07	6	Fstl3
8.48E-12	0.29653026	0.425	0.285	1.33E-07	6	Nolc1
8.58E-12	-0.3325737	0.45	0.641	1.35E-07	6	Siva1
8.64E-12	0.51104926	1	0.999	1.35E-07	6	Ubb
8.70E-12	0.32742065	0.622	0.487	1.36E-07	6	Ckap4
9.29E-12	-0.4790728	0.866	0.893	1.46E-07	6	Ckb
9.93E-12	0.32041407	0.516	0.379	1.56E-07	6	Cdv3
9.98E-12	-0.3316271	0.647	0.787	1.57E-07	6	Sdhb
1.14E-11	-0.3127282	0.787	0.883	1.79E-07	6	Anapc11
1.20E-11	-0.3322826	0.678	0.824	1.89E-07	6	Kcnmb1
1.30E-11	0.3055569	0.831	0.767	2.03E-07	6	Ube2s
1.38E-11	0.28721361	0.453	0.324	2.16E-07	6	Chic2
1.42E-11	-0.3209007	0.434	0.626	2.22E-07	6	Tmem147
1.43E-11	-0.3224128	0.303	0.503	2.24E-07	6	Ptp4a3
1.46E-11	-0.309487	0.234	0.427	2.28E-07	6	Cirbp
1.57E-11	-0.2516791	0.081	0.233	2.46E-07	6	Atoh8
1.71E-11	-0.3183056	0.575	0.744	2.69E-07	6	Ap2s1
1.80E-11	0.32403683	0.7	0.628	2.83E-07	6	Tmem43
1.90E-11	-0.5002607	0.109	0.255	2.98E-07	6	Fmo3
1.96E-11	-0.3092788	0.537	0.718	3.07E-07	6	Ndufs8
1.99E-11	0.30400597	0.359	0.241	3.12E-07	6	Pim3
2.00E-11	-0.2764122	0.125	0.293	3.14E-07	6	Rab3a
2.03E-11	0.38691245	0.516	0.388	3.18E-07	6	Atp1b1
2.10E-11	0.29195155	0.881	0.828	3.29E-07	6	Vapa
2.14E-11	0.36899942	0.481	0.353	3.35E-07	6	Fgf2
2.16E-11	-0.3240798	0.238	0.429	3.39E-07	6	Cavin2
2.21E-11	-0.4331451	0.256	0.436	3.47E-07	6	Vwc2
2.23E-11	-0.2778904	0.119	0.283	3.50E-07	6	Oard1
2.26E-11	0.36060414	0.919	0.899	3.55E-07	6	Ctsl
2.35E-11	0.26956491	0.353	0.205	3.69E-07	6	Nmd3
2.44E-11	-0.3225723	0.534	0.71	3.82E-07	6	Lamtor4
2.50E-11	0.30244237	0.963	0.947	3.92E-07	6	Msn
2.53E-11	-0.2747767	0.128	0.297	3.96E-07	6	Dguok
2.72E-11	0.31877745	0.753	0.683	4.27E-07	6	Srsf2
2.84E-11	-0.4523445	0.425	0.599	4.45E-07	6	Stbd1
2.88E-11	0.34829089	0.469	0.342	4.51E-07	6	Nedd9
2.98E-11	-0.433387	0.181	0.35	4.68E-07	6	Pecam1
3.02E-11	-0.3226735	0.725	0.847	4.73E-07	6	Aes
3.13E-11	-0.315765	0.35	0.546	4.92E-07	6	Kcnab1
3.16E-11	0.29923041	0.659	0.607	4.95E-07	6	Brd2
3.66E-11	-0.3023134	0.741	0.859	5.74E-07	6	Ndufc2
3.75E-11	-0.3271333	0.428	0.616	5.88E-07	6	Speg
3.94E-11	0.30811277	0.697	0.618	6.18E-07	6	Psmd11
3.99E-11	-0.2654985	0.894	0.946	6.26E-07	6	D8Ertd738e
4.03E-11	0.25223532	0.131	0.057	6.32E-07	6	Casp4
4.39E-11	0.29038884	0.372	0.215	6.88E-07	6	Ets2

4.51E-11	-0.3417503	0.516	0.681	7.07E-07	6	Tsn
4.54E-11	-0.331233	0.637	0.778	7.12E-07	6	Fis1
4.69E-11	0.26527378	0.375	0.249	7.36E-07	6	Timm10
4.78E-11	-0.2540392	0.119	0.281	7.49E-07	6	Psmg2
4.89E-11	0.30546078	0.537	0.409	7.67E-07	6	Nfat5
5.03E-11	-0.2699818	0.838	0.907	7.89E-07	6	Ndufs6
5.33E-11	-0.2990408	0.787	0.884	8.36E-07	6	Snrpd2
5.52E-11	-0.2575716	0.766	0.887	8.65E-07	6	Srp14
5.62E-11	-0.2931023	0.191	0.371	8.81E-07	6	Dhrs3
5.78E-11	0.29596389	0.488	0.349	9.06E-07	6	Ube2g1
5.87E-11	-0.2983864	0.334	0.528	9.21E-07	6	Mrps16
5.90E-11	0.2972131	0.341	0.232	9.26E-07	6	Pnp
6.02E-11	0.36096265	0.931	0.91	9.43E-07	6	Anxa1
6.26E-11	-0.2819215	0.894	0.942	9.82E-07	6	Aldh2
6.62E-11	0.27603157	0.212	0.105	1.04E-06	6	Siah2
7.15E-11	-0.2910976	0.134	0.298	1.12E-06	6	Pnck
8.19E-11	-0.3211519	0.338	0.526	1.28E-06	6	Zc2hc1a
8.20E-11	0.3017572	0.519	0.399	1.29E-06	6	Rsl1d1
8.85E-11	-0.2557142	0.1	0.252	1.39E-06	6	Fuz
8.89E-11	0.32690738	0.787	0.716	1.39E-06	6	Eno1
9.59E-11	-0.3188014	0.466	0.644	1.50E-06	6	Commd3
9.90E-11	0.26544396	0.603	0.576	1.55E-06	6	Acot9
1.01E-10	-0.3087763	0.625	0.78	1.58E-06	6	Smim11
1.03E-10	-0.2689826	0.184	0.361	1.62E-06	6	Eef2k
1.08E-10	-0.2742155	0.872	0.923	1.69E-06	6	Gnai2
1.11E-10	0.33108948	0.591	0.496	1.74E-06	6	Cav3
1.21E-10	0.25772248	0.269	0.164	1.90E-06	6	Bcor
1.22E-10	-0.3320191	0.603	0.75	1.91E-06	6	Slc48a1
1.39E-10	0.31553482	0.734	0.63	2.17E-06	6	Sfpq
1.42E-10	0.30777479	0.778	0.706	2.22E-06	6	Anxa7
1.53E-10	0.51859897	0.684	0.655	2.41E-06	6	Klf4
1.54E-10	-0.3127719	0.637	0.762	2.42E-06	6	Rab11b
1.57E-10	-0.3373509	0.338	0.515	2.46E-06	6	Fez1
1.67E-10	0.32825342	0.519	0.422	2.61E-06	6	Lrrc8a
1.67E-10	-0.2998034	0.863	0.917	2.62E-06	6	Selenom
1.78E-10	0.25809498	0.397	0.298	2.78E-06	6	Eif3j1
1.87E-10	-0.2634911	0.928	0.949	2.94E-06	6	Smim14
1.88E-10	-0.2897274	0.409	0.599	2.95E-06	6	Mrps18c
1.96E-10	-0.3157276	0.419	0.603	3.07E-06	6	Spr
1.97E-10	0.28166593	0.681	0.599	3.09E-06	6	Cldnd1
1.99E-10	0.32509756	0.844	0.801	3.12E-06	6	Rock2
2.01E-10	-0.266497	0.269	0.454	3.14E-06	6	Pcbd2
2.09E-10	0.33006535	0.894	0.872	3.27E-06	6	Tgm2
2.15E-10	-0.2500413	0.941	0.966	3.38E-06	6	Capzb
2.22E-10	-0.2898148	0.2	0.376	3.48E-06	6	Anln
2.28E-10	0.27195606	0.312	0.211	3.57E-06	6	Kdm5b
2.28E-10	0.27649288	0.509	0.431	3.58E-06	6	Ahsa2
2.34E-10	-0.2849391	0.522	0.7	3.68E-06	6	Sdhc
2.48E-10	-0.268032	0.084	0.228	3.89E-06	6	Gm26802
2.49E-10	-0.3008364	0.347	0.53	3.91E-06	6	C1d
2.57E-10	-0.2782016	0.703	0.837	4.04E-06	6	Ehd2

2.71E-10	-0.3020797	0.322	0.509	4.25E-06	6	Synpo
2.75E-10	-0.2537769	0.144	0.305	4.31E-06	6	Ppil1
2.82E-10	0.29073764	0.597	0.517	4.43E-06	6	Ptp4a1
2.91E-10	-0.2859749	0.191	0.362	4.57E-06	6	Cuedc1
3.05E-10	-0.2979336	0.488	0.667	4.78E-06	6	Txn2
3.15E-10	0.283823	0.65	0.575	4.94E-06	6	Ube2a
3.19E-10	0.27857423	0.988	0.973	5.01E-06	6	Calr
3.23E-10	0.28697743	0.869	0.825	5.07E-06	6	Tpm4
3.27E-10	0.31530595	0.506	0.378	5.13E-06	6	Prnp
3.28E-10	-0.3058469	0.297	0.477	5.14E-06	6	Lsm3
3.35E-10	-0.3277254	0.306	0.478	5.25E-06	6	Cbx6
3.51E-10	-0.2854691	0.753	0.875	5.51E-06	6	Tmem256
3.57E-10	-0.2782811	0.503	0.684	5.60E-06	6	Sptssa
3.62E-10	-0.2519548	0.122	0.278	5.68E-06	6	Nudt18
3.67E-10	-0.2876655	0.759	0.866	5.76E-06	6	Rtraf
3.70E-10	-0.2708341	0.844	0.92	5.80E-06	6	Smdt1
3.86E-10	0.32583675	0.803	0.712	6.05E-06	6	Sf3b1
4.02E-10	-0.3466756	0.381	0.556	6.30E-06	6	Plekho1
4.24E-10	-0.3292568	0.644	0.785	6.65E-06	6	Gstm2
4.31E-10	0.29581283	0.688	0.597	6.76E-06	6	Luzp1
4.45E-10	0.27131868	0.7	0.633	6.98E-06	6	Ppp2ca
4.63E-10	-0.3204207	0.344	0.525	7.25E-06	6	Pdlim2
4.77E-10	-0.3238764	0.366	0.538	7.47E-06	6	Unc45a
4.94E-10	0.32698973	0.481	0.325	7.75E-06	6	Tgfb1
5.11E-10	-0.280897	0.547	0.72	8.01E-06	6	Tpr
5.33E-10	-0.256007	0.144	0.304	8.35E-06	6	Cdkl5
5.70E-10	-0.2904486	0.444	0.619	8.95E-06	6	Cisd1
6.16E-10	-0.3375782	0.428	0.609	9.66E-06	6	Ccdc3
6.47E-10	0.26328111	0.388	0.27	1.02E-05	6	Smad1
6.52E-10	-0.300249	0.75	0.847	1.02E-05	6	Ndufb4
6.86E-10	0.30084257	0.609	0.504	1.08E-05	6	Pkn2
7.03E-10	-0.3168975	0.347	0.523	1.10E-05	6	Itga1
7.52E-10	0.29852105	0.353	0.233	1.18E-05	6	Arid5a
7.64E-10	-0.4797625	0.497	0.635	1.20E-05	6	Igfbp4
7.80E-10	-0.3024188	0.284	0.454	1.22E-05	6	Smim26
7.98E-10	0.29832138	0.947	0.939	1.25E-05	6	Pmepa1
8.40E-10	-0.2585259	0.138	0.294	1.32E-05	6	Zbtb16
8.56E-10	0.28290898	0.672	0.592	1.34E-05	6	Rnf11
8.65E-10	-0.2638797	0.181	0.348	1.36E-05	6	Bcar3
8.66E-10	-0.2791622	0.956	0.976	1.36E-05	6	Cavin3
9.13E-10	0.27322593	0.484	0.364	1.43E-05	6	Adss
9.51E-10	-0.2790012	0.462	0.641	1.49E-05	6	Churc1
1.01E-09	0.34097078	0.566	0.513	1.58E-05	6	Plpp3
1.05E-09	0.27151361	0.672	0.636	1.64E-05	6	Stip1
1.11E-09	-0.3035692	0.466	0.633	1.74E-05	6	Mrpl51
1.14E-09	-0.2521165	0.203	0.373	1.80E-05	6	Med30
1.17E-09	-0.4087193	0.294	0.472	1.83E-05	6	Sfrp2
1.33E-09	-0.2818003	0.406	0.587	2.09E-05	6	Mcrip1
1.34E-09	0.26106511	0.488	0.377	2.09E-05	6	Map2k1
1.34E-09	-0.3159599	0.462	0.632	2.10E-05	6	Idh2
1.53E-09	-0.2775078	0.903	0.939	2.40E-05	6	Pcdh7

1.56E-09	-0.271952	0.331	0.512	2.45E-05	6	Mtch1
1.66E-09	0.25334272	0.334	0.25	2.60E-05	6	Rnf19a
1.69E-09	-0.2984221	0.3	0.476	2.65E-05	6	Nkd1
1.77E-09	-0.2838365	0.772	0.878	2.77E-05	6	Ndufa3
1.78E-09	-0.2557444	0.719	0.848	2.79E-05	6	Chmp2a
1.79E-09	0.27142085	0.609	0.489	2.80E-05	6	Zc3h15
1.81E-09	-0.2819046	0.606	0.757	2.84E-05	6	Lamtor5
1.89E-09	-0.3078864	0.409	0.583	2.96E-05	6	Ndufaf8
1.95E-09	-0.2844093	0.859	0.92	3.06E-05	6	Oxct1
1.96E-09	-0.2853308	0.734	0.842	3.07E-05	6	Tprgl
2.03E-09	-0.2693784	0.825	0.905	3.18E-05	6	Ndufa5
2.35E-09	-0.305638	0.616	0.761	3.68E-05	6	Anxa11
2.35E-09	0.29194409	0.991	0.988	3.68E-05	6	Tagln2
2.42E-09	0.31995033	0.988	0.977	3.79E-05	6	Postn
2.42E-09	-0.3565039	0.637	0.758	3.80E-05	6	Hist1h2bc
2.44E-09	-0.299218	0.484	0.647	3.82E-05	6	Snrnp27
2.47E-09	0.38674227	0.991	0.97	3.87E-05	6	Mustn1
2.56E-09	0.25444695	0.459	0.383	4.02E-05	6	Ppp2r2a
2.66E-09	-0.3077452	0.244	0.407	4.17E-05	6	Dact3
2.82E-09	-0.2953093	0.678	0.783	4.43E-05	6	Myh10
2.94E-09	-0.3117237	0.3	0.469	4.60E-05	6	Gstt1
3.14E-09	-0.2913747	0.209	0.371	4.93E-05	6	Filip1
3.33E-09	0.29234384	0.725	0.644	5.23E-05	6	Hnrnpdl
3.42E-09	-0.2901725	0.341	0.515	5.37E-05	6	Kank2
3.43E-09	-0.3465179	0.806	0.883	5.38E-05	6	Id2
3.53E-09	0.25337365	0.309	0.177	5.54E-05	6	Shroom3
3.59E-09	-0.2959096	0.584	0.738	5.63E-05	6	Rsrp1
3.76E-09	-0.2749906	0.488	0.657	5.89E-05	6	Naa38
3.77E-09	-0.2768665	0.319	0.492	5.91E-05	6	Mat2b
3.89E-09	-0.2566075	0.847	0.917	6.10E-05	6	Mpc1
3.94E-09	-0.2715524	0.834	0.91	6.18E-05	6	Mbnl1
3.96E-09	0.28403827	0.466	0.365	6.20E-05	6	Mcm6
4.30E-09	0.33676142	0.747	0.667	6.74E-05	6	Pdlim5
4.35E-09	-0.2538577	0.259	0.43	6.83E-05	6	[illegible]210016F16Ri[illegible]
4.68E-09	-0.3271761	0.694	0.816	7.33E-05	6	Mcam
4.70E-09	0.28392858	0.356	0.255	7.37E-05	6	Vmp1
5.61E-09	0.26293546	0.597	0.532	8.80E-05	6	Anp32e
5.66E-09	-0.3272813	0.531	0.663	8.88E-05	6	Pink1
5.78E-09	0.25316126	0.906	0.843	9.07E-05	6	Sub1
5.82E-09	0.25584093	0.85	0.81	9.12E-05	6	Eif1ax
5.90E-09	-0.2657275	0.647	0.783	9.25E-05	6	Brk1
5.92E-09	-0.4178487	0.616	0.749	9.28E-05	6	Id1
6.58E-09	0.27792508	0.506	0.413	0.00010313	6	Tspan5
6.65E-09	-0.2801621	0.119	0.261	0.00010421	6	Adm
6.67E-09	-0.2724075	0.887	0.936	0.00010457	6	Ndufv3
6.82E-09	0.26868017	0.822	0.757	0.00010702	6	Snu13
6.84E-09	-0.2592251	0.266	0.433	0.00010721	6	Pde6d
7.04E-09	-0.3142832	0.409	0.573	0.00011035	6	Rassf3
7.08E-09	-0.2981304	0.581	0.733	0.00011104	6	Kctd10
7.10E-09	-0.2768685	0.247	0.412	0.00011141	6	Arhgef2
7.69E-09	0.29255072	0.388	0.301	0.00012065	6	Ctsc

7.82E-09	-0.2794723	0.644	0.782	0.00012257	6	Rnf7
8.18E-09	-0.286582	0.312	0.483	0.00012832	6	Myom1
8.78E-09	-0.2989487	0.359	0.526	0.00013767	6	Hand2
9.11E-09	-0.2839357	0.331	0.499	0.0001429	6	Smarca2
9.60E-09	0.28534366	0.869	0.818	0.00015057	6	Pgrmc1
9.93E-09	0.43823313	0.478	0.434	0.00015574	6	Meg3
9.96E-09	-0.2533766	0.153	0.302	0.00015614	6	Tef
1.01E-08	-0.258029	0.172	0.325	0.0001586	6	Tmcc3
1.07E-08	-0.2979596	0.272	0.422	0.0001685	6	Mob2
1.08E-08	-0.290514	0.634	0.761	0.00016905	6	Mrpl42
1.09E-08	-0.2736426	0.406	0.574	0.00017087	6	Coro1c
1.13E-08	-0.2655512	0.284	0.446	0.00017719	6	Nfu1
1.17E-08	-0.3083664	0.369	0.532	0.0001833	6	Bod1l
1.22E-08	0.2577216	0.912	0.861	0.00019101	6	Mif
1.37E-08	-0.2682695	0.272	0.43	0.00021485	6	Eci2
1.42E-08	-0.2609006	0.8	0.888	0.00022274	6	Anxa6
1.43E-08	-0.2846708	0.462	0.625	0.00022408	6	Snx17
1.43E-08	0.34813168	0.953	0.937	0.0002245	6	S100a4
1.48E-08	-0.2596339	0.228	0.383	0.00023133	6	Dbnl
1.61E-08	0.2682471	0.322	0.219	0.00025213	6	Mapk1ip1l
1.63E-08	-0.2651406	0.372	0.54	0.00025489	6	Dmac1
1.65E-08	0.27954292	0.572	0.499	0.00025935	6	Cggbp1
1.66E-08	-0.2714283	0.469	0.633	0.00026016	6	Foxn3
1.69E-08	-0.3224373	0.469	0.627	0.00026466	6	Klf13
1.75E-08	-0.2530677	0.597	0.745	0.00027429	6	Tomm22
1.77E-08	-0.2701923	0.628	0.768	0.00027806	6	Copz2
1.80E-08	-0.2709936	0.356	0.52	0.00028275	6	Fez2
1.87E-08	-0.2866059	0.784	0.88	0.00029387	6	Prelp
1.93E-08	-0.3017844	0.288	0.445	0.00030221	6	Gm11627
1.97E-08	-0.2580055	0.753	0.862	0.00030903	6	Rnf187
2.02E-08	-0.2531029	0.65	0.781	0.00031622	6	Mrps14
2.03E-08	0.41601692	0.734	0.714	0.00031903	6	Nfkbia
2.06E-08	0.28051647	0.272	0.199	0.00032305	6	Nop16
2.06E-08	-0.2596969	0.334	0.503	0.00032357	6	Serpinb6b
2.08E-08	-0.2595916	0.356	0.526	0.00032635	6	Mrpl34
2.15E-08	0.26484029	0.756	0.731	0.00033716	6	Pdgfa
2.28E-08	0.28738998	0.622	0.502	0.00035794	6	Plec
2.50E-08	-0.279809	0.553	0.691	0.00039252	6	2-Mar
2.55E-08	-0.2740218	0.4	0.562	0.00040046	6	Rtl8a
2.58E-08	0.2622718	0.444	0.338	0.00040468	6	Akirin1
2.75E-08	-0.2765799	0.422	0.58	0.00043117	6	Cenpx
3.22E-08	-0.3089116	0.488	0.629	0.00050478	6	Atp2b4
3.23E-08	-0.3325142	0.644	0.756	0.00050622	6	Ecm2
3.45E-08	0.26781578	0.666	0.594	0.00054128	6	Fgfr1
3.46E-08	0.25969029	0.613	0.548	0.00054274	6	Smarca5
3.65E-08	-0.2869406	0.494	0.64	0.00057204	6	Acat1
3.86E-08	0.34682796	0.506	0.588	0.00060573	6	Klf2
4.51E-08	0.25267711	0.581	0.464	0.0007067	6	Rab5a
4.76E-08	-0.2851776	0.434	0.585	0.00074684	6	Txndc15
4.98E-08	-0.250411	0.491	0.652	0.00078054	6	Ddx39b
5.58E-08	-0.282845	0.884	0.933	0.00087479	6	Pdlim1

5.64E-08	-0.2754849	0.662	0.788	0.00088477	6	Banf1
5.73E-08	-0.2509463	0.591	0.734	0.00089923	6	Tmem14c
5.76E-08	-0.2716609	0.316	0.459	0.0009037	6	Chrac1
6.10E-08	-0.3115146	0.397	0.543	0.00095599	6	Tob2
6.17E-08	-0.2661784	0.506	0.657	0.00096759	6	Dctn2
6.75E-08	-0.2905058	0.35	0.501	0.00105793	6	Pde5a
6.84E-08	-0.2586583	0.3	0.46	0.00107192	6	Pcmtd1
6.90E-08	0.25111347	0.684	0.606	0.00108249	6	Mical2
7.85E-08	-0.2508204	0.597	0.737	0.00123068	6	Trmt112
7.87E-08	-0.2577602	0.734	0.831	0.00123398	6	Tecr
8.06E-08	-0.275905	0.616	0.748	0.00126327	6	Nfix
8.89E-08	-0.2580608	0.212	0.356	0.00139328	6	Mif4gd
9.12E-08	-0.2673558	0.759	0.847	0.0014302	6	Rex1bd
9.43E-08	-0.2651137	0.444	0.598	0.00147813	6	?610001J05Ril
9.43E-08	0.25011804	0.569	0.487	0.00147941	6	Tnfrsf1a
9.64E-08	-0.2513407	0.391	0.547	0.00151165	6	Tm2d2
9.79E-08	-0.2529336	0.197	0.344	0.00153445	6	Tmtc1
1.09E-07	-0.2587944	0.256	0.402	0.00170572	6	Snapin
1.24E-07	0.26820438	0.853	0.79	0.00194481	6	Atp1a1
1.32E-07	-0.3014294	0.716	0.791	0.00207283	6	Efhd1
1.38E-07	-0.2895213	0.278	0.43	0.00215704	6	Gxylt2
1.46E-07	-0.2582537	0.425	0.582	0.00229093	6	Aimp1
1.60E-07	-0.3028013	0.394	0.536	0.00250342	6	Emilin1
1.71E-07	-0.2676437	0.431	0.584	0.0026776	6	Dazap2
1.71E-07	-0.2943707	0.703	0.796	0.00268425	6	Itgb5
1.78E-07	0.28346795	0.659	0.565	0.00278763	6	Pole4
1.78E-07	0.41539228	0.928	0.929	0.00279415	6	Fabp4
1.79E-07	-0.2661352	0.444	0.598	0.00280649	6	Chd3
2.11E-07	-0.275044	0.45	0.604	0.00330681	6	Lgr6
2.15E-07	-0.2578202	0.794	0.852	0.0033656	6	Mdh1
2.24E-07	-0.2613333	0.391	0.534	0.00351914	6	Lamtor1
2.28E-07	-0.2523445	0.312	0.463	0.00357548	6	BC004004
2.30E-07	0.25704771	0.547	0.435	0.00360131	6	Ywhag
2.36E-07	-0.2736312	0.562	0.7	0.00370798	6	Jph2
2.82E-07	-0.3016927	0.672	0.783	0.0044186	6	Spint2
2.84E-07	-0.2792173	0.6	0.72	0.00445021	6	Ccni
2.85E-07	0.2949829	0.166	0.085	0.00446763	6	Sox9
2.99E-07	-0.2570968	0.403	0.555	0.00469366	6	Ik
3.04E-07	-0.2626841	0.469	0.615	0.00477225	6	?230219D22Ril
3.12E-07	-0.2544559	0.613	0.744	0.00489878	6	Ccser2
3.42E-07	-0.2703704	0.672	0.775	0.00536365	6	Thra
3.86E-07	0.25233582	0.319	0.208	0.00605433	6	B4galt1
4.17E-07	-0.2712731	0.256	0.398	0.00654246	6	Dsp
4.20E-07	0.27087225	0.197	0.128	0.00659012	6	Sox4
4.34E-07	-0.2635267	0.338	0.476	0.00679855	6	Mrpl36
4.45E-07	-0.2555112	0.575	0.709	0.00697092	6	Pigp
4.50E-07	-0.2586633	0.581	0.716	0.0070624	6	?310022B05Ril
4.52E-07	0.30194284	0.209	0.128	0.00708558	6	Spry2
4.93E-07	-0.2570377	0.753	0.841	0.00773203	6	Selenop
5.70E-07	-0.254591	0.569	0.704	0.00894196	6	Efemp2
6.33E-07	-0.2506822	0.372	0.514	0.00993372	6	Fam174a

6.84E-07	0.25444387	0.722	0.652	0.01072529	6	Glud1
7.38E-07	-0.263709	0.362	0.508	0.0115773	6	Atp6v1a
7.47E-07	-0.2561044	0.684	0.799	0.0117111	6	Fam129a
8.26E-07	-0.2506913	0.613	0.734	0.01295782	6	Timm8b
1.04E-06	-0.2722855	0.225	0.356	0.01629822	6	Rgs7bp
1.30E-06	-0.2516629	0.422	0.563	0.02033646	6	Mpnd
1.49E-06	0.28323856	0.994	0.998	0.02329953	6	Actg1
1.69E-06	0.3010749	0.45	0.369	0.02648072	6	Fabp3
1.79E-06	-0.2594263	0.488	0.612	0.02810517	6	Fbn1
2.35E-06	0.27381262	0.256	0.186	0.03686369	6	Abhd2
2.42E-06	-0.2708986	0.719	0.79	0.03796439	6	Plcb4
2.75E-06	0.25851226	0.863	0.837	0.04318308	6	Cstb
3.06E-06	-0.2560953	0.584	0.705	0.04794844	6	Celf2
3.64E-06	0.43850589	0.647	0.59	0.057145	6	Cebpd
6.85E-06	0.32090383	0.453	0.375	0.10738331	6	Palld
7.77E-06	-0.2880536	0.178	0.293	0.12187411	6	Timp4
9.99E-06	0.27294713	0.684	0.622	0.15673127	6	Hnrnph1
2.01E-05	0.3263617	0.822	0.787	0.31587523	6	Itih4
2.06E-05	0.2608182	0.953	0.945	0.32293183	6	Clu
3.95E-05	-0.2526643	0.95	0.954	0.61921289	6	Cnn3
5.34E-05	0.26713136	1	0.999	0.8379893	6	Mgp
0.0040831	0.30276498	0.462	0.423	1	6	Slc39a1
0.00555175	0.32534624	0.953	0.938	1	6	Pam
0	4.06371116	0.986	0.788	0	8	Mt2
0	-1.1515671	1	0.997	0	8	Dstn
[illegible]11328103419	-1.4785684	0.969	0.998	[illegible]69923598971[illegible]	8	Myl9
5.50E-280	1.75091799	0.997	0.857	8.63E-276	8	Mif
1.36E-279	2.09530782	1	0.942	2.13E-275	8	Clu
1.09E-243	3.09571672	1	0.976	1.72E-239	8	Mt1
2.32E-215	-1.2300172	0.958	0.996	3.64E-211	8	Tpm1
4.67E-214	-1.3653607	0.944	0.995	7.32E-210	8	Myh11
6.36E-213	1.56755418	0.983	0.942	9.97E-209	8	Ifitm3
3.28E-208	3.34231067	0.899	0.277	5.14E-204	8	Timp1
1.07E-206	1.08904227	1	0.992	1.68E-202	8	Cd63
1.16E-205	2.2347458	0.767	0.339	1.82E-201	8	Gm12840
3.83E-197	-1.0663372	1	0.999	6.00E-193	8	Acta2
2.11E-188	3.65010991	0.573	0.021	3.31E-184	8	Lum
3.59E-188	-1.5239604	0.819	0.988	5.62E-184	8	Gstm1
3.55E-182	-0.9188279	0.997	0.998	5.57E-178	8	Tpm2
5.33E-178	-0.7994851	0.997	0.998	8.35E-174	8	Tagln
1.02E-171	-0.714081	1	0.999	1.59E-167	8	Myl6
3.49E-167	3.25343879	0.712	0.126	5.48E-163	8	Vcam1
3.37E-165	1.50815645	0.858	0.338	5.29E-161	8	Frzb
2.06E-153	-0.7789624	0.997	0.997	3.23E-149	8	Cpe
2.52E-147	-1.1504918	0.878	0.994	3.96E-143	8	Cnn1
4.02E-146	-0.8230663	1	0.998	6.30E-142	8	Vim
5.69E-142	1.13445547	0.983	0.912	8.92E-138	8	Ldha
8.43E-134	1.52256814	0.538	0.048	1.32E-129	8	Cp
2.11E-131	-0.925216	0.99	0.995	3.32E-127	8	Flna
5.59E-121	4.9475049	0.392	0.024	8.77E-117	8	Spp1
1.76E-120	1.13334628	0.538	0.044	2.77E-116	8	Tmem176b

2.61E-116	1.51938224	0.726	0.255	4.09E-112	8	Cxcl12
2.33E-106	1.0665174	1	0.993	3.65E-102	8	Tm4sf1
3.53E-106	-1.041476	0.903	0.985	5.54E-102	8	Map1b
1.31E-104	1.08194615	0.983	0.94	2.06E-100	8	Col1a1
7.20E-102	-1.8278748	0.448	0.879	1.13E-97	8	Sparcl1
7.77E-98	1.27497909	0.958	0.752	1.22E-93	8	Sdc4
1.29E-96	0.64185504	1	0.999	2.02E-92	8	Rpl41
5.15E-94	-0.6307571	0.979	0.997	8.08E-90	8	Cald1
2.35E-91	-1.7675557	0.399	0.858	3.69E-87	8	Sost
1.94E-86	-0.9799254	0.962	0.991	3.04E-82	8	Csrp1
7.81E-86	-0.9570006	0.951	0.988	1.23E-81	8	Gsn
3.08E-84	-0.944756	0.854	0.975	4.83E-80	8	Ppp1r12a
2.90E-83	-0.8626465	0.917	0.98	4.55E-79	8	Tns1
2.42E-82	0.88955775	0.913	0.672	3.80E-78	8	Pgk1
1.34E-81	2.0926675	0.236	0.003	2.11E-77	8	Lcn2
1.05E-80	0.89256617	0.899	0.71	1.65E-76	8	Eno1
5.12E-80	-0.8547531	0.906	0.982	8.03E-76	8	Mylk
8.05E-80	-1.3324108	0.639	0.904	1.26E-75	8	Ckb
1.91E-79	1.02048865	0.573	0.149	2.99E-75	8	Cox4i2
2.31E-79	-0.9887059	0.788	0.948	3.62E-75	8	Cped1
1.72E-78	1.01953077	0.503	0.095	2.70E-74	8	Tnc
2.23E-78	-1.050821	0.819	0.962	3.50E-74	8	Cav1
3.63E-77	-0.6862104	0.965	0.993	5.69E-73	8	Actn1
8.40E-77	0.96285205	0.396	0.039	1.32E-72	8	C4b
3.41E-76	0.68448492	0.99	0.99	5.35E-72	8	Gapdh
1.55E-75	0.78541717	0.469	0.07	2.42E-71	8	Sdc3
2.31E-75	-1.3455316	0.559	0.902	3.62E-71	8	Prss23
7.32E-74	0.6013461	0.306	0.014	1.15E-69	8	Tmem176a
8.00E-74	-0.5840963	0.997	0.997	1.25E-69	8	Dynll1
9.36E-73	-0.9299165	0.812	0.96	1.47E-68	8	Zyx
9.47E-73	0.69405803	0.997	0.993	1.49E-68	8	Rps28
1.39E-72	0.9676169	0.212	0.002	2.18E-68	8	Ndufa4l2
2.71E-71	0.65967981	1	0.991	4.26E-67	8	Rpl38
5.19E-68	-1.2647654	0.521	0.864	8.14E-64	8	Ogn
9.83E-66	0.74084139	0.979	0.969	1.54E-61	8	Ifitm2
5.92E-65	0.82929521	0.948	0.897	9.29E-61	8	Ctsl
7.67E-65	-0.8498278	0.826	0.963	1.20E-60	8	Hcfc1r1
1.24E-64	-1.0020217	0.549	0.87	1.95E-60	8	Synpo2
2.95E-63	-0.9059954	0.816	0.967	4.63E-59	8	Rock1
2.05E-62	0.68572966	0.986	0.971	3.21E-58	8	Pkm
1.17E-61	0.80202417	0.809	0.543	1.83E-57	8	Ppic
2.25E-61	-0.8018117	0.872	0.971	3.53E-57	8	Lmod1
1.45E-60	-0.8620567	0.889	0.98	2.28E-56	8	Ppp1r14a
1.75E-60	1.19921291	0.389	0.106	2.75E-56	8	Tnfrsf11b
1.67E-59	-0.8838611	0.74	0.942	2.62E-55	8	Ltbp4
1.71E-59	-1.1581553	0.788	0.956	2.68E-55	8	Nov
3.72E-59	-0.7167362	0.882	0.97	5.83E-55	8	Cavin1
6.71E-59	-0.6052041	0.962	0.99	1.05E-54	8	Itga8
1.28E-58	-0.7611183	0.785	0.965	2.00E-54	8	Ramp1
1.90E-58	0.55541748	0.299	0.028	2.98E-54	8	Cx3cl1
1.23E-57	-0.9895934	0.382	0.768	1.92E-53	8	Ecm2

2.16E-57	0.57563603	0.361	0.046	3.39E-53	8	Casp4
2.55E-57	0.66079956	0.993	0.969	4.01E-53	8	Ctsb
8.52E-57	-1.1988121	0.257	0.7	1.34E-52	8	Mylk4
1.55E-56	0.5581283	1	0.996	2.44E-52	8	Rplp1
2.08E-56	1.13464123	0.774	0.584	3.26E-52	8	Cebpd
2.26E-56	-0.8877466	0.656	0.899	3.54E-52	8	Wfdc1
3.23E-56	-0.9940263	0.312	0.718	5.06E-52	8	Optc
1.04E-55	-0.7174541	0.997	0.998	1.63E-51	8	Crip1
1.32E-55	-0.8398689	0.747	0.938	2.08E-51	8	Sh3bgr
1.62E-55	-0.9271175	0.59	0.865	2.53E-51	8	Lims2
2.78E-55	-0.4703123	0.993	0.996	4.35E-51	8	Cd9
5.42E-55	0.92079813	0.153	0.001	8.50E-51	8	Prg4
1.16E-54	-0.8173458	0.802	0.961	1.82E-50	8	Hspb6
1.35E-54	0.92411376	0.799	0.588	2.12E-50	8	Pi15
2.97E-54	-0.4599124	0.99	0.995	4.65E-50	8	Gnas
3.64E-54	-1.0974587	0.625	0.892	5.70E-50	8	Tppp3
8.92E-54	0.81934512	0.851	0.587	1.40E-49	8	Tspan4
1.69E-53	0.50583624	1	0.996	2.65E-49	8	Rps29
3.13E-53	0.62448032	1	0.996	4.91E-49	8	Sparc
1.74E-52	0.70362108	0.927	0.798	2.74E-48	8	Mrpl52
2.55E-52	0.73074367	0.538	0.177	4.00E-48	8	Pde1a
3.09E-52	-1.245073	0.403	0.768	4.84E-48	8	Fbxl22
4.84E-52	0.50209448	1	0.996	7.58E-48	8	Rplp2
1.12E-51	0.59937956	0.976	0.959	1.76E-47	8	Bsg
1.64E-51	-0.4837794	0.979	0.99	2.58E-47	8	Ybx1
1.67E-51	0.52923897	1	0.998	2.61E-47	8	Rpl37a
1.93E-51	-1.2708266	0.611	0.833	3.03E-47	8	Nr4a1
6.12E-51	-1.1904049	0.556	0.841	9.60E-47	8	Btg2
8.27E-50	1.56846298	0.219	0.015	1.30E-45	8	Dcn
1.43E-49	0.69620688	0.851	0.647	2.24E-45	8	Lgmn
7.42E-49	1.39797758	0.347	0.059	1.16E-44	8	Thbs1
1.19E-48	1.04942043	0.955	0.915	1.86E-44	8	Ctgf
1.27E-48	-0.7072931	0.83	0.945	1.98E-44	8	Aldh2
1.35E-48	0.62286142	0.462	0.111	2.11E-44	8	C1ra
1.47E-48	-0.7148159	0.84	0.96	2.31E-44	8	Lrrfip1
4.38E-48	-0.7637189	0.753	0.941	6.86E-44	8	Pdlim3
2.28E-47	0.75661855	0.767	0.501	3.57E-43	8	Hif1a
3.16E-47	0.33925606	0.146	0.002	4.95E-43	8	Car9
1.04E-45	-1.0098476	0.278	0.658	1.63E-41	8	Fibin
1.16E-45	0.8270507	0.74	0.505	1.82E-41	8	Plpp3
2.45E-45	-0.8179949	0.847	0.966	3.84E-41	8	Filip1l
1.26E-44	1.05158393	1	0.999	1.98E-40	8	Mgp
2.75E-44	0.76862816	0.67	0.345	4.31E-40	8	Fgf2
4.27E-44	0.70792862	0.618	0.318	6.69E-40	8	Pfkl
6.45E-44	0.41166934	1	0.997	1.01E-39	8	Igfbp7
9.17E-44	-0.9127685	0.885	0.955	1.44E-39	8	Dusp1
2.49E-43	-0.5849676	0.955	0.989	3.90E-39	8	Ptgis
6.87E-43	1.09227393	0.986	0.982	1.08E-38	8	Eln
1.33E-42	0.68189408	0.535	0.21	2.08E-38	8	Map4k4
1.48E-42	-0.3077371	1	1	2.32E-38	8	Tmsb4x
3.34E-42	-1.0748536	0.969	0.975	5.23E-38	8	Fos

4.37E-42	-0.6254827	0.83	0.952	6.86E-38	8	Ppp1cb
6.00E-42	0.67399702	0.885	0.762	9.40E-38	8	Fam162a
1.12E-41	0.53549138	0.417	0.099	1.75E-37	8	Il13ra1
1.14E-41	-0.4044945	0.99	0.997	1.79E-37	8	Csrp2
2.45E-41	0.75146803	0.983	0.976	3.84E-37	8	Col3a1
5.09E-41	-1.0024894	0.667	0.89	7.98E-37	8	Id2
3.47E-40	-0.6673971	0.767	0.924	5.45E-36	8	Oxct1
8.22E-40	0.69606312	0.872	0.726	1.29E-35	8	Col5a2
8.94E-40	-0.7181266	0.611	0.859	1.40E-35	8	Adcy5
9.94E-40	0.56103271	0.931	0.882	1.56E-35	8	Ssr4
1.25E-39	0.50172718	0.253	0.033	1.97E-35	8	Lrg1
1.50E-39	0.76111364	0.861	0.702	2.36E-35	8	Lox
2.03E-39	0.95047607	0.91	0.783	3.18E-35	8	Itih4
4.47E-39	0.79565077	0.799	0.636	7.01E-35	8	Cdo1
6.71E-39	0.65229033	0.76	0.504	1.05E-34	8	Stk38l
1.67E-38	0.39494162	1	0.998	2.61E-34	8	Rpl37
8.32E-38	0.55302359	0.406	0.107	1.31E-33	8	Osmr
5.36E-37	-0.6048922	0.868	0.965	8.40E-33	8	Pbxip1
1.00E-36	0.45408414	1	0.994	1.57E-32	8	Rpl39
1.00E-36	0.5864941	0.42	0.139	1.57E-32	8	Slc16a3
2.97E-36	2.27976835	0.184	0.047	4.65E-32	8	Cxcl1
3.15E-36	-0.7661017	0.431	0.726	4.94E-32	8	St5
8.81E-36	0.62471755	0.639	0.329	1.38E-31	8	Pros1
2.36E-35	0.42229538	1	0.996	3.71E-31	8	Serf2
4.29E-34	0.51374954	0.892	0.838	6.72E-30	8	Pgam1
6.12E-34	0.41739748	0.997	0.993	9.59E-30	8	Rps26
6.70E-34	-0.7819365	0.608	0.82	1.05E-29	8	Mcam
9.87E-34	-0.6167826	0.816	0.945	1.55E-29	8	Slmap
1.09E-33	0.57253457	0.938	0.876	1.71E-29	8	Tpi1
2.56E-33	0.78373243	0.993	0.965	4.02E-29	8	Nupr1
3.79E-33	-0.5752168	0.757	0.912	5.94E-29	8	Rsu1
4.62E-33	0.44386088	1	0.985	7.25E-29	8	Tspo
5.00E-33	-0.7433831	0.389	0.695	7.85E-29	8	.190005I06Ril
6.92E-33	0.57195864	0.222	0.036	1.08E-28	8	Smoc2
1.48E-32	-0.7287304	0.274	0.616	2.33E-28	8	Ccdc3
1.71E-32	0.51820571	0.976	0.922	2.68E-28	8	Rpl36a1
2.53E-32	-0.6780096	0.865	0.959	3.97E-28	8	Cnn3
2.95E-32	-0.7233072	0.333	0.661	4.63E-28	8	Npy1r
7.12E-32	0.54758007	0.503	0.225	1.12E-27	8	Nnmt
8.66E-32	-0.5340861	0.878	0.947	1.36E-27	8	Ilk
1.13E-31	0.49557489	1	0.992	1.78E-27	8	Sod3
2.44E-31	-0.6815751	0.549	0.798	3.83E-27	8	Plcb4
2.64E-31	0.49970811	0.99	0.955	4.15E-27	8	Dbi
2.79E-31	-0.6564152	0.847	0.936	4.37E-27	8	Msrb1
3.12E-31	0.40366512	1	0.997	4.90E-27	8	Bgn
3.65E-31	0.56213314	0.823	0.488	5.72E-27	8	Fxyd5
4.05E-31	-0.7370902	0.503	0.754	6.35E-27	8	Limch1
4.48E-31	0.61623681	0.955	0.878	7.02E-27	8	Cd200
1.19E-30	0.60699987	0.965	0.865	1.86E-26	8	Tmsb10
1.36E-30	-0.5516056	0.892	0.967	2.14E-26	8	Npnt
2.01E-30	0.57177758	0.74	0.518	3.15E-26	8	Emilin1

2.15E-30	0.4139607	0.997	0.996	3.38E-26	8	Rplp0
2.17E-30	0.47870671	0.972	0.975	3.40E-26	8	Col1a2
2.84E-30	-0.6526908	0.715	0.883	4.46E-26	8	Cdh13
8.66E-30	0.56012321	0.51	0.235	1.36E-25	8	Gfm1
1.03E-29	0.60953741	0.594	0.338	1.61E-25	8	Scara3
1.09E-29	0.50222991	0.969	0.929	1.71E-25	8	Sec61b
4.32E-29	0.88350644	0.674	0.413	6.77E-25	8	Slc39a1
9.34E-29	-0.5625286	0.736	0.891	1.47E-24	8	Anxa6
9.81E-29	-0.4995015	0.92	0.963	1.54E-24	8	Map1lc3a
1.77E-28	-0.6705601	0.958	0.99	2.77E-24	8	Hsp90aa1
1.90E-28	-0.9757284	0.587	0.786	2.98E-24	8	Gadd45g
1.99E-28	-0.4918459	0.889	0.961	3.12E-24	8	Smtn
2.14E-28	-0.4848293	0.944	0.978	3.36E-24	8	Fblim1
5.04E-28	-0.4991665	0.938	0.988	7.90E-24	8	Fhl1
5.73E-28	-1.0876557	0.319	0.615	8.99E-24	8	Tcap
1.27E-27	-0.6194144	0.486	0.729	2.00E-23	8	Rasl12
2.18E-27	0.57306729	0.83	0.616	3.42E-23	8	Ltbp3
2.98E-27	-0.5461525	0.74	0.882	4.68E-23	8	Kif5b
4.71E-27	-0.6504526	0.413	0.663	7.39E-23	8	Pcp4l1
4.72E-27	0.59259344	0.507	0.228	7.41E-23	8	Glrx
6.60E-27	-0.3249965	1	0.997	1.03E-22	8	mt-Nd2
7.41E-27	-0.4996505	0.944	0.976	1.16E-22	8	Cavin3
2.30E-26	-0.5056415	0.882	0.951	3.60E-22	8	Bcam
2.59E-26	0.42738418	0.969	0.964	4.07E-22	8	Rpl36a
4.82E-26	0.38031142	0.997	0.992	7.56E-22	8	Rps18
1.06E-25	-0.6086397	0.483	0.736	1.66E-21	8	Ppp1r12b
1.99E-25	0.40482948	0.983	0.979	3.13E-21	8	Sec61g
2.16E-25	0.50278618	0.444	0.241	3.39E-21	8	Bnip3
2.17E-25	-0.5127524	0.767	0.858	3.41E-21	8	Cnn2
2.62E-25	-0.5170816	0.792	0.907	4.11E-21	8	Tgfb1i1
4.19E-25	-0.6412955	0.781	0.917	6.58E-21	8	Fmo2
5.00E-25	0.49832404	0.882	0.733	7.84E-21	8	Rps27rt
5.83E-25	-0.7757325	0.25	0.51	9.15E-21	8	Nr4a2
6.69E-25	-0.5696844	0.615	0.804	1.05E-20	8	Dmd
7.68E-25	-0.5284881	0.823	0.93	1.20E-20	8	Nexn
8.12E-25	-0.5038448	0.92	0.967	1.27E-20	8	Ahnak
9.25E-25	0.3886078	0.372	0.121	1.45E-20	8	Mlf1
1.04E-24	0.7343616	0.844	0.582	1.63E-20	8	Sncg
1.06E-24	-0.562428	0.861	0.951	1.66E-20	8	Ncam1
1.07E-24	-0.4794836	0.802	0.893	1.68E-20	8	Iscu
1.08E-24	0.63549094	0.118	0.012	1.70E-20	8	Icam1
1.61E-24	0.39369147	0.972	0.952	2.52E-20	8	Ppib
1.81E-24	-1.1888548	0.215	0.47	2.84E-20	8	Rgs2
1.92E-24	0.54946621	0.993	0.935	3.01E-20	8	S100a4
2.56E-24	0.43225253	0.969	0.955	4.02E-20	8	Eef1b2
2.70E-24	-0.4909463	0.865	0.958	4.24E-20	8	Lpp
2.84E-24	-0.5752678	0.469	0.726	4.45E-20	8	Smarcd3
3.04E-24	0.43578645	0.316	0.093	4.76E-20	8	Bhlhe40
4.26E-24	-0.3382542	0.962	0.991	6.68E-20	8	Slc25a4
5.53E-24	-0.5827166	0.444	0.697	8.67E-20	8	Pkp4
6.48E-24	-0.6865986	0.49	0.708	1.02E-19	8	Tmem110

8.12E-24	-0.545893	0.42	0.69	1.27E-19	8	Ttc28
8.47E-24	-0.6712275	0.382	0.632	1.33E-19	8	[illegible]030013I19Ril
1.20E-23	-0.4582569	0.903	0.967	1.88E-19	8	Sh3bgrl
1.40E-23	0.5086356	0.733	0.482	2.20E-19	8	Ckap4
1.71E-23	-0.4055391	0.958	0.978	2.67E-19	8	Ptms
1.93E-23	-0.5521805	0.431	0.66	3.03E-19	8	Snta1
2.79E-23	-0.77576	0.583	0.797	4.37E-19	8	Rrad
3.28E-23	0.43856546	0.26	0.072	5.15E-19	8	Fbln2
3.69E-23	0.52282211	0.75	0.54	5.78E-19	8	Serping1
3.90E-23	-0.5448965	0.965	0.986	6.12E-19	8	Ubc
3.93E-23	0.58848001	0.462	0.216	6.17E-19	8	Rspo3
4.34E-23	0.4485514	0.417	0.165	6.80E-19	8	Mrc2
4.38E-23	-0.4952802	0.698	0.885	6.87E-19	8	Sgcg
6.53E-23	0.41910495	0.326	0.106	1.02E-18	8	Map3k8
6.66E-23	0.51504646	0.965	0.942	1.04E-18	8	Fbln5
9.50E-23	-0.3756376	0.972	0.991	1.49E-18	8	H3f3a
9.84E-23	0.42076821	0.965	0.957	1.54E-18	8	Rpl13a
1.06E-22	-0.5321372	0.556	0.733	1.67E-18	8	Thsd4
1.26E-22	0.33412166	0.997	0.995	1.97E-18	8	Rpl32
1.46E-22	-0.5464052	0.49	0.724	2.29E-18	8	Sorl1
1.53E-22	0.46093073	0.91	0.8	2.40E-18	8	P4hb
1.82E-22	0.47256939	0.882	0.834	2.85E-18	8	Prdx5
2.04E-22	0.43469277	0.253	0.073	3.20E-18	8	Slc7a2
2.07E-22	0.368324	0.979	0.975	3.25E-18	8	Eef1g
2.12E-22	-0.5567433	0.24	0.513	3.33E-18	8	Ntf3
2.62E-22	-0.5491447	0.597	0.798	4.11E-18	8	Cdc42ep3
4.27E-22	0.5515385	0.625	0.37	6.70E-18	8	Mgst1
7.52E-22	-0.4354161	0.878	0.952	1.18E-17	8	Pdlim7
8.02E-22	0.32578343	0.34	0.113	1.26E-17	8	Sbno2
1.22E-21	-0.5589527	0.66	0.829	1.91E-17	8	Ccnd2
1.37E-21	-0.4812153	0.733	0.862	2.14E-17	8	Rnf187
1.99E-21	0.44104474	0.854	0.801	3.12E-17	8	Tmed3
2.97E-21	0.99486705	0.465	0.381	4.65E-17	8	2210407C18Ril
4.83E-21	-0.5322131	0.608	0.792	7.57E-17	8	Gucy1b1
6.47E-21	-0.5019431	0.531	0.736	1.01E-16	8	Utrn
8.61E-21	-0.725724	0.521	0.729	1.35E-16	8	Rbp4
9.55E-21	-0.3672629	0.976	0.992	1.50E-16	8	Cd81
1.19E-20	-0.5217867	0.281	0.548	1.87E-16	8	Lrrc17
1.25E-20	-0.5151909	0.59	0.779	1.96E-16	8	Thra
1.43E-20	-0.5392654	0.993	0.998	2.24E-16	8	Hspa8
1.47E-20	0.34276534	0.979	0.983	2.30E-16	8	Aldoa
1.52E-20	0.29128135	1	0.996	2.38E-16	8	Rps16
1.56E-20	0.46141779	0.747	0.598	2.44E-16	8	Fbn1
1.79E-20	-0.5997892	0.667	0.844	2.81E-16	8	Tinagl1
2.41E-20	-0.5418512	0.507	0.726	3.77E-16	8	Perp
2.59E-20	-0.2915704	1	0.997	4.06E-16	8	Actg1
2.65E-20	-0.4596334	0.705	0.843	4.16E-16	8	Tprgl
3.54E-20	0.29534902	1	0.997	5.56E-16	8	Rpl27a
3.75E-20	0.31472716	1	0.997	5.89E-16	8	Rps2
3.82E-20	-0.4157373	0.958	0.98	5.99E-16	8	Ddx5
4.49E-20	-0.5320458	0.517	0.744	7.04E-16	8	Atp1a2

4.83E-20	-0.521144	0.583	0.78	7.57E-16	8	Arl4a
4.98E-20	-0.4502554	0.753	0.87	7.80E-16	8	Dmpk
6.44E-20	-0.5138575	0.427	0.648	1.01E-15	8	Clip1
6.59E-20	-0.5348061	0.448	0.647	1.03E-15	8	Marveld1
8.87E-20	-0.5406968	0.753	0.864	1.39E-15	8	2010111I01Ril
1.03E-19	-0.7543599	0.774	0.92	1.62E-15	8	Cyr61
1.18E-19	-0.531254	0.372	0.601	1.84E-15	8	Islr
1.31E-19	0.47372107	0.712	0.548	2.06E-15	8	110008P14Ril
1.37E-19	-0.7920436	0.587	0.75	2.14E-15	8	Id1
1.40E-19	-0.5055266	0.295	0.547	2.19E-15	8	Kcnab1
1.45E-19	-0.4975776	0.747	0.887	2.27E-15	8	Cbr2
1.52E-19	-0.5711883	0.444	0.695	2.39E-15	8	Osr1
1.75E-19	0.6144103	0.681	0.481	2.74E-15	8	Gja1
1.78E-19	-0.6785631	0.569	0.734	2.80E-15	8	Gadd45b
3.06E-19	-0.5551921	0.167	0.396	4.79E-15	8	Haus8
3.29E-19	-0.4725407	0.677	0.834	5.16E-15	8	Tecr
3.57E-19	0.43010133	0.997	0.992	5.59E-15	8	Serpine2
3.77E-19	0.31220383	1	0.995	5.92E-15	8	Rps9
3.97E-19	0.29388437	0.993	0.996	6.22E-15	8	Rps27a
4.20E-19	0.39365812	0.979	0.937	6.59E-15	8	Rpl35
5.05E-19	-0.4066322	0.865	0.939	7.92E-15	8	Ywhaq
5.33E-19	-0.4825074	0.247	0.478	8.36E-15	8	Nkd1
7.62E-19	0.41016476	0.962	0.939	1.19E-14	8	Hsp90b1
7.71E-19	0.44723426	0.67	0.511	1.21E-14	8	Lxn
1.16E-18	-0.5044161	0.424	0.641	1.82E-14	8	Pdlim4
1.61E-18	0.27585009	0.997	0.996	2.53E-14	8	Rpl23
2.24E-18	0.46440931	0.802	0.618	3.51E-14	8	H2afj
2.43E-18	-0.4017005	0.896	0.961	3.80E-14	8	Rbp1
2.63E-18	-0.5319704	0.785	0.914	4.13E-14	8	Mfap5
3.14E-18	0.31844388	0.997	0.996	4.93E-14	8	Rps21
3.49E-18	-0.2879556	1	0.999	5.48E-14	8	Ubb
3.99E-18	-0.681852	0.587	0.752	6.26E-14	8	Ifrd1
5.29E-18	-0.3775098	0.087	0.306	8.29E-14	8	Klf15
5.45E-18	0.46930194	0.583	0.36	8.55E-14	8	500009L16Ril
5.83E-18	0.2891036	1	0.995	9.14E-14	8	Rps15a
8.42E-18	0.50775489	0.618	0.426	1.32E-13	8	Ace
8.94E-18	-0.4722018	0.194	0.423	1.40E-13	8	Asb2
1.14E-17	-0.454368	0.545	0.75	1.78E-13	8	Cav2
1.29E-17	-0.4426722	0.521	0.663	2.02E-13	8	Pink1
1.57E-17	0.28115453	0.997	0.994	2.46E-13	8	Rpl36
1.76E-17	0.51476322	0.302	0.123	2.75E-13	8	Sox4
2.33E-17	0.45959266	0.194	0.046	3.65E-13	8	Cxcl2
2.56E-17	-0.3827776	0.778	0.874	4.02E-13	8	Pdcl3
2.94E-17	0.45823633	0.885	0.787	4.61E-13	8	Htra3
3.02E-17	0.26818408	1	0.999	4.73E-13	8	Rpl18a
3.05E-17	0.25834317	1	0.997	4.78E-13	8	Rpl18
3.16E-17	0.25718641	0.983	0.993	4.95E-13	8	Rpl11
3.51E-17	0.43703905	0.878	0.804	5.51E-13	8	S100a1
5.44E-17	0.34481379	0.986	0.979	8.53E-13	8	Aebp1
6.14E-17	0.36968264	0.972	0.96	9.63E-13	8	Arpc1b
7.15E-17	0.44835339	0.358	0.18	1.12E-12	8	Slc43a3

7.88E-17	-0.3625735	0.955	0.977	1.24E-12	8	Rbpms
9.26E-17	-0.6335832	0.785	0.871	1.45E-12	8	Actg2
9.34E-17	-0.8115092	0.642	0.83	1.46E-12	8	Hspa1a
9.38E-17	0.38877143	0.979	0.959	1.47E-12	8	Rpl22l1
1.14E-16	-0.4518024	0.208	0.43	1.79E-12	8	Rasgrp2
1.26E-16	0.27084991	0.26	0.084	1.97E-12	8	Rarres1
1.27E-16	0.52639048	0.431	0.202	1.99E-12	8	Ptgs2
1.37E-16	0.40406804	0.41	0.199	2.14E-12	8	Nfkbiz
1.69E-16	0.54560117	0.646	0.544	2.64E-12	8	200002D01Ril
2.06E-16	0.41483973	0.941	0.862	3.23E-12	8	Hspg2
2.21E-16	-0.4262597	0.604	0.768	3.46E-12	8	Mbnl2
2.70E-16	-0.4720869	0.448	0.636	4.24E-12	8	Gucy1a1
2.97E-16	-0.449028	0.392	0.598	4.65E-12	8	Tacc2
3.36E-16	-0.4488253	0.378	0.595	5.26E-12	8	Plin4
3.87E-16	0.37849066	0.896	0.778	6.07E-12	8	Vkorc1
4.71E-16	-0.5057557	0.142	0.346	7.39E-12	8	Enpp2
5.33E-16	0.2697817	0.993	0.995	8.36E-12	8	Rpl15
5.86E-16	0.27424843	0.177	0.044	9.19E-12	8	Gch1
6.09E-16	-0.4577866	0.358	0.575	9.54E-12	8	Rassf3
6.49E-16	-0.4478509	0.674	0.804	1.02E-11	8	Capn2
6.51E-16	0.29548462	0.997	0.995	1.02E-11	8	Rps19
1.33E-15	0.44292044	0.747	0.553	2.08E-11	8	Sulf1
1.44E-15	0.37057934	0.906	0.829	2.26E-11	8	Tmem258
1.49E-15	-0.4031855	0.597	0.797	2.33E-11	8	Efhd1
1.88E-15	-0.4077162	0.618	0.759	2.95E-11	8	Rbbp7
1.94E-15	-0.3893178	0.792	0.908	3.05E-11	8	Hacd1
2.11E-15	-0.4318541	0.17	0.371	3.31E-11	8	Otud1
2.15E-15	-0.4217977	0.229	0.447	3.37E-11	8	Gm11627
2.60E-15	-0.3748223	0.118	0.304	4.08E-11	8	Cdkl5
3.00E-15	-0.4064213	0.781	0.88	4.71E-11	8	Eif4a2
3.54E-15	-0.4096407	0.226	0.444	5.55E-11	8	Heyl
4.01E-15	0.25301589	0.236	0.075	6.29E-11	8	Ifitm10
4.22E-15	0.42005615	0.726	0.568	6.62E-11	8	Nbl1
4.24E-15	-0.4308685	0.594	0.762	6.64E-11	8	Anxa11
4.34E-15	-0.3364213	0.885	0.951	6.81E-11	8	Smim14
5.89E-15	-0.4059913	0.24	0.451	9.24E-11	8	Ak3
6.09E-15	0.30011577	0.997	0.985	9.54E-11	8	Rpl22
6.83E-15	-0.4429462	0.375	0.555	1.07E-10	8	Plekho1
7.01E-15	-0.5274146	0.42	0.593	1.10E-10	8	Ppp1r15a
7.43E-15	0.52451046	0.99	0.954	1.17E-10	8	Cfh
8.32E-15	-0.4261805	0.358	0.575	1.30E-10	8	Coro1c
8.43E-15	-0.4245352	0.333	0.526	1.32E-10	8	Hand2
9.68E-15	-0.4432354	0.333	0.534	1.52E-10	8	Pxdc1
9.74E-15	-0.3997778	0.361	0.537	1.53E-10	8	Unc45a
1.04E-14	-0.3406562	0.948	0.967	1.63E-10	8	Bri3
1.07E-14	-0.3999692	0.733	0.843	1.67E-10	8	Myadm
1.07E-14	-0.40139	0.788	0.883	1.68E-10	8	Tln1
1.40E-14	0.38607294	0.774	0.623	2.20E-10	8	Snrpg
1.91E-14	-0.3863499	0.792	0.884	3.00E-10	8	Il11ra1
2.21E-14	0.28706663	0.115	0.024	3.47E-10	8	Cxcl16
2.88E-14	-0.3804442	0.642	0.8	4.51E-10	8	Fam129a

3.04E-14	-0.3931526	0.733	0.82	4.77E-10	8	Kcnmb1
3.32E-14	0.39940481	0.483	0.29	5.21E-10	8	Ptpn2
3.41E-14	0.29251559	0.993	0.994	5.34E-10	8	Rpsa
3.44E-14	0.3622574	0.885	0.825	5.39E-10	8	Tpm4
3.45E-14	0.39115954	0.649	0.476	5.41E-10	8	Fkbp10
3.84E-14	-0.6237905	0.531	0.663	6.02E-10	8	Klf4
3.85E-14	0.39844258	0.653	0.483	6.03E-10	8	Tnfrsf1a
3.86E-14	0.28619006	0.205	0.062	6.06E-10	8	Iigp1
3.87E-14	0.36346289	0.931	0.868	6.07E-10	8	S100a13
4.12E-14	0.31464419	0.292	0.126	6.45E-10	8	Psmb10
4.48E-14	0.410454	0.701	0.531	7.03E-10	8	Wdr89
4.71E-14	-0.4174463	0.757	0.881	7.38E-10	8	Prelp
4.81E-14	0.34538578	0.542	0.321	7.55E-10	8	Ifnar2
5.02E-14	-0.5985611	0.521	0.689	7.88E-10	8	Sgk1
5.60E-14	0.40882688	0.243	0.135	8.78E-10	8	Gbp2
5.85E-14	0.25223714	0.997	0.996	9.17E-10	8	Rps13
7.25E-14	-0.4075872	0.108	0.291	1.14E-09	8	Smim5
1.00E-13	-0.2512944	0.976	0.988	1.57E-09	8	Pls3
1.06E-13	-0.3527693	0.17	0.356	1.67E-09	8	Fyco1
1.18E-13	-0.3856388	0.691	0.807	1.85E-09	8	Svil
1.18E-13	-0.3795974	0.188	0.371	1.86E-09	8	Filip1
1.19E-13	0.3073351	0.965	0.968	1.86E-09	8	Rhoc
1.36E-13	-0.3331348	0.743	0.889	2.13E-09	8	Rcan2
1.38E-13	-0.4153143	0.24	0.428	2.17E-09	8	Cavin2
1.59E-13	-0.2522196	0.031	0.173	2.50E-09	8	Stum
1.94E-13	-0.3786224	0.611	0.751	3.04E-09	8	Emc8
2.25E-13	-0.3038322	0.91	0.96	3.53E-09	8	Sumo2
2.38E-13	-0.7142144	0.354	0.535	3.73E-09	8	Atf3
2.91E-13	0.39189979	0.469	0.285	4.56E-09	8	Chsy1
2.92E-13	-0.3924654	0.448	0.63	4.57E-09	8	Atp2b4
3.90E-13	-0.458642	0.42	0.585	6.12E-09	8	Tes
4.11E-13	-0.3573029	0.719	0.835	6.44E-09	8	Degs1
4.13E-13	0.36380891	0.528	0.327	6.48E-09	8	Scn1b
4.46E-13	-0.3887062	0.951	0.974	7.00E-09	8	Lmna
4.78E-13	-0.3407283	0.934	0.978	7.49E-09	8	Jund
4.91E-13	0.37719348	0.667	0.537	7.69E-09	8	Imp3
5.49E-13	0.51541897	0.684	0.686	8.62E-09	8	Sod2
5.60E-13	-0.5111364	0.306	0.473	8.78E-09	8	Atf5
5.78E-13	-0.3982387	0.097	0.261	9.06E-09	8	Adm
6.58E-13	0.36629983	0.566	0.361	1.03E-08	8	Eif1a
6.59E-13	0.97555804	0.906	0.797	1.03E-08	8	Gm42418
7.02E-13	-0.2976655	0.927	0.975	1.10E-08	8	Capns1
7.04E-13	0.4553597	0.413	0.221	1.10E-08	8	Phlda1
7.07E-13	-0.3823013	0.576	0.721	1.11E-08	8	Ccni
7.64E-13	0.36270204	0.497	0.29	1.20E-08	8	Mmp14
8.45E-13	-0.3828084	0.434	0.603	1.33E-08	8	Acadm
1.02E-12	-0.3790257	0.33	0.53	1.60E-08	8	Pgm5
1.05E-12	-0.3982741	0.382	0.573	1.65E-08	8	Hspa2
1.14E-12	-0.2932781	0.938	0.972	1.78E-08	8	Eif4g2
1.19E-12	-0.3485005	0.833	0.917	1.86E-08	8	7-Sep
1.43E-12	-0.2994922	0.938	0.971	2.25E-08	8	Atp5b

1.84E-12	0.35483821	0.729	0.663	2.89E-08	8	Nme1
1.91E-12	-0.3702283	0.472	0.626	3.00E-08	8	Gna11
2.04E-12	-0.3361786	0.823	0.918	3.19E-08	8	Mpc1
2.08E-12	-0.3395828	0.385	0.517	3.27E-08	8	Fez2
2.16E-12	0.39024798	0.51	0.32	3.38E-08	8	Fosl2
2.92E-12	-0.6469528	0.399	0.556	4.58E-08	8	Flnc
3.32E-12	0.27147546	0.997	0.992	5.20E-08	8	Rpl17
3.39E-12	0.34141855	0.889	0.816	5.31E-08	8	Itm2c
3.46E-12	-0.3688204	0.576	0.725	5.43E-08	8	Fkbp4
3.56E-12	-0.3078987	0.927	0.964	5.59E-08	8	Lamp1
3.96E-12	-0.3742168	0.462	0.631	6.21E-08	8	Idh2
5.43E-12	-0.3595213	0.444	0.621	8.52E-08	8	Hebp1
5.46E-12	-0.295361	0.156	0.291	8.57E-08	8	Rab3a
5.84E-12	-0.3456531	0.347	0.488	9.15E-08	8	Cap2
5.86E-12	-0.3811298	0.594	0.727	9.19E-08	8	Dusp3
6.17E-12	0.30037679	0.979	0.95	9.67E-08	8	Usmg5
6.29E-12	-0.2820671	0.997	0.996	9.86E-08	8	Hsp90ab1
6.53E-12	-0.3041829	0.899	0.965	1.02E-07	8	Hspb7
6.98E-12	0.33055113	0.913	0.895	1.10E-07	8	Rrbp1
7.81E-12	0.28069467	0.285	0.122	1.22E-07	8	C1s1
8.32E-12	0.27405104	0.465	0.264	1.30E-07	8	Qpct
9.13E-12	-0.3579176	0.861	0.943	1.43E-07	8	Oat
1.15E-11	-0.3157258	0.875	0.948	1.81E-07	8	Hmgn1
1.21E-11	0.39732995	0.771	0.63	1.90E-07	8	Col6a3
1.22E-11	-0.5351666	0.198	0.387	1.91E-07	8	Cdkn1a
1.37E-11	-0.3597757	0.493	0.648	2.14E-07	8	Rbpms2
1.39E-11	-0.3879752	0.479	0.627	2.18E-07	8	Scube3
1.43E-11	-0.3156001	0.299	0.44	2.25E-07	8	Aktip
1.47E-11	-0.4183603	0.771	0.872	2.30E-07	8	Dnajb4
1.71E-11	-0.5176714	0.656	0.769	2.68E-07	8	Jun
1.95E-11	-0.3561385	0.677	0.786	3.05E-07	8	Csde1
2.03E-11	-0.2511616	0.024	0.145	3.19E-07	8	Bmp3
2.27E-11	0.32968885	0.941	0.845	3.56E-07	8	Fn1
2.35E-11	-0.3187412	0.792	0.882	3.69E-07	8	Cyb5a
2.37E-11	-0.3536463	0.056	0.196	3.72E-07	8	Igfbp5
2.44E-11	0.40007871	0.67	0.515	3.83E-07	8	Sulf2
2.91E-11	0.3984997	0.323	0.159	4.56E-07	8	Wisp2
3.23E-11	-0.3520285	0.524	0.637	5.06E-07	8	Tmem43
3.27E-11	-0.2821405	0.924	0.95	5.12E-07	8	Aplp2
3.51E-11	0.25683415	0.205	0.075	5.51E-07	8	Dclk1
3.62E-11	0.27843418	0.389	0.205	5.68E-07	8	B4galt1
3.80E-11	0.27674362	0.299	0.138	5.96E-07	8	Man2a1
4.01E-11	-0.339637	0.819	0.898	6.29E-07	8	Atp2a2
4.77E-11	-0.2816	0.156	0.291	7.48E-07	8	Pygb
4.90E-11	-0.31825	0.84	0.915	7.69E-07	8	Nptn
5.32E-11	-0.4574845	0.472	0.617	8.35E-07	8	Hsph1
5.34E-11	-0.3768107	0.212	0.391	8.37E-07	8	Epas1
5.49E-11	-0.359073	0.622	0.755	8.61E-07	8	Tax1bp1
5.67E-11	-0.3525147	0.177	0.331	8.89E-07	8	Tgfb3
5.81E-11	-0.3002384	0.868	0.931	9.11E-07	8	Cnbp
6.09E-11	-0.2936451	0.851	0.904	9.55E-07	8	Mlf2

6.17E-11	-0.3694749	0.51	0.63	9.68E-07	8	Npr1
6.17E-11	-0.2693906	0.024	0.134	9.68E-07	8	Slc22a1
6.47E-11	-0.3455827	0.67	0.772	1.01E-06	8	Kank1
1.04E-10	0.33885953	0.521	0.344	1.63E-06	8	Stat3
1.10E-10	-0.356144	0.705	0.811	1.72E-06	8	Cltb
1.15E-10	0.32948531	0.507	0.325	1.81E-06	8	Tgfb1
1.22E-10	-0.3601333	0.694	0.798	1.92E-06	8	Ccdc107
1.23E-10	0.3578661	0.562	0.419	1.93E-06	8	Ank
1.37E-10	0.27638493	0.306	0.147	2.14E-06	8	Matn2
1.37E-10	0.29779466	0.497	0.302	2.15E-06	8	Neat1
1.48E-10	0.25153262	0.295	0.138	2.32E-06	8	Layn
1.52E-10	0.30606665	0.316	0.178	2.38E-06	8	Shroom3
1.63E-10	-0.3278264	0.84	0.909	2.56E-06	8	Ywhah
1.69E-10	0.25983342	0.934	0.917	2.65E-06	8	Ndufa1
1.76E-10	-0.2850215	0.122	0.277	2.77E-06	8	Pygm
2.05E-10	-0.3298297	0.51	0.647	3.22E-06	8	Polr2m
2.27E-10	0.38559027	0.656	0.507	3.56E-06	8	Lmo4
2.88E-10	-0.3371057	0.753	0.839	4.52E-06	8	Rras
2.93E-10	0.32978658	0.604	0.452	4.59E-06	8	Kdelr3
2.93E-10	0.34964602	0.799	0.698	4.60E-06	8	Mxra8
3.24E-10	-0.3859491	0.323	0.496	5.08E-06	8	Itgbl1
3.42E-10	-0.2638874	0.059	0.192	5.36E-06	8	F3
3.58E-10	0.25463923	0.962	0.951	5.61E-06	8	Npm1
3.75E-10	-0.3422764	0.667	0.783	5.89E-06	8	Gstm2
3.78E-10	0.28708311	0.91	0.87	5.92E-06	8	Atox1
4.27E-10	-0.3136017	0.698	0.788	6.69E-06	8	Hmgn3
4.34E-10	-0.338724	0.486	0.647	6.81E-06	8	Chpt1
4.55E-10	0.27241331	0.222	0.089	7.14E-06	8	Sphk1
4.95E-10	-0.3457614	0.358	0.512	7.77E-06	8	Dtna
5.98E-10	-0.2945739	0.299	0.42	9.38E-06	8	Msra
6.21E-10	0.36704049	0.701	0.564	9.74E-06	8	Fat1
6.40E-10	0.35198912	0.757	0.644	1.00E-05	8	Col4a5
6.57E-10	-0.3367837	0.243	0.398	1.03E-05	8	Dsp
7.11E-10	-0.3181314	0.281	0.425	1.11E-05	8	Tspan5
7.70E-10	0.32185288	0.576	0.386	1.21E-05	8	Atp1b1
8.05E-10	0.3240925	0.472	0.291	1.26E-05	8	Adam9
8.34E-10	-0.3145683	0.632	0.76	1.31E-05	8	Mrpl42
8.72E-10	-0.290755	0.156	0.296	1.37E-05	8	Pnck
1.22E-09	-0.2753122	0.833	0.937	1.91E-05	8	Lmcd1
1.26E-09	-0.3078044	0.219	0.369	1.98E-05	8	Dhrs3
1.30E-09	0.31419949	0.497	0.324	2.04E-05	8	Pet100
1.38E-09	0.25414234	0.25	0.115	2.17E-05	8	Fkbp11
1.38E-09	-0.3138728	0.212	0.374	2.17E-05	8	Anln
1.40E-09	-0.3382955	0.257	0.397	2.20E-05	8	Glul
1.81E-09	0.36365353	0.823	0.728	2.84E-05	8	Pdgfa
1.86E-09	0.27115496	0.882	0.826	2.92E-05	8	Prdx2
2.02E-09	0.26848895	0.924	0.895	3.17E-05	8	Tma7
2.19E-09	-0.2977663	0.656	0.757	3.44E-05	8	Dctn6
2.20E-09	-0.3515029	0.396	0.542	3.45E-05	8	Tob2
2.33E-09	-0.3270708	0.403	0.539	3.65E-05	8	Asap2
2.34E-09	-0.2996119	0.247	0.39	3.67E-05	8	Ppp2r5a

2.49E-09	-0.2932946	0.535	0.629	3.91E-05	8	Foxn3
2.53E-09	-0.3298925	0.535	0.649	3.97E-05	8	Gnai1
2.54E-09	0.26327297	0.906	0.87	3.98E-05	8	Ndufa3
2.58E-09	0.30585623	0.726	0.618	4.05E-05	8	H13
2.62E-09	0.3084341	0.917	0.86	4.11E-05	8	Atp5k
3.08E-09	-0.2764697	0.236	0.367	4.84E-05	8	Chmp4c
3.10E-09	-0.3066594	0.448	0.594	4.86E-05	8	Smpdl3a
3.31E-09	0.29099608	0.806	0.702	5.19E-05	8	Ier3ip1
3.39E-09	0.32984962	0.542	0.388	5.31E-05	8	Pdgfrb
3.55E-09	-0.2879458	0.389	0.543	5.56E-05	8	Efr3a
3.89E-09	-0.2518086	0.892	0.922	6.10E-05	8	Ndufb10
3.98E-09	-0.2833278	0.281	0.419	6.23E-05	8	Rilpl1
4.01E-09	0.28031584	0.438	0.268	6.28E-05	8	Rps12
4.32E-09	-0.2986437	0.26	0.405	6.78E-05	8	Dact3
4.49E-09	-0.3172215	0.472	0.623	7.04E-05	8	Pacsin2
4.84E-09	-0.2972574	0.378	0.523	7.59E-05	8	Zc2hc1a
4.95E-09	-0.3101816	0.653	0.768	7.76E-05	8	Ppp1r2
5.41E-09	0.25498034	0.924	0.928	8.48E-05	8	Pdia3
5.41E-09	0.3018555	0.809	0.72	8.49E-05	8	Cnpy2
5.79E-09	-0.2738412	0.222	0.349	9.08E-05	8	Prdm6
6.41E-09	0.27236052	0.271	0.136	0.00010059	8	Hrct1
6.66E-09	-0.3120063	0.872	0.934	0.00010439	8	Pdlim1
7.78E-09	-0.2914271	0.132	0.272	0.00012204	8	Map7d2
8.42E-09	0.26352555	0.719	0.544	0.0001321	8	Sdf2
8.52E-09	-0.2880419	0.75	0.858	0.00013354	8	Cfl2
8.81E-09	0.33197489	0.493	0.326	0.00013811	8	Angpt2
9.87E-09	-0.301986	0.681	0.783	0.00015473	8	Mapre1
1.06E-08	-0.2809818	0.799	0.85	0.00016641	8	Bnip2
1.13E-08	-0.2875868	0.285	0.441	0.00017669	8	Prkar2a
1.14E-08	-0.3078873	0.646	0.769	0.00017857	8	Aoc3
1.20E-08	-0.2933203	0.733	0.837	0.00018779	8	Raly
1.21E-08	-0.2905006	0.545	0.672	0.00019013	8	Vapb
1.23E-08	-0.2608843	0.247	0.363	0.00019255	8	Rnf167
1.38E-08	-0.2886298	0.441	0.565	0.00021649	8	Camta1
1.47E-08	-0.2920556	0.451	0.585	0.00023001	8	Uba2
1.52E-08	0.28564613	0.51	0.339	0.00023861	8	Spcs3
1.58E-08	-0.3112016	0.528	0.668	0.00024793	8	Rnf10
1.61E-08	0.28978885	0.705	0.556	0.00025274	8	Lamp2
1.63E-08	-0.2983068	0.292	0.431	0.00025493	8	Dnajb5
1.77E-08	0.31879748	0.597	0.459	0.00027686	8	Alcam
1.80E-08	-0.2807867	0.347	0.506	0.00028261	8	Lims1
1.81E-08	0.32481203	0.653	0.508	0.00028419	8	Fads3
1.87E-08	-0.3164127	0.253	0.397	0.00029302	8	Peli1
1.88E-08	-0.2974237	0.604	0.71	0.00029462	8	Tmem109
1.88E-08	-0.2849048	0.448	0.574	0.00029478	8	Wbp2
1.88E-08	-0.2993898	0.33	0.482	0.00029546	8	Gamt
1.92E-08	0.25577438	0.382	0.223	0.00030173	8	Ano1
2.00E-08	0.3014538	0.59	0.428	0.00031325	8	P4ha1
2.07E-08	0.25230884	0.174	0.099	0.00032468	8	Rnd1
2.21E-08	-0.2943949	0.59	0.677	0.00034604	8	Smc6
2.22E-08	-0.3036934	0.455	0.552	0.00034795	8	Ehd4

2.27E-08	-0.3181858	0.41	0.495	0.0003553	8	Ccnl1
2.29E-08	-0.3027395	0.247	0.376	0.00035939	8	Stk17b
2.33E-08	-0.3455472	0.406	0.515	0.0003648	8	Bag3
2.43E-08	0.31207482	0.809	0.695	0.00038037	8	Mmp23
2.48E-08	0.27062803	0.84	0.789	0.00038914	8	Snrpe
2.66E-08	-0.3168681	0.295	0.463	0.00041701	8	Abi3bp
2.75E-08	-0.3555575	0.236	0.36	0.0004309	8	Pcolce2
2.76E-08	-0.2840383	0.611	0.75	0.0004321	8	Nox4
2.81E-08	-0.2705291	0.361	0.446	0.00044065	8	Acadsb
3.04E-08	-0.287308	0.646	0.747	0.00047615	8	Slc48a1
3.34E-08	-0.2758894	0.167	0.319	0.00052367	8	Cdkn1c
3.36E-08	-0.2948588	0.351	0.525	0.00052762	8	Crispld2
3.50E-08	-0.2791327	0.34	0.457	0.00054814	8	Pcmtd1
3.50E-08	-0.2964883	0.462	0.567	0.00054855	8	Arid4b
3.70E-08	0.25800384	0.278	0.171	0.00057982	8	Nfkb1
3.75E-08	-0.2918931	0.767	0.833	0.0005874	8	Ehd2
3.83E-08	-0.3027742	0.333	0.481	0.0006009	8	Myom1
4.13E-08	-0.2700569	0.198	0.347	0.00064751	8	Bcar3
4.15E-08	-0.4339082	0.816	0.901	0.00065079	8	Dnaja1
4.21E-08	0.41724333	0.951	0.939	0.00065985	8	Pam
4.29E-08	-0.3027762	0.503	0.648	0.0006721	8	Rhoj
4.37E-08	-0.2862251	0.337	0.488	0.0006848	8	Camk2g
4.49E-08	-0.2847436	0.642	0.735	0.00070338	8	Map7d1
5.13E-08	-1.5252065	0.201	0.088	0.00080401	8	Hbb-bs
5.36E-08	-0.2577811	0.497	0.582	0.00084104	8	Golgb1
5.63E-08	-0.303643	0.326	0.463	0.00088307	8	Lama4
5.85E-08	0.26448902	0.424	0.267	0.00091717	8	Rpl36a-ps1
5.89E-08	-0.2865545	0.615	0.713	0.00092432	8	Ptov1
5.95E-08	-0.2798875	0.562	0.679	0.00093235	8	Ahsa1
5.95E-08	-0.2875215	0.562	0.674	0.00093331	8	Tspan2
6.74E-08	-0.2648822	0.188	0.324	0.0010564	8	Mettl24
6.93E-08	0.32931233	0.361	0.215	0.00108691	8	Notch2
7.06E-08	-0.3916466	0.618	0.731	0.00110739	8	Adamts1
7.85E-08	0.27558672	0.406	0.255	0.00123053	8	Mpp6
8.19E-08	0.25819638	0.337	0.19	0.00128363	8	Rcan1
9.11E-08	-0.2512816	0.142	0.273	0.00142828	8	Mknk2
9.12E-08	0.27553946	0.34	0.217	0.00143011	8	Pik3r1
9.13E-08	0.28262286	0.618	0.464	0.00143094	8	Fuca1
9.44E-08	-0.2707063	0.292	0.416	0.00148102	8	Cds2
9.58E-08	0.25846358	0.778	0.64	0.00150213	8	Rpl10-ps3
9.74E-08	0.25666328	0.486	0.321	0.00152767	8	Ell2
1.00E-07	-0.2841184	0.351	0.504	0.00157472	8	Klf7
1.11E-07	0.29298688	0.483	0.342	0.00174501	8	Nedd9
1.21E-07	0.26896707	0.719	0.599	0.00190498	8	Erp29
1.29E-07	-0.2872469	0.503	0.624	0.0020224	8	Pttg1ip
1.35E-07	-0.2924926	0.649	0.736	0.00211941	8	Ebf1
1.36E-07	-0.2601846	0.729	0.829	0.00213218	8	Vdac2
1.46E-07	0.27933762	0.559	0.446	0.00228584	8	Flot1
1.58E-07	0.26985665	0.795	0.725	0.00248488	8	Ctsh
1.69E-07	-0.2719502	0.486	0.604	0.00265451	8	Dynlt3
1.81E-07	0.275312	0.694	0.589	0.00283773	8	Psme2

1.87E-07	-0.2678298	0.503	0.592	0.00293714	8	Zfr
2.04E-07	0.29997669	0.674	0.562	0.00319168	8	H2-K1
2.08E-07	-0.2618816	0.59	0.689	0.00326273	8	2-Mar
2.08E-07	0.27774877	0.823	0.758	0.00326567	8	Hspb2
2.12E-07	-0.2579203	0.392	0.529	0.00332496	8	Bod1l
2.23E-07	0.26983694	0.719	0.645	0.00349097	8	Eif4ebp1
2.34E-07	-0.2519756	0.858	0.909	0.00366935	8	Lamb2
2.37E-07	-0.263777	0.312	0.439	0.00371671	8	Cystm1
2.38E-07	-0.2613726	0.861	0.919	0.00372586	8	Rheb
2.46E-07	-0.2515072	0.347	0.47	0.00385351	8	Raf1
2.54E-07	-0.2573755	0.677	0.743	0.00397983	8	Ctnna1
2.60E-07	-0.3450432	0.851	0.932	0.00408392	8	Tnfrsf12a
2.72E-07	0.29035894	0.74	0.622	0.00426143	8	Kdelr2
2.79E-07	-0.2688471	0.385	0.511	0.00437559	8	Kank2
2.82E-07	-0.2574572	0.34	0.495	0.0044217	8	Lzts2
3.13E-07	-0.2604517	0.632	0.727	0.0049086	8	Nudc
3.19E-07	-0.2692657	0.469	0.602	0.00500235	8	Lgr6
3.41E-07	-0.2656401	0.747	0.834	0.00534636	8	Ube2d2a
3.64E-07	0.2839293	0.816	0.726	0.0057094	8	Lrp1
3.83E-07	-0.2535271	0.344	0.442	0.00599808	8	Thoc2
3.87E-07	0.27365266	0.469	0.313	0.00607252	8	Polr2l
4.00E-07	0.25901106	0.448	0.296	0.00626793	8	Syt11
4.64E-07	-0.2520551	0.583	0.667	0.00728361	8	Mrvi1
4.66E-07	0.25357827	0.497	0.346	0.00730305	8	Capg
4.79E-07	-0.2696231	0.323	0.466	0.0075144	8	Ldb3
4.80E-07	-0.2701155	0.59	0.698	0.00751956	8	Jph2
4.95E-07	-0.2518951	0.128	0.259	0.00776213	8	Ptprz1
5.11E-07	0.25698689	0.705	0.593	0.00801037	8	Tmem167
5.28E-07	-0.2816145	0.358	0.48	0.00828021	8	Map1a
5.63E-07	-0.2500102	0.788	0.859	0.0088336	8	Klf9
5.90E-07	-0.2696729	0.278	0.395	0.00924581	8	Rhobtb1
6.12E-07	0.29793657	0.531	0.408	0.00960366	8	Eif4b
6.25E-07	-0.2544361	0.41	0.505	0.00980707	8	Atp6v1a
6.74E-07	-0.2606059	0.597	0.692	0.0105714	8	Ppp2r1a
7.35E-07	0.26271472	0.448	0.299	0.011521	8	Ctsc
7.51E-07	-0.2514564	0.417	0.527	0.01178424	8	Nr3c1
7.74E-07	-0.2578145	0.66	0.744	0.01213242	8	Ift43
8.20E-07	-0.2617261	0.403	0.54	0.01286084	8	C1qtnf2
8.58E-07	0.25696344	0.712	0.595	0.01345715	8	Shisa5
8.88E-07	0.28507236	0.694	0.556	0.01391744	8	Scarb2
9.24E-07	-0.2803976	0.229	0.356	0.01448869	8	Rgs7bp
9.97E-07	0.25061199	0.556	0.424	0.01563709	8	Snhg6
1.09E-06	0.25745614	0.938	0.911	0.01712642	8	Snhg18
1.16E-06	-0.2556146	0.802	0.869	0.01817101	8	Srsf3
1.20E-06	-0.3595382	0.934	0.975	0.01877161	8	Cryab
1.20E-06	0.2785971	0.826	0.738	0.01879603	8	Fkbp2
1.21E-06	-0.3463157	0.483	0.587	0.01899414	8	Gem
1.61E-06	-0.4794061	0.469	0.584	0.02529663	8	Fosb
1.62E-06	-0.2562544	0.653	0.735	0.0254809	8	Cltc
2.11E-06	-0.2692493	0.5	0.593	0.03315707	8	Bex3
2.14E-06	0.2507522	0.701	0.558	0.03354681	8	Bola2

2.16E-06	-0.2906329	0.576	0.667	0.03380642	8	Hspb8
2.20E-06	-0.2582508	0.354	0.472	0.03455653	8	Syne2
2.43E-06	-0.2892004	0.451	0.541	0.03805904	8	Emb
2.49E-06	-0.3930823	0.347	0.424	0.03900238	8	Ier5
2.58E-06	-0.2524848	0.726	0.829	0.04042491	8	Susd5
3.23E-06	-0.2634878	0.51	0.624	0.05061482	8	Klf13
3.51E-06	-0.2546903	0.892	0.936	0.05507234	8	Actn4
3.67E-06	0.25669915	0.611	0.51	0.05753216	8	Tmem208
4.56E-06	0.27945568	0.83	0.75	0.07152635	8	Olfml2b
4.81E-06	-0.2901239	0.535	0.625	0.07540471	8	Dkk3
5.31E-06	-0.2713281	0.434	0.538	0.08328289	8	Cdh6
5.33E-06	0.25783811	0.74	0.616	0.08355669	8	Timm10b
5.57E-06	-0.2525767	0.712	0.79	0.08733925	8	Enah
6.59E-06	-0.2514811	0.392	0.519	0.10328009	8	Itga1
6.87E-06	-0.2666147	0.646	0.748	0.10765764	8	Abracl
6.97E-06	-0.2766781	0.604	0.693	0.10935792	8	Anxa3
8.76E-06	0.27218665	0.615	0.555	0.13737367	8	Tuba1c
1.20E-05	-0.2770755	0.295	0.343	0.18839145	8	Pecam1
1.24E-05	-0.2706298	0.632	0.721	0.19453282	8	Sf3b1
1.83E-05	0.35187526	0.656	0.606	0.28680657	8	Inhba
1.84E-05	-0.2793027	0.597	0.629	0.28830166	8	Igfbp4
2.29E-05	0.27310928	0.583	0.476	0.35967752	8	Igf1r
2.32E-05	-0.3785229	0.347	0.475	0.36458267	8	Dnajb1
3.05E-05	-0.3210457	0.483	0.579	0.47760192	8	Zfp36
3.10E-05	-0.282537	0.455	0.559	0.48615505	8	Bambi
3.53E-05	-0.3169454	0.726	0.767	0.552967	8	Ier2
4.83E-05	-0.2780002	0.99	0.993	0.75698556	8	Hspb1
6.63E-05	-0.2787101	0.767	0.805	1	8	Klf6
6.93E-05	-0.4225829	0.889	0.929	1	8	S100a10
0.00013536	-0.3354164	0.25	0.214	1	8	Emp1
0.00014003	-0.5013534	0.601	0.602	1	8	Hbegf
0.00044745	-0.4448517	0.399	0.461	1	8	Errfi1
0.00186778	-0.2812126	0.75	0.776	1	8	Egr1
0.0027285	-0.3130815	0.875	0.889	1	8	Junb
0.00724212	0.34539739	0.656	0.718	1	8	Nfkbia

Table S2. List of highly expressed genes identified in cluster 7(avg_logFC > 1)

	p_val	avg_logF(	pct.1	pct.2	p_val_adj	cluster	gene
Cd74	0	4.564421	0.924	0.052	0	7	Cd74
Lyz2	0	4.162743	0.838	0.032	0	7	Lyz2
H2-Ab1	0	3.90765	0.644	0.048	0	7	H2-Ab1
H2-Aa	0	3.847654	0.61	0.033	0	7	H2-Aa
H2-Eb1	0	3.613524	0.581	0.028	0	7	H2-Eb1
Ctss	0	3.332134	0.952	0.014	0	7	Ctss
Plac8	0	3.216873	0.692	0.011	0	7	Plac8
Fcer1g	0	2.733033	0.949	0.035	0	7	Fcer1g
Tyrobp	0	2.593041	0.937	0.039	0	7	Tyrobp
Tgfbi	0	2.580494	0.778	0.017	0	7	Tgfbi
Cd52	0	2.560113	0.902	0.034	0	7	Cd52
Il1b	0	2.48661	0.905	0.053	0	7	Il1b
Coro1a	0	2.359004	0.879	0.031	0	7	Coro1a
Chil3	0	2.355081	0.378	0.004	0	7	Chil3
Cxcl2	0	2.145657	0.778	0.094	0	7	Cxcl2
Ifi27l2a	0	2.14507	0.603	0.037	0	7	Ifi27l2a
Laptm5	0	2.113533	0.892	0.024	0	7	Laptm5
Ccl9	0	2.097486	0.695	0.011	0	7	Ccl9
Cd14	0	1.997675	0.686	0.039	0	7	Cd14
Slfn2	0	1.974967	0.711	0.023	0	7	Slfn2
Ms4a6c	0	1.951614	0.705	0.003	0	7	Ms4a6c
Cotl1	0	1.934528	0.819	0.033	0	7	Cotl1
Wfdc17	0	1.918123	0.721	0.018	0	7	Wfdc17
Lst1	0	1.914648	0.752	0.021	0	7	Lst1
Ucp2	0	1.874481	0.825	0.017	0	7	Ucp2
Srgn1	0	1.866034	0.933	0.146	0	7	Srgn
Alox5ap	0	1.859582	0.749	0.051	0	7	Alox5ap
Fcgr2b	0	1.859369	0.66	0.004	0	7	Fcgr2b
Tnf	0	1.849619	0.727	0.025	0	7	Tnf
H2-DMa	0	1.843581	0.654	0.011	0	7	H2-DMa
Ifitm1	0	1.762803	0.486	0.026	0	7	Ifitm1
Cybb	0	1.737547	0.641	0.004	0	7	Cybb
Cytip	0	1.722293	0.743	0.016	0	7	Cytip
Bcl2a1b	0	1.721792	0.673	0.02	0	7	Bcl2a1b
Plek	0	1.708417	0.794	0.033	0	7	Plek
Napsa	0	1.707454	0.67	0.006	0	7	Napsa
Arhgdib1	0	1.707057	0.778	0.084	0	7	Arhgdib
Ptprc	0	1.705641	0.778	0.021	0	7	Ptprc
Rel	0	1.681937	0.724	0.053	0	7	Rel
Mpeg1	0	1.665269	0.676	0.006	0	7	Mpeg1
Ccl4	0	1.657583	0.432	0.017	0	7	Ccl4
Ccl6	0	1.652378	0.613	0.018	0	7	Ccl6
Ccr2	0	1.640533	0.59	0.003	0	7	Ccr2
Cd53	0	1.637559	0.79	0.025	0	7	Cd53
Aif1	0	1.625964	0.562	0.003	0	7	Aif1
Tlr2	0	1.615268	0.61	0.05	0	7	Tlr2
Csf1r	0	1.611881	0.651	0.006	0	7	Csf1r
Spi1	0	1.611017	0.743	0.019	0	7	Spi1
Tnfaip3	0	1.609874	0.74	0.046	0	7	Tnfaip3
Clec4e	0	1.581351	0.603	0.024	0	7	Clec4e
Cd68	0	1.568767	0.673	0.024	0	7	Cd68
Ms4a6d	0	1.562999	0.546	0.003	0	7	Ms4a6d

Fcgr3	0	1.555681	0.587	0.014	0	7	Fcgr3
Lcp1	0	1.526747	0.781	0.025	0	7	Lcp1
Plbd1	0	1.523907	0.67	0.009	0	7	Plbd1
Ms4a4c	0	1.515901	0.476	0.002	0	7	Ms4a4c
Ifi207	0	1.50081	0.578	0.002	0	7	Ifi207
Dusp2	0	1.481846	0.657	0.032	0	7	Dusp2
Pla2g7	0	1.460269	0.498	0.013	0	7	Pla2g7
Ly86	0	1.427512	0.644	0.002	0	7	Ly86
Il1rn	0	1.409636	0.527	0.022	0	7	Il1rn
Ifitm6	0	1.409472	0.467	0.007	0	7	Ifitm6
Unc93b1	0	1.398317	0.689	0.043	0	7	Unc93b1
Itgb2	0	1.382528	0.632	0.014	0	7	Itgb2
Ptpn18	0	1.380096	0.651	0.03	0	7	Ptpn18
Cd83	0	1.360799	0.521	0.002	0	7	Cd83
Clec4n	0	1.329286	0.508	0.014	0	7	Clec4n
Emilin2	0	1.319788	0.571	0.014	0	7	Emilin2
C5ar1	0	1.314482	0.524	0.022	0	7	C5ar1
Nlrp3	0	1.306675	0.657	0.024	0	7	Nlrp3
Lyn	0	1.304255	0.651	0.016	0	7	Lyn
Cfp	0	1.303218	0.537	0.014	0	7	Cfp
Gmfg	0	1.289706	0.711	0.024	0	7	Gmfg
Slc7a11	0	1.279944	0.552	0.023	0	7	Slc7a11
Lilr4b	0	1.278015	0.622	0.016	0	7	Lilr4b
Ptafr	0	1.263997	0.584	0.017	0	7	Ptafr
Clec4a3	0	1.260819	0.533	0.001	0	7	Clec4a3
Ncf2	0	1.259449	0.616	0.044	0	7	Ncf2
Pld4	0	1.25863	0.571	0.002	0	7	Pld4
Ms4a6b	0	1.238352	0.483	0.003	0	7	Ms4a6b
Pirb	0	1.225504	0.587	0.01	0	7	Pirb
Cd300c2	0	1.220853	0.549	0.007	0	7	Cd300c2
F10	0	1.207454	0.394	0.002	0	7	F10
Clec4d	0	1.199311	0.575	0.028	0	7	Clec4d
Gm6377	0	1.19709	0.463	0.006	0	7	Gm6377
Fyb	0	1.181356	0.587	0.034	0	7	Fyb
Hcls1	0	1.164891	0.641	0.046	0	7	Hcls1
Ifi204	0	1.146451	0.448	0.002	0	7	Ifi204
Ptpn6	0	1.142312	0.581	0.011	0	7	Ptpn6
Ly6c2	0	1.139393	0.263	0.003	0	7	Ly6c2
Sirpb1c	0	1.121961	0.483	0.01	0	7	Sirpb1c
Rac2	0	1.121428	0.625	0.022	0	7	Rac2
Samsn1	0	1.105023	0.492	0.021	0	7	Samsn1
Cxcr4	0	1.099485	0.457	0.014	0	7	Cxcr4
Cyp4f18	0	1.075538	0.511	0.012	0	7	Cyp4f18
Selplg	0	1.059458	0.61	0.022	0	7	Selplg
Osm	0	1.055886	0.368	0.006	0	7	Osm
Ncf4	0	1.053945	0.537	0.011	0	7	Ncf4
Traf1	0	1.046141	0.479	0.021	0	7	Traf1
Csf2rb	0	1.03546	0.521	0.007	0	7	Csf2rb
Gpr132	0	1.027228	0.498	0.007	0	7	Gpr132
Nrros	0	1.025026	0.492	0.007	0	7	Nrros
Hck	0	1.020016	0.508	0.005	0	7	Hck
Nfkbid	0	1.012017	0.552	0.019	0	7	Nfkbid
Marcksl1	1.61E-305	1.707961	0.737	0.086	2.53E-301	7	Marcksl1
Gngt21	7.37E-300	1.431588	0.695	0.072	1.16E-295	7	Gngt2

Ccl3	3.65E-299	1.088773	0.438	0.021	5.72E-295	7	Ccl3
Rab20	4.87E-290	1.169797	0.495	0.033	7.64E-286	7	Rab20
Marcks	5.18E-290	1.327856	0.552	0.044	8.13E-286	7	Marcks
Hp	2.68E-287	1.430248	0.394	0.017	4.20E-283	7	Hp
Psmb81	2.91E-284	1.487352	0.721	0.089	4.57E-280	7	Psmb8
Il6	2.00E-280	1.142897	0.235	0.003	3.13E-276	7	Il6
C1qb	2.28E-278	1.715079	0.197	0.001	3.58E-274	7	C1qb
Fam49b	2.84E-277	1.695979	0.829	0.148	4.46E-273	7	Fam49b
Rgs1	2.66E-268	1.048253	0.241	0.004	4.17E-264	7	Rgs1
Ccl2	3.58E-268	1.259207	0.235	0.004	5.62E-264	7	Ccl2
Hmox1	9.94E-265	2.286701	0.537	0.048	1.56E-260	7	Hmox1
H2-DMb1	9.55E-251	1.558918	0.505	0.045	1.50E-246	7	H2-DMb1
C1qa	1.09E-249	1.639237	0.181	0.001	1.71E-245	7	C1qa
Rnf149	9.43E-249	1.483296	0.743	0.117	1.48E-244	7	Rnf149
Trf	7.04E-245	1.390383	0.511	0.046	1.10E-240	7	Trf
C1qc	4.59E-243	1.261347	0.168	0.001	7.20E-239	7	C1qc
Sirpa	6.57E-234	1.165975	0.559	0.061	1.03E-229	7	Sirpa
Tgif1	2.91E-228	1.290578	0.654	0.094	4.56E-224	7	Tgif1
Ly6e1	1.33E-222	1.570491	0.781	0.128	2.09E-218	7	Ly6e
Pim1	2.71E-214	1.89545	0.895	0.255	4.25E-210	7	Pim1
Gch1	6.56E-197	1.070171	0.537	0.069	1.03E-192	7	Gch1
Cxcl10	4.00E-185	1.239243	0.254	0.011	6.27E-181	7	Cxcl10
Samhd1	3.78E-175	1.441433	0.673	0.138	5.93E-171	7	Samhd1
Ftl11	2.11E-174	2.102977	0.997	0.978	3.32E-170	7	Ftl1
Tnfsf9	9.18E-170	1.352762	0.448	0.055	1.44E-165	7	Tnfsf9
Slpi	1.37E-168	1.172806	0.308	0.021	2.15E-164	7	Slpi
Baz1a	9.54E-165	1.032003	0.594	0.107	1.50E-160	7	Baz1a
Mafb	2.22E-163	1.307329	0.438	0.054	3.48E-159	7	Mafb
Fau1	1.47E-156	1.028845	0.997	0.994	2.30E-152	7	Fau
Cd44	1.54E-152	1.534036	0.737	0.204	2.41E-148	7	Cd44
Nfkbiz	1.74E-150	1.579517	0.756	0.226	2.73E-146	7	Nfkbiz
Cxcl161	2.25E-149	1.266163	0.46	0.066	3.54E-145	7	Cxcl16
Ctsc	4.38E-140	1.780773	0.765	0.273	6.87E-136	7	Ctsc
Pf4	1.61E-138	1.785179	0.149	0.004	2.53E-134	7	Pf4
Cdkn1a1	1.14E-136	1.637562	0.848	0.381	1.78E-132	7	Cdkn1a
Tmem176a1	1.88E-135	1.223512	0.489	0.081	2.95E-131	7	Tmem176a
Litaf	5.62E-132	1.014151	0.705	0.198	8.81E-128	7	Litaf
Sh3bgrl31	1.34E-129	1.625943	0.908	0.714	2.10E-125	7	Sh3bgrl3
Nfkbia	4.47E-129	1.856798	0.921	0.725	7.01E-125	7	Nfkbia
Tpd52	5.91E-129	1.268085	0.743	0.251	9.27E-125	7	Tpd52
Lsp1	1.20E-128	1.632949	0.641	0.177	1.88E-124	7	Lsp1
Atox11	6.85E-128	1.46481	0.933	0.855	1.07E-123	7	Atox1
Psap	1.16E-126	1.633612	0.975	0.942	1.82E-122	7	Psap
Bhlhe401	3.29E-125	1.27021	0.565	0.128	5.16E-121	7	Bhlhe40
B2m1	3.75E-125	1.153814	0.968	0.978	5.89E-121	7	B2m
Cyba	1.22E-123	1.568565	0.902	0.827	1.91E-119	7	Cyba
Kdm6b	1.85E-121	1.274512	0.673	0.201	2.90E-117	7	Kdm6b
Ccr7	3.64E-119	1.380946	0.124	0.003	5.71E-115	7	Ccr7
Prkcd	1.04E-114	1.271863	0.695	0.232	1.63E-110	7	Prkcd
Lgals31	8.58E-112	1.826207	0.876	0.597	1.35E-107	7	Lgals3
Hmgb2	5.71E-110	1.025712	0.603	0.165	8.96E-106	7	Hmgb2
Btg1	2.97E-109	1.603683	0.879	0.65	4.66E-105	7	Btg1
H2-K11	3.67E-109	1.297178	0.898	0.594	5.76E-105	7	H2-K1
Rilpl2	2.43E-107	1.112957	0.606	0.178	3.81E-103	7	Rilpl2

Mcl1	3.36E-105	1.386181	0.857	0.505	5.27E-101	7	Mcl1
Npc21	7.99E-104	1.418496	0.87	0.699	1.25E-99	7	Npc2
Tmem176b1	1.20E-102	1.307492	0.549	0.137	1.88E-98	7	Tmem176b
Ccl5	3.57E-101	1.57719	0.121	0.004	5.61E-97	7	Ccl5
Thbs1	1.60E-100	1.64727	0.508	0.118	2.52E-96	7	Thbs1
Gm2a	6.62E-98	1.419598	0.746	0.36	1.04E-93	7	Gm2a
H2-D11	1.26E-97	1.232587	0.911	0.856	1.98E-93	7	H2-D1
Efhd2	1.51E-96	1.174668	0.721	0.311	2.37E-92	7	Efhd2
H2afz	3.12E-96	1.465955	0.873	0.806	4.89E-92	7	H2afz
Csrnp11	2.75E-94	1.142426	0.711	0.306	4.31E-90	7	Csrnp1
Ccrl2	2.43E-93	1.207712	0.546	0.157	3.82E-89	7	Ccrl2
Plaur1	2.53E-93	1.427487	0.695	0.287	3.97E-89	7	Plaur
Junb1	5.62E-92	1.03874	0.927	0.881	8.82E-88	7	Junb
Ptpn1	1.19E-91	1.149949	0.648	0.244	1.87E-87	7	Ptpn1
Tmsb101	2.04E-89	1.398399	0.895	0.853	3.19E-85	7	Tmsb10
Gsr	1.46E-86	1.268353	0.67	0.282	2.29E-82	7	Gsr
Arpc5	4.56E-84	1.061619	0.825	0.585	7.15E-80	7	Arpc5
Wsb11	4.01E-82	1.143094	0.683	0.296	6.28E-78	7	Wsb1
Zeb2	1.20E-81	1.101606	0.581	0.202	1.88E-77	7	Zeb2
Ier51	1.41E-81	1.110859	0.784	0.416	2.20E-77	7	Ier5
Ctsz	1.60E-81	1.197897	0.819	0.66	2.51E-77	7	Ctsz
Plin2	1.65E-79	1.046601	0.625	0.24	2.59E-75	7	Plin2
Rap1b	6.01E-71	1.044526	0.794	0.593	9.43E-67	7	Rap1b
Fxyd52	4.78E-69	1.230776	0.787	0.546	7.50E-65	7	Fxyd5
Tgfb1	6.20E-68	1.135515	0.654	0.335	9.72E-64	7	Tgfb1
Ifitm31	2.68E-61	1.33997	0.879	0.924	4.20E-57	7	Ifitm3
Zfp361	9.85E-61	1.05354	0.8	0.586	1.54E-56	7	Zfp36
Nfe2l2	2.29E-58	1.116672	0.705	0.436	3.59E-54	7	Nfe2l2
Phlda11	1.61E-54	1.058975	0.527	0.221	2.52E-50	7	Phlda1
Ier31	1.18E-53	1.43556	0.756	0.584	1.85E-49	7	Ier3
Ifitm21	8.94E-51	1.035684	0.851	0.954	1.40E-46	7	Ifitm2
Prdx5	6.28E-44	1.060742	0.806	0.805	9.85E-40	7	Prdx5
Lgmn	9.05E-37	1.209484	0.746	0.64	1.42E-32	7	Lgmn
Ptgs21	9.44E-35	1.365045	0.451	0.21	1.48E-30	7	Ptgs2
Cstb	2.18E-32	1.019964	0.787	0.799	3.42E-28	7	Cstb
Fabp51	4.14E-22	1.601393	0.324	0.153	6.49E-18	7	Fabp5
Syngr2	1.31E-21	1.076498	0.603	0.523	2.06E-17	7	Syngr2
Apoe1	1.80E-21	1.358503	0.733	0.753	2.83E-17	7	Apoe
Cxcl11	3.56E-15	1.291491	0.216	0.094	5.59E-11	7	Cxcl1

Table S3. List of highly expressed genes identified in cluster 10(avg_logFC > 1)

	p_val	avg_logFC	pct.1	pct.2	p_val_adj	cluster	gene
S100a8	0	5.9489239	0.844	0.028	0	10	S100a8
S100a9	0	5.8256082	0.873	0.027	0	10	S100a9
Cxcl21	0	4.3268882	0.994	0.103	0	10	Cxcl2
Il1b1	0	3.8528582	1	0.068	0	10	Il1b
Retnlg	0	3.8434836	0.41	0.002	0	10	Retnlg
G0s2	0	3.68903	0.665	0.005	0	10	G0s2
Ccl31	0	3.6271245	0.63	0.025	0	10	Ccl3
Gm5483	0	3.3700698	0.382	0.002	0	10	Gm5483
Slpi1	0	3.2933175	0.613	0.019	0	10	Slpi
Il1r21	0	3.2886971	0.913	0.022	0	10	Il1r2
Ifitm11	0	3.2881967	0.775	0.029	0	10	Ifitm1
Clec4d1	0	3.2003124	0.855	0.032	0	10	Clec4d
Tnf1	0	3.1336373	0.751	0.038	0	10	Tnf
Tyrobp1	0	3.1206636	0.988	0.055	0	10	Tyrobp
Acod1	0	3.0937702	0.705	0.007	0	10	Acod1
Clec4e1	0	2.9343961	0.821	0.03	0	10	Clec4e
Nlrp31	0	2.8978464	0.861	0.031	0	10	Nlrp3
Il1rn1	0	2.8752499	0.763	0.026	0	10	Il1rn
Hdc	0	2.7945329	0.746	0.006	0	10	Hdc
Fcer1g1	0	2.7905598	0.948	0.053	0	10	Fcer1g
Hcar2	0	2.7753319	0.647	0.007	0	10	Hcar2
Plek1	0	2.7743962	0.855	0.047	0	10	Plek
Slc7a111	0	2.6780128	0.751	0.028	0	10	Slc7a11
Trem11	0	2.6717343	0.728	0.02	0	10	Trem1
Cd521	0	2.5972262	0.873	0.051	0	10	Cd52
Stfa2l1	0	2.588776	0.382	0.001	0	10	Stfa2l1
Bcl2a1b1	0	2.5380027	0.647	0.033	0	10	Bcl2a1b
Asprv1	0	2.5187	0.462	0.002	0	10	Asprv1
C5ar11	0	2.4736131	0.769	0.026	0	10	C5ar1
AA4671971	0	2.4544562	0.462	0.011	0	10	AA467197
Wfdc21	0	2.4375627	0.428	0.003	0	10	Wfdc21
Samsn11	0	2.4358626	0.723	0.025	0	10	Samsn1
Lcp11	0	2.4242207	0.786	0.04	0	10	Lcp1
Csf3r	0	2.3571056	0.676	0.008	0	10	Csf3r
Ccr11	0	2.3523656	0.711	0.021	0	10	Ccr1
Gmfg1	0	2.3498073	0.682	0.038	0	10	Gmfg
Hp1	0	2.3414442	0.543	0.021	0	10	Hp
Il1f9	0	2.2813655	0.457	0.001	0	10	Il1f9
Mmp9	0	2.2781264	0.671	0.006	0	10	Mmp9
Cxcr2	0	2.2210955	0.595	0.001	0	10	Cxcr2
Arg21	0	2.2101275	0.566	0.017	0	10	Arg2
Ptafr1	0	2.1255948	0.618	0.027	0	10	Ptafr
Coro1a1	0	2.1221711	0.792	0.05	0	10	Coro1a
Pglyrp1	0	2.0925855	0.503	0.016	0	10	Pglyrp1
Rac21	0	2.0788945	0.659	0.033	0	10	Rac2
Spi11	0	2.0164254	0.665	0.035	0	10	Spi1
F63002801	0	1.9749846	0.439	0.004	0	10	F630028O10Rik
Cd300lf1	0	1.875265	0.566	0.011	0	10	Cd300lf
Slfn1	0	1.7498547	0.405	0.006	0	10	Slfn1
H2-Q10	0	1.6525569	0.41	0.004	0	10	H2-Q10
Fpr1	0	1.5490509	0.295	0.003	0	10	Fpr1
Cd331	0	1.493363	0.416	0.011	0	10	Cd33

AC110211.	0	1.4001281	0.324	0.001	0	10	AC110211.1
Mrgpra2b	0	1.1929111	0.214	0	0	10	Mrgpra2b
Sirpb1b1	4.81E-301	1.5154283	0.445	0.014	7.55E-297	10	Sirpb1b
Clec5a1	9.78E-299	1.4917315	0.376	0.009	1.53E-294	10	Clec5a
Upp1	2.39E-297	1.7161323	0.376	0.009	3.75E-293	10	Upp1
Cd531	3.40E-294	2.1675042	0.682	0.042	5.33E-290	10	Cd53
Slfn4	1.18E-292	1.0525261	0.22	0.001	1.85E-288	10	Slfn4
Gm19951	4.99E-290	1.5909545	0.272	0.003	7.83E-286	10	Gm19951
Cd141	1.51E-289	3.180256	0.723	0.051	2.37E-285	10	Cd14
Pilra1	2.78E-284	1.6704619	0.48	0.018	4.35E-280	10	Pilra
Il23a1	6.93E-282	1.9897606	0.341	0.007	1.09E-277	10	Il23a
Lst11	2.55E-275	2.1179608	0.636	0.038	3.99E-271	10	Lst1
Alox5ap1	1.52E-273	2.5430304	0.769	0.064	2.38E-269	10	Alox5ap
Ltb	6.48E-271	1.6319002	0.335	0.007	1.02E-266	10	Ltb
Marcksl11	1.27E-269	3.2457652	0.884	0.095	1.99E-265	10	Marcksl1
Selplg1	3.68E-257	2.1237175	0.578	0.035	5.76E-253	10	Selplg
Igsf61	9.17E-250	1.6643027	0.468	0.021	1.44E-245	10	Igsf6
Lilr4b1	1.07E-248	2.0346188	0.538	0.03	1.68E-244	10	Lilr4b
Ptprc1	1.12E-247	1.9593947	0.613	0.04	1.75E-243	10	Ptprc
Snx201	4.78E-243	1.720396	0.48	0.023	7.49E-239	10	Snx20
Slfn21	5.23E-241	1.9888656	0.601	0.04	8.20E-237	10	Slfn2
Il1a1	1.02E-237	1.9655312	0.387	0.014	1.61E-233	10	Il1a
Cd300ld1	5.60E-237	1.3907846	0.341	0.01	8.78E-233	10	Cd300ld
Srgn3	7.10E-237	3.4536641	0.988	0.16	1.11E-232	10	Srgn
Lmnb11	1.02E-234	2.4007356	0.642	0.051	1.59E-230	10	Lmnb1
Ccl61	1.07E-223	2.8906026	0.52	0.032	1.68E-219	10	Ccl6
Nfkbid1	2.64E-221	1.8498119	0.509	0.031	4.13E-217	10	Nfkbid
Laptm51	7.69E-211	1.6355412	0.613	0.047	1.21E-206	10	Laptm5
Cxcl31	2.14E-207	2.0458732	0.26	0.006	3.35E-203	10	Cxcl3
Emilin21	5.88E-203	1.4868894	0.468	0.028	9.22E-199	10	Emilin2
Cyp4f181	1.08E-202	1.5813841	0.439	0.024	1.69E-198	10	Cyp4f18
Sell1	2.94E-201	1.2897629	0.335	0.012	4.61E-197	10	Sell
Trem31	2.25E-200	1.3023453	0.312	0.01	3.53E-196	10	Trem3
Wfdc171	3.03E-196	3.0845272	0.514	0.036	4.74E-192	10	Wfdc17
Fcgr31	1.83E-195	1.7329265	0.462	0.028	2.87E-191	10	Fcgr3
Fpr21	1.96E-195	1.1976407	0.283	0.008	3.08E-191	10	Fpr2
Cotl11	5.45E-195	1.759713	0.613	0.054	8.55E-191	10	Cotl1
Clec4n1	1.21E-189	2.2530144	0.434	0.025	1.89E-185	10	Clec4n
Siglece	2.20E-189	1.0658289	0.277	0.008	3.45E-185	10	Siglece
Itgam1	1.22E-188	1.4554004	0.405	0.022	1.91E-184	10	Itgam
Ccl41	6.98E-183	3.5155996	0.422	0.025	1.09E-178	10	Ccl4
Lilra6	5.34E-182	1.0139426	0.22	0.005	8.37E-178	10	Lilra6
Sirpb1c1	6.18E-182	1.3585274	0.399	0.022	9.69E-178	10	Sirpb1c
1600010M(	3.54E-176	1.1097448	0.237	0.006	5.55E-172	10	1600010M07Rik
Mcemp11	1.61E-175	1.5689842	0.376	0.02	2.52E-171	10	Mcemp1
Tnfaip21	4.31E-174	2.1483185	0.514	0.043	6.76E-170	10	Tnfaip2
Gm26740	6.27E-173	1.0609829	0.254	0.008	9.83E-169	10	Gm26740
Pilrb11	1.56E-172	1.0243379	0.272	0.009	2.44E-168	10	Pilrb1
Mxd11	2.92E-172	2.2294354	0.647	0.077	4.58E-168	10	Mxd1
Pla2g71	4.04E-172	1.501445	0.41	0.025	6.33E-168	10	Pla2g7
Rdh12	1.11E-169	1.3966696	0.277	0.01	1.74E-165	10	Rdh12
Bcl2a1a1	1.14E-169	1.7129607	0.358	0.019	1.79E-165	10	Bcl2a1a
Jaml1	7.92E-168	1.39365	0.283	0.011	1.24E-163	10	Jaml
Nfam11	1.15E-167	1.2752849	0.306	0.013	1.81E-163	10	Nfam1

Hcst1	1.25E-167	1.2252884	0.329	0.016	1.96E-163	10	Hcst
Gpr841	1.78E-166	1.3823367	0.266	0.009	2.80E-162	10	Gpr84
Mir142hg	2.12E-163	1.0430662	0.202	0.004	3.33E-159	10	Mir142hg
Dusp21	5.26E-163	2.4618706	0.52	0.047	8.26E-159	10	Dusp2
Cxcr41	2.93E-160	1.8052543	0.387	0.024	4.60E-156	10	Cxcr4
Itgb21	3.69E-157	1.3801119	0.428	0.031	5.78E-153	10	Itgb2
Retreg11	2.12E-155	1.2831889	0.335	0.018	3.33E-151	10	Retreg1
Gm43936	6.79E-153	1.2032896	0.156	0.002	1.06E-148	10	Gm43936
Lrrc251	3.65E-151	1.4394034	0.364	0.023	5.73E-147	10	Lrrc25
Rnf1491	3.19E-145	2.2342919	0.74	0.129	5.00E-141	10	Rnf149
Ltb4r11	1.49E-138	1.3034045	0.295	0.016	2.33E-134	10	Ltb4r1
Ncf21	4.02E-138	1.7095788	0.52	0.058	6.31E-134	10	Ncf2
Gpsm31	1.83E-137	1.4780166	0.451	0.042	2.87E-133	10	Gpsm3
Gm51501	1.09E-134	1.1783055	0.283	0.015	1.71E-130	10	Gm5150
Mmp8	2.35E-131	1.0305801	0.179	0.005	3.68E-127	10	Mmp8
Tnfaip31	1.26E-128	1.7345724	0.532	0.065	1.97E-124	10	Tnfaip3
Ncf41	2.72E-128	1.1678361	0.353	0.026	4.27E-124	10	Ncf4
2310001H1	6.77E-128	1.486993	0.382	0.031	1.06E-123	10	2310001H17Rik
Lcn21	1.56E-127	1.9974942	0.312	0.02	2.45E-123	10	Lcn2
Neurl31	1.28E-119	1.3754466	0.318	0.022	2.01E-115	10	Neurl3
Fmnl11	4.69E-119	1.2383971	0.324	0.023	7.35E-115	10	Fmnl1
Ezr1	2.28E-116	1.5761626	0.422	0.044	3.57E-112	10	Ezr
Lpcat21	7.55E-115	1.2702744	0.37	0.033	1.18E-110	10	Lpcat2
Rab11fip1	4.10E-113	1.2293712	0.277	0.017	6.43E-109	10	Rab11fip1
Ncf11	8.76E-112	1.1297478	0.266	0.016	1.37E-107	10	Ncf1
Cd371	2.60E-111	1.0211086	0.237	0.012	4.08E-107	10	Cd37
Hcls11	4.00E-110	1.5350993	0.48	0.062	6.27E-106	10	Hcls1
Itgal1	2.13E-108	1.1288597	0.266	0.017	3.35E-104	10	Itgal
Adam81	5.75E-107	1.1851829	0.249	0.015	9.02E-103	10	Adam8
Cd441	1.04E-106	2.3102961	0.78	0.214	1.63E-102	10	Cd44
Nfkbia1	1.50E-106	3.0205918	0.988	0.727	2.35E-102	10	Nfkbia
Pirb1	2.70E-105	1.1198486	0.329	0.028	4.24E-101	10	Pirb
AB1246111	8.90E-104	1.2568584	0.312	0.025	1.40E-99	10	AB124611
Pim11	1.35E-103	2.6199951	0.832	0.269	2.12E-99	10	Pim1
Stx111	1.87E-103	1.4492339	0.405	0.046	2.93E-99	10	Stx11
Syk1	7.59E-103	1.0526645	0.277	0.02	1.19E-98	10	Syk
Ptpn61	4.66E-101	1.2810781	0.324	0.028	7.31E-97	10	Ptpn6
Fgr1	7.92E-97	1.1425957	0.289	0.023	1.24E-92	10	Fgr
Cd24a2	4.42E-96	1.7083135	0.595	0.106	6.93E-92	10	Cd24a
Gda3	1.20E-90	1.3970663	0.382	0.045	1.88E-86	10	Gda
Dok31	1.85E-89	1.0539145	0.237	0.016	2.91E-85	10	Dok3
Ftl13	1.48E-87	1.8544272	0.988	0.978	2.33E-83	10	Ftl1
Cytip1	4.38E-87	1.2069768	0.358	0.04	6.86E-83	10	Cytip
Lyn1	1.07E-86	1.3340835	0.335	0.036	1.67E-82	10	Lyn
Nfkbiz1	1.49E-84	2.1882443	0.734	0.237	2.33E-80	10	Nfkbiz
Dgat21	6.27E-83	1.1724013	0.26	0.022	9.84E-79	10	Dgat2
Cd801	6.50E-82	1.1651555	0.22	0.015	1.02E-77	10	Cd80
Marcks1	2.39E-81	1.3880661	0.405	0.058	3.75E-77	10	Marcks
Pygl1	4.40E-81	1.344272	0.376	0.05	6.90E-77	10	Pygl
Cd300a1	1.18E-80	1.1305683	0.254	0.021	1.85E-76	10	Cd300a
Ccrl21	1.07E-77	2.6803429	0.618	0.163	1.67E-73	10	Ccrl2
Plaur2	5.19E-77	2.2827026	0.763	0.293	8.13E-73	10	Plaur
Klf21	5.19E-76	2.1426226	0.919	0.592	8.14E-72	10	Klf2
Ifitm23	2.36E-75	1.6179113	0.948	0.95	3.69E-71	10	Ifitm2

Bcl2a1d1	8.54E-75	1.1368899	0.243	0.021	1.34E-70	10 Bcl2a1d
Slc16a31	5.92E-74	2.0144638	0.59	0.155	9.28E-70	10 Slc16a3
Rab201	6.30E-73	1.2982258	0.347	0.046	9.88E-69	10 Rab20
Btg11	6.71E-73	2.1098661	0.89	0.655	1.05E-68	10 Btg1
Junb3	7.98E-73	1.7087974	0.954	0.881	1.25E-68	10 Junb
Gsr1	5.00E-72	2.0514426	0.734	0.288	7.84E-68	10 Gsr
Mcl11	2.59E-71	2.104752	0.85	0.513	4.06E-67	10 Mcl1
Cks21	1.78E-70	2.0237496	0.474	0.096	2.79E-66	10 Cks2
Arhgdib3	1.66E-69	1.4557287	0.503	0.105	2.61E-65	10 Arhgdib
Lrg12	3.11E-69	2.0534372	0.353	0.051	4.88E-65	10 Lrg1
Il1rap	1.51E-67	1.015176	0.277	0.032	2.36E-63	10 Il1rap
Litaf2	1.94E-67	1.7857559	0.647	0.209	3.04E-63	10 Litaf
Fth11	6.55E-67	1.337634	1	0.999	1.03E-62	10 Fth1
Igfbp61	1.21E-66	1.2199657	0.208	0.017	1.90E-62	10 Igfbp6
Fxyd54	1.24E-66	1.7148816	0.867	0.549	1.95E-62	10 Fxyd5
Ifitm61	6.83E-66	1.2078515	0.231	0.021	1.07E-61	10 Ifitm6
Apbb1ip1	4.94E-63	1.3383386	0.318	0.045	7.75E-59	10 Apbb1ip
Bst12	2.71E-59	1.0093811	0.26	0.031	4.25E-55	10 Bst1
Trib11	7.41E-57	1.6752912	0.514	0.142	1.16E-52	10 Trib1
S100a111	5.13E-55	1.2887879	0.954	0.986	8.05E-51	10 S100a11
Lfng1	7.50E-55	1.0476735	0.243	0.03	1.18E-50	10 Lfng
Chd71	1.02E-54	1.0742207	0.249	0.031	1.60E-50	10 Chd7
Samhd11	2.09E-54	1.5409301	0.526	0.152	3.27E-50	10 Samhd1
Vsir1	1.24E-52	1.1633492	0.272	0.039	1.94E-48	10 Vsir
Icam13	1.60E-52	1.7798681	0.434	0.101	2.51E-48	10 Icam1
Lsp11	5.26E-52	1.6571917	0.566	0.188	8.25E-48	10 Lsp1
Fam107b2	5.34E-50	1.3240643	0.353	0.07	8.37E-46	10 Fam107b
Adrb21	1.22E-49	1.0326211	0.191	0.02	1.91E-45	10 Adrb2
Ier33	1.89E-49	2.2900315	0.809	0.587	2.97E-45	10 Ier3
Pnrc11	2.11E-49	1.8798694	0.786	0.597	3.31E-45	10 Pnrc1
H3f3b2	4.47E-48	1.0847743	0.931	0.978	7.01E-44	10 H3f3b
Smox1	4.80E-48	1.279346	0.329	0.063	7.52E-44	10 Smox
Gm140051	5.65E-48	1.0887659	0.225	0.029	8.86E-44	10 Gm14005
Pmaip11	6.61E-47	1.1754506	0.179	0.018	1.04E-42	10 Pmaip1
Ier52	1.76E-46	1.6193666	0.728	0.424	2.77E-42	10 Ier5
Tpd521	3.02E-45	1.7385785	0.607	0.263	4.73E-41	10 Tpd52
Msrb1	2.26E-41	1.3853081	0.855	0.852	3.54E-37	10 Msrb1
Ets21	5.74E-41	1.6612538	0.543	0.219	9.00E-37	10 Ets2
Vasp1	1.07E-40	1.4989037	0.78	0.703	1.67E-36	10 Vasp
Traf11	4.61E-40	1.0579818	0.231	0.036	7.23E-36	10 Traf1
281047401	4.94E-37	2.0660514	0.607	0.317	7.75E-33	10 2810474019Rik
Osm1	1.50E-36	1.0496284	0.156	0.018	2.35E-32	10 Osm
Prdx51	5.28E-36	1.5753373	0.775	0.806	8.28E-32	10 Prdx5
H2-D13	8.51E-35	1.2594676	0.827	0.86	1.33E-30	10 H2-D1
Srsf51	1.63E-33	1.4044962	0.734	0.654	2.55E-29	10 Srsf5
Btg21	5.02E-33	1.2164151	0.821	0.793	7.87E-29	10 Btg2
Zfp363	9.67E-33	1.6944523	0.728	0.592	1.52E-28	10 Zfp36
Fam49b1	4.13E-32	1.2245575	0.457	0.17	6.47E-28	10 Fam49b
Cdk2ap21	4.47E-31	1.9245076	0.717	0.662	7.01E-27	10 Cdk2ap2
Rap1b1	4.49E-31	1.3558659	0.711	0.599	7.03E-27	10 Rap1b
Rel1	4.54E-30	1.133687	0.301	0.077	7.11E-26	10 Rel
Gadd45a2	1.10E-29	1.7326518	0.405	0.145	1.73E-25	10 Gadd45a
Tlr21	1.29E-29	1.1383221	0.283	0.069	2.03E-25	10 Tlr2
Sh3bgrl13	5.37E-29	1.2824854	0.746	0.721	8.42E-25	10 Sh3bgrl3

Grina	5.77E-29	1.9470496	0.561	0.321	9.04E-25	10	Grina
Kctd122	2.71E-27	1.0850566	0.318	0.092	4.26E-23	10	Kctd12
Rhog1	2.99E-27	1.2629357	0.451	0.196	4.69E-23	10	Rhog
Tgif11	5.71E-27	1.3293953	0.347	0.112	8.95E-23	10	Tgif1
Glipr2	7.63E-24	1.123791	0.329	0.114	1.20E-19	10	Glipr2
Dennd4a2	1.35E-23	1.179908	0.347	0.124	2.12E-19	10	Dennd4a
Arrdc4	2.80E-23	1.0564228	0.249	0.067	4.39E-19	10	Arrdc4
Rgcc2	3.33E-23	1.5039004	0.353	0.131	5.21E-19	10	Rgcc
N4bp11	5.58E-23	1.193463	0.306	0.101	8.75E-19	10	N4bp1
Arpc31	3.33E-22	1.1310675	0.717	0.831	5.23E-18	10	Arpc3
Kdm6b1	7.91E-22	1.3578169	0.445	0.216	1.24E-17	10	Kdm6b
Ostf11	2.74E-21	1.3064408	0.671	0.672	4.30E-17	10	Ostf1
Hmgb21	2.96E-21	1.3321409	0.399	0.179	4.65E-17	10	Hmgb2
Nfe2l21	5.26E-21	1.3997027	0.59	0.444	8.26E-17	10	Nfe2l2
Tsc22d32	1.61E-20	1.2443464	0.301	0.105	2.53E-16	10	Tsc22d3
Csrnp12	5.41E-19	1.4159162	0.503	0.319	8.48E-15	10	Csrnp1
Cyba1	3.69E-18	1.0223695	0.705	0.833	5.78E-14	10	Cyba
Stk17b1	5.71E-18	1.3823024	0.509	0.345	8.95E-14	10	Stk17b
Cebpb1	1.63E-17	1.1348293	0.295	0.112	2.55E-13	10	Cebpb
Plin21	1.96E-17	1.4891021	0.439	0.252	3.08E-13	10	Plin2
Alas11	1.18E-16	1.0835907	0.341	0.156	1.85E-12	10	Alas1
Thbs13	1.64E-15	1.3279959	0.318	0.13	2.58E-11	10	Thbs1
Taldo11	1.67E-15	1.4553712	0.636	0.734	2.63E-11	10	Taldo1
Ccnl12	1.95E-15	1.3014058	0.566	0.498	3.06E-11	10	Ccnl1
Wsb12	2.28E-14	1.2097284	0.462	0.309	3.58E-10	10	Wsb1
Irf13	3.55E-14	1.2840152	0.277	0.116	5.57E-10	10	Irf1
Ppp1r15a4	4.00E-14	1.2574119	0.618	0.604	6.27E-10	10	Ppp1r15a
Gm26532	6.53E-14	1.071116	0.243	0.093	1.02E-09	10	Gm26532
Klf62	1.50E-13	1.1926619	0.671	0.778	2.34E-09	10	Klf6
Gngt23	5.16E-13	1.1164082	0.243	0.095	8.10E-09	10	Gngt2
Gadd45b2	7.19E-13	1.2847139	0.63	0.695	1.13E-08	10	Gadd45b
Ninj13	7.56E-13	1.1939684	0.59	0.607	1.19E-08	10	Ninj1
Zcchc61	2.54E-12	1.1611128	0.376	0.226	3.98E-08	10	Zcchc6
Cdkn1a2	1.05E-11	1.422764	0.503	0.399	1.65E-07	10	Cdkn1a
Ptgs22	1.40E-11	1.5336604	0.37	0.217	2.19E-07	10	Ptgs2
Card191	1.93E-11	1.1111681	0.618	0.714	3.03E-07	10	Card19
Bcl101	2.58E-11	1.05769	0.382	0.245	4.04E-07	10	Bcl10
Arpc51	2.62E-10	1.0330339	0.566	0.596	4.11E-06	10	Arpc5
Socs34	1.12E-09	1.2787914	0.439	0.336	1.76E-05	10	Socs3
Ptbp32	1.18E-09	1.1108573	0.329	0.202	1.85E-05	10	Ptbp3
Gcnt21	4.06E-09	1.1080119	0.277	0.153	6.37E-05	10	Gcnt2
Txnip2	4.39E-09	1.1592279	0.486	0.443	6.89E-05	10	Txnip
Cox171	3.23E-08	1.1670718	0.566	0.697	0.000506	10	Cox17
Prr133	4.68E-08	1.2292646	0.474	0.447	0.0007345	10	Prr13
Rilpl21	5.80E-08	1.1631539	0.306	0.193	0.0009094	10	Rilpl2
Ehd12	6.71E-08	1.0751038	0.497	0.491	0.0010529	10	Ehd1
Sde21	1.52E-07	1.0338765	0.353	0.253	0.0023842	10	Sde2
Mar-71	2.18E-07	1.0235096	0.312	0.208	0.0034192	10	7-Mar
Adipor11	5.64E-07	1.0556473	0.526	0.605	0.0088363	10	Adipor1
Phlda12	8.34E-07	1.2276915	0.329	0.232	0.0130754	10	Phlda1
Adgre5	1.39E-06	1.2682739	0.509	0.572	0.0217685	10	Adgre5
Supt4a1	2.20E-06	1.013061	0.434	0.414	0.0344745	10	Supt4a
Cstb1	0.0003419	1.0316432	0.572	0.804	1	10	Cstb
Hilpda1	0.0018713	1.2687713	0.191	0.133	1	10	Hilpda

Table S4. List of highly expressed genes identified in cluster 12(avg_logFC > 1)

	p_val	avg_logF(	pct.1	pct.2	p_val_adj	cluster	gene
Trbc2	0	3.168417	0.844	0.003	0	12	Trbc2
Ms4a4b	0	2.301644	0.688	0.003	0	12	Ms4a4b
Cd3d	0	2.272156	0.781	0	0	12	Cd3d
Cd3g	0	2.106663	0.656	0	0	12	Cd3g
Trbc1	0	2.095181	0.5	0	0	12	Trbc1
Ptprcap	0	2.002018	0.688	0.003	0	12	Ptprcap
Nkg7	0	1.966468	0.312	0	0	12	Nkg7
Cd2	0	1.936378	0.719	0.001	0	12	Cd2
Trac	0	1.751069	0.625	0.001	0	12	Trac
Lat	0	1.71811	0.594	0	0	12	Lat
Il2rb	0	1.687773	0.594	0.002	0	12	Il2rb
Cd3e	0	1.550554	0.625	0	0	12	Cd3e
Gimap3	0	1.53362	0.594	0	0	12	Gimap3
Lck	0	1.390671	0.625	0.001	0	12	Lck
Gm8369	0	1.310283	0.469	0.001	0	12	Gm8369
Cd28	0	1.305977	0.375	0	0	12	Cd28
Skap1	0	1.283133	0.5	0.001	0	12	Skap1
Cd247	0	1.159322	0.5	0.003	0	12	Cd247
Icos	0	1.139869	0.312	0	0	12	Icos
Sh2d2a	0	1.100808	0.375	0	0	12	Sh2d2a
Tcf7	0	1.095387	0.438	0.002	0	12	Tcf7
Cd27	0	1.091952	0.438	0	0	12	Cd27
Ctsw	0	1.003182	0.281	0	0	12	Ctsw
Klra1	4.63E-252	1.015792	0.156	0	7.27E-248	12	Klra1
Tnfrsf18	2.50E-245	1.456263	0.438	0.004	3.92E-241	12	Tnfrsf18
Ltb1	6.81E-237	2.072778	0.719	0.012	1.07E-232	12	Ltb
Ptpn22	8.78E-176	1.48295	0.406	0.005	1.38E-171	12	Ptpn22
Il7r	3.42E-168	1.442682	0.344	0.003	5.37E-164	12	Il7r
Ccr71	9.27E-159	1.253351	0.438	0.006	1.45E-154	12	Ccr7
Cd7	6.46E-156	1.167888	0.312	0.003	1.01E-151	12	Cd7
Thy1	4.49E-154	1.392469	0.406	0.006	7.04E-150	12	Thy1
P2ry101	2.02E-153	1.164823	0.406	0.006	3.17E-149	12	P2ry10
Gimap61	1.68E-147	2.066341	0.812	0.028	2.63E-143	12	Gimap6
Hcst2	3.26E-142	1.66582	0.688	0.02	5.11E-138	12	Hcst
Cd372	2.27E-137	1.462502	0.594	0.015	3.56E-133	12	Cd37
Gimap51	2.39E-132	1.329072	0.594	0.016	3.76E-128	12	Gimap5
Rhoh1	3.57E-130	1.152762	0.375	0.006	5.60E-126	12	Rhoh
Cytip2	7.69E-117	1.661456	0.906	0.044	1.21E-112	12	Cytip
Igkc	1.19E-115	2.753929	0.125	0	1.86E-111	12	Igkc
Ly6d	1.19E-115	1.422083	0.125	0	1.86E-111	12	Ly6d
Gm267401	3.01E-112	1.367577	0.469	0.011	4.73E-108	12	Gm26740
Rac22	8.86E-111	2.210673	0.875	0.044	1.39E-106	12	Rac2
Gimap42	7.63E-108	1.829597	0.656	0.025	1.20E-103	12	Gimap4
Rgs11	1.87E-106	1.354369	0.469	0.012	2.93E-102	12	Rgs1
Iglc2	2.73E-101	1.834226	0.125	0.001	4.28E-97	12	Iglc2
Cd532	4.23E-97	2.141775	0.906	0.054	6.64E-93	12	Cd53
Ikzf11	4.98E-97	1.048081	0.438	0.012	7.81E-93	12	Ikzf1
Ramp31	2.22E-96	1.925202	0.406	0.01	3.48E-92	12	Ramp3
Ighm1	2.10E-95	1.879344	0.375	0.009	3.29E-91	12	Ighm
Klrk1	2.88E-94	1.024096	0.281	0.005	4.52E-90	12	Klrk1
Tnfrsf9	5.66E-92	1.008657	0.281	0.005	8.88E-88	12	Tnfrsf9
Coro1a2	7.98E-91	1.962103	0.969	0.063	1.25E-86	12	Coro1a

Ccl51	6.80E-90	2.997566	0.344	0.008	1.07E-85	12 Ccl5
Cotl12	2.89E-83	1.913403	0.906	0.063	4.53E-79	12 Cotl1
Il2rg1	8.31E-81	1.340462	0.594	0.028	1.30E-76	12 Il2rg
B4galnt11	2.67E-78	1.260141	0.469	0.017	4.19E-74	12 B4galnt1
Laptm52	5.78E-76	1.550913	0.844	0.057	9.07E-72	12 Laptm5
Ptpn182	8.28E-76	1.809765	0.781	0.053	1.30E-71	12 Ptpn18
Gimap12	7.82E-75	1.447053	0.594	0.03	1.23E-70	12 Gimap1
Klrd11	7.04E-72	1.318106	0.312	0.008	1.10E-67	12 Klrd1
Cd522	4.90E-69	1.921598	0.875	0.067	7.68E-65	12 Cd52
Gmfg2	1.07E-68	1.427279	0.75	0.05	1.68E-64	12 Gmfg
Cd481	2.14E-65	1.052646	0.469	0.021	3.36E-61	12 Cd48
Ptprc2	1.57E-62	1.572341	0.719	0.051	2.46E-58	12 Ptprc
Selplg2	3.27E-61	1.59233	0.656	0.045	5.12E-57	12 Selplg
Limd22	3.27E-56	1.665559	0.75	0.069	5.13E-52	12 Limd2
Cd79b	7.00E-51	1.279269	0.125	0.002	1.10E-46	12 Cd79b
Cd79a	7.22E-51	1.704608	0.125	0.002	1.13E-46	12 Cd79a
Neurl32	1.22E-50	1.051693	0.469	0.027	1.91E-46	12 Neurl3
AW112010	1.68E-48	1.752983	0.594	0.048	2.64E-44	12 AW112010
Arhgdib4	2.25E-44	1.865686	0.844	0.111	3.54E-40	12 Arhgdib
H2-Q73	1.23E-43	1.802953	0.656	0.066	1.92E-39	12 H2-Q7
Fyb2	2.80E-41	1.502744	0.594	0.056	4.39E-37	12 Fyb
Ms4a6b1	1.85E-40	1.224015	0.375	0.022	2.90E-36	12 Ms4a6b
Fam107b3	2.74E-39	1.631336	0.656	0.074	4.30E-35	12 Fam107b
Dusp22	7.77E-39	1.187402	0.594	0.056	1.22E-34	12 Dusp2
1-Sep	3.12E-38	1.399363	0.562	0.055	4.90E-34	12 1-Sep
Vps37b1	1.69E-34	2.243775	0.812	0.148	2.65E-30	12 Vps37b
Srgn4	4.95E-32	1.051943	0.969	0.176	7.76E-28	12 Srgn
Crem1	8.59E-31	2.2617	0.875	0.203	1.35E-26	12 Crem
H2-Q62	2.89E-30	1.116413	0.469	0.046	4.53E-26	12 H2-Q6
Lcp12	4.65E-30	1.040123	0.531	0.056	7.29E-26	12 Lcp1
Ifi27l2a2	9.46E-30	1.259412	0.531	0.059	1.48E-25	12 Ifi27l2a
Apobec31	2.18E-29	1.005971	0.438	0.043	3.41E-25	12 Apobec3
Apbb1ip2	1.78E-28	1.234422	0.469	0.05	2.79E-24	12 Apbb1ip
Satb1	7.44E-28	1.234992	0.469	0.053	1.17E-23	12 Satb1
Ly6e4	8.02E-28	1.462281	0.844	0.153	1.26E-23	12 Ly6e
Ets11	1.72E-26	1.512068	0.688	0.128	2.70E-22	12 Ets1
Dusp53	4.30E-26	1.599444	0.625	0.102	6.75E-22	12 Dusp5
Samsn12	2.37E-25	1.123153	0.406	0.04	3.72E-21	12 Samsn1
Gramd3	3.87E-24	1.605239	0.562	0.091	6.06E-20	12 Gramd3
Ass13	6.31E-23	1.141823	0.531	0.075	9.90E-19	12 Ass1
Lfng2	1.29E-22	1.015627	0.344	0.033	2.03E-18	12 Lfng
Ctla2a2	1.48E-22	1.132514	0.375	0.039	2.32E-18	12 Ctla2a
Rps15a2	2.53E-22	1.316174	1	0.98	3.97E-18	12 Rps15a
Rpl173	6.71E-22	1.17077	1	0.98	1.05E-17	12 Rpl17
Psmb83	1.21E-21	1.141867	0.625	0.114	1.90E-17	12 Psmb8
Rps243	1.95E-21	1.116009	1	0.98	3.06E-17	12 Rps24
Rpl18a3	3.98E-21	1.10497	1	0.992	6.25E-17	12 Rpl18a
Tmsb103	4.25E-21	2.055268	1	0.854	6.67E-17	12 Tmsb10
Rps133	7.83E-21	1.184051	1	0.982	1.23E-16	12 Rps13
Tnfaip32	8.35E-21	1.132382	0.5	0.074	1.31E-16	12 Tnfaip3
Rpl324	8.92E-21	1.092709	1	0.981	1.40E-16	12 Rpl32
Rpl182	1.10E-20	1.025601	1	0.978	1.73E-16	12 Rpl18
Rps183	1.36E-20	1.234135	1	0.971	2.14E-16	12 Rps18
Rps72	1.61E-20	1.208886	1	0.968	2.53E-16	12 Rps7

Fau4	2.07E-20	1.152047	1	0.994	3.25E-16	12	Fau
Rps193	2.72E-20	1.094948	1	0.981	4.27E-16	12	Rps19
Rpsa2	3.42E-20	1.044457	1	0.968	5.37E-16	12	Rpsa
Rps33	4.84E-20	1.015179	1	0.983	7.59E-16	12	Rps3
Rplp23	7.48E-20	1.008122	1	0.985	1.17E-15	12	Rplp2
Rplp01	1.06E-19	1.04119	1	0.982	1.66E-15	12	Rplp0
Tgif12	1.70E-19	1.051305	0.594	0.116	2.67E-15	12	Tgif1
H2-K13	9.49E-19	1.5784	1	0.605	1.49E-14	12	H2-K1
B2m4	1.07E-18	1.340818	1	0.978	1.67E-14	12	B2m
Rpl113	1.19E-18	1.104575	1	0.971	1.86E-14	12	Rpl11
Rps284	2.04E-18	1.046942	1	0.979	3.20E-14	12	Rps28
Shisa54	4.53E-18	1.631834	0.938	0.598	7.10E-14	12	Shisa5
H2-D14	9.51E-17	1.437159	1	0.858	1.49E-12	12	H2-D1
Pabpc13	1.24E-16	1.287582	0.969	0.833	1.95E-12	12	Pabpc1
Hmgb22	2.07E-16	1.517668	0.656	0.182	3.24E-12	12	Hmgb2
Rps27rt4	5.83E-16	1.352325	0.938	0.738	9.15E-12	12	Rps27rt
Tuba4a1	2.72E-15	1.126086	0.594	0.156	4.27E-11	12	Tuba4a
Emb2	5.22E-15	2.031202	0.844	0.494	8.19E-11	12	Emb
Rpl12	5.55E-14	1.003068	1	0.956	8.70E-10	12	Rpl12
Rgcc3	1.09E-13	1.529795	0.531	0.134	1.71E-09	12	Rgcc
Ifngr13	1.13E-13	2.003119	0.812	0.433	1.77E-09	12	Ifngr1
Btg12	1.66E-13	1.672467	0.906	0.659	2.61E-09	12	Btg1
Rpl13a2	1.99E-13	1.099381	0.938	0.921	3.13E-09	12	Rpl13a
Cd822	8.69E-13	1.004526	0.562	0.166	1.36E-08	12	Cd82
Junb4	9.61E-13	1.643719	0.938	0.883	1.51E-08	12	Junb
Jpt12	2.26E-12	1.186617	0.75	0.347	3.54E-08	12	Jpt1
Stk17b2	2.77E-12	1.25703	0.75	0.347	4.34E-08	12	Stk17b
H2afz1	4.41E-12	1.362292	0.906	0.809	6.92E-08	12	H2afz
Lsp12	1.23E-11	1.163941	0.594	0.195	1.93E-07	12	Lsp1
Pnrc12	3.41E-11	1.381305	0.875	0.6	5.35E-07	12	Pnrc1
Zfp36122	4.02E-11	1.488749	0.75	0.424	6.30E-07	12	Zfp36l2
Zc3hav11	7.07E-11	1.126327	0.438	0.113	1.11E-06	12	Zc3hav1
Ikzf2	7.24E-11	1.113894	0.25	0.037	1.13E-06	12	Ikzf2
Pim12	2.11E-10	1.156153	0.688	0.28	3.31E-06	12	Pim1
Nop533	1.19E-09	1.168962	0.844	0.684	1.87E-05	12	Nop53
Ppp1cc	3.13E-09	1.291291	0.75	0.494	4.90E-05	12	Ppp1cc
Prrc2c	1.24E-07	1.016236	0.75	0.564	0.001942	12	Prrc2c
Rgs22	1.62E-07	1.477067	0.688	0.405	0.002543	12	Rgs2
6-Sep	7.38E-07	1.001715	0.5	0.224	0.0115748	12	6-Sep
Cited21	0.0016982	1.034337	0.469	0.329	1	12	Cited2

Table S5. List of highly expressed genes identified in big macrophage cluster (avg_logFC < -1)

	p_val	avg_logF(	pct.1	pct.2	p_val_adj
Apoe	8.96E-28	-3.66306	0.2	0.886	1.41E-23
Cxcl2	2.39E-25	-3.19965	0.386	0.89	3.74E-21
Lyz2	1.17E-24	-2.06747	0.471	0.943	1.83E-20
C5ar1	3.15E-19	-1.7832	0.014	0.669	4.95E-15
Clec4e	5.41E-19	-1.92721	0.143	0.735	8.48E-15
Clec4d	8.27E-19	-1.73938	0.1	0.71	1.30E-14
Cd14	4.73E-18	-1.92449	0.314	0.792	7.42E-14
Pla2g7	7.28E-18	-1.74379	0.014	0.637	1.14E-13
Hmox1	5.68E-17	-2.47796	0.086	0.665	8.91E-13
Thbs1	1.30E-16	-2.44571	0.057	0.637	2.03E-12
Lst1	2.44E-16	-1.275	0.486	0.829	3.82E-12
Tgfbi	4.04E-16	-1.38714	0.486	0.861	6.33E-12
Sod2	5.96E-14	-1.57378	0.314	0.698	9.34E-10
Fcgr3	6.72E-14	-1.25195	0.2	0.698	1.05E-09
Tlr2	9.98E-14	-1.34922	0.271	0.706	1.57E-09
Mafb	1.39E-13	-1.50857	0.043	0.551	2.18E-09
F10	2.38E-13	-1.41288	0	0.506	3.74E-09
Chil3	1.14E-12	-2.60289	0	0.486	1.79E-08
Hp	1.27E-12	-1.86207	0.014	0.502	1.99E-08
Ctsd	3.43E-12	-1.40582	0.229	0.637	5.37E-08
Cd44	6.15E-12	-1.101	0.486	0.808	9.64E-08
Ccl3	6.71E-12	-1.59919	0.071	0.543	1.05E-07
Msr1	1.24E-11	-1.12927	0.014	0.469	1.95E-07
Cybb	1.49E-11	-1.19113	0.386	0.714	2.34E-07
Ccl6	3.28E-11	-1.36633	0.314	0.698	5.14E-07
Ctsl	1.02E-10	-1.09728	0.243	0.624	1.60E-06
Nlrp3	1.34E-10	-1.10895	0.414	0.727	2.11E-06
Cebpb	1.45E-10	-1.27066	0.057	0.482	2.27E-06
Emilin2	1.49E-10	-1.06726	0.286	0.653	2.33E-06
Capg	1.54E-10	-1.07788	0.114	0.551	2.42E-06
Arg2	1.67E-10	-1.12511	0.029	0.449	2.61E-06
Ifi204	3.67E-10	-1.13052	0.143	0.535	5.76E-06
Fn1	8.21E-10	-1.52619	0.157	0.531	1.29E-05
Mgst1	1.38E-09	-1.10096	0.014	0.4	2.16E-05
Vcan	1.89E-09	-1.14409	0	0.38	2.96E-05
Selenop	2.26E-09	-1.30075	0.271	0.649	3.54E-05
Ifi207	2.96E-09	-1.05294	0.329	0.649	4.65E-05
Slpi	9.42E-09	-1.92698	0.029	0.388	0.000148
Il1a	1.80E-08	-1.24119	0.014	0.367	0.000282
Slc7a11	1.96E-08	-1.14708	0.286	0.629	0.000308
Gda	2.38E-08	-1.01207	0.157	0.498	0.000373
Ptgs2	2.76E-08	-1.55092	0.171	0.531	0.000433
Phlda1	3.51E-08	-1.04119	0.271	0.6	0.00055

Table S6. List of highly expressed genes identified in small macrophage cluster (avg_logFC > 1)

	p_val	avg_logF(	pct.1	pct.2	p_val_adj
Ccr7	3.71E-29	2.435666	0.514	0.012	5.82E-25
Cd209a	3.86E-25	2.078408	0.486	0.024	6.06E-21
Ccl22	1.36E-18	1.875889	0.314	0.004	2.14E-14
Klrd1	3.91E-50	1.777162	0.786	0.008	6.13E-46
H2-Eb1	1.84E-25	1.663969	0.943	0.478	2.88E-21
Syngr2	1.26E-31	1.648784	0.986	0.494	1.98E-27
Ramp3	1.22E-29	1.591814	0.657	0.065	1.91E-25
H2-Aa	1.53E-26	1.588326	0.957	0.51	2.41E-22
Clec10a	5.13E-16	1.585265	0.514	0.11	8.05E-12
H2-Ab1	6.87E-22	1.488791	0.929	0.563	1.08E-17
Tbc1d4	9.37E-27	1.35398	0.443	0.004	1.47E-22
Tmem123	7.22E-13	1.313325	0.743	0.335	1.13E-08
H2-DMb2	4.51E-30	1.237546	0.6	0.037	7.08E-26
Rogdi	2.91E-19	1.2262	0.614	0.118	4.56E-15
Csrp1	1.27E-12	1.100923	0.529	0.147	2.00E-08
Tmem176a	7.83E-13	1.086961	0.771	0.408	1.23E-08
Flt3	2.42E-33	1.056936	0.586	0.016	3.80E-29
Lsp1	1.59E-16	1.024206	0.943	0.555	2.49E-12
Grasp	1.95E-22	1.007619	0.571	0.073	3.06E-18
